# Integrated analysis of Wnt signalling system component gene expression

**DOI:** 10.1242/dev.200312

**Published:** 2022-08-15

**Authors:** Paula Murphy, Chris Armit, Bill Hill, Shanmugasundaram Venkataraman, Patrick Frankel, Richard A. Baldock, Duncan R. Davidson

**Affiliations:** 1 School of Natural Sciences, Department of Zoology, Trinity College Dublin, The University of Dublin, Dublin 2, Ireland; 2 MRC Human Genetics Unit, Institute of Cancer and Genetics, University of Edinburgh, Crewe Road, Edinburgh EH4 2XU, UK

**Keywords:** Wnt signalling, 3D imaging, Integrated analysis, Computational analysis

## Abstract

Wnt signalling controls patterning and differentiation across many tissues and organs of the developing embryo through temporally and spatially restricted expression of multi-gene families encoding ligands, receptors, pathway modulators and intracellular components. Here, we report an integrated analysis of key genes in the 3D space of the mouse embryo across multiple stages of development. We applied a method for 3D/3D image transformation to map all gene expression patterns to a single reference embryo for each stage, providing both visual analysis and volumetric mapping allowing computational methods to interrogate the combined expression patterns. We identify territories where multiple Wnt and Fzd genes are co-expressed and cross-compare all patterns, including all seven Wnt paralogous gene pairs. The comprehensive analysis revealed regions in the embryo where no Wnt or Fzd gene expression is detected, and where single Wnt genes are uniquely expressed. This work provides insight into a previously unappreciated level of organisation of expression patterns, as well as presenting a resource that can be utilised further by the research community for whole-system analysis.

## Abstract (precis)

**Summary:** A systematic analysis of integrated expression patterns of Wnt signalling pathway component-encoding genes and canonical pathway read-out, spatially mapped in 3D to mouse embryo models identifies co-expression territories.

## INTRODUCTION

The Wnt signalling system of cell–cell communication is ancient and fundamental to the construction of an organised animal body plan (Loh et al., 2016), and is proposed to have arisen concurrently with the metazoan lineage (Moroz et al., 2014; Ryan et al., 2013). Since the original dual discoveries of key roles for Wnt in development and in dysregulation during oncogenic transformation, it has more recently been shown to control cell differentiation within, and maintenance of, stem cell niches (Clevers et al., 2014). Spatiotemporally localised Wnt signalling plays a key role in patterning the primary body axis across very different body plans (Holstein, 2012) and is required for the establishment and healthy maintenance of organ systems from the central nervous system (CNS) to the kidney and gut (Noelanders and Vleminckx, 2017; Tian et al., 2018; Wang et al., 2018). Clearly, the spatiotemporal expression of genes that control Wnt signalling is of central importance.

The Wnt system is complex, with components encoded by several highly conserved multi-gene families; for example, there are 19 conserved Wnt ligand encoding genes in all mammals. Indeed, Wnts are unusual with respect to the high number of family members and paired paralogues, compared, for example, with the hedgehog family, with only three vertebrate members. FGFs are another example of a large family of signalling molecule-encoding genes present early in multicellular evolution, like Wnts; however, their classification into direct paralogous pairs is less clear (Itoh and Ornitz, 2011). Each Wnt is uniquely essential, as demonstrated by mutation analysis in the mouse and the level of sequence conservation between species. Ten genes encode frizzled (Fzd) receptors, which work together with a variety of co-receptors, such as Lrps, Ryk and Ror. Extracellular modulators of the pathways include secreted frizzled related proteins (Sfrps), Wif1 and Wise (Sostdc1). Intracellularly Wnts can trigger a number of different pathways, the best understood of which is the canonical/β-catenin-dependent pathway whereby stabilisation of β-catenin leads to gene expression changes in the responding cell; pivotal components include the Tcf/Lef transcription factors and β-catenin itself. Many other proteins interact at multiple levels in alternative pathways, influencing cellular outputs and this is rendered yet more complex through cross-talk between the pathways and other key signals, such as bone morphogenetic protein (Singh et al., 2018).

A central question is, why are there so many Wnt and Fzd genes in a single organism? Indeed, there are 12 sub-families of Wnt genes conserved across metazoans, with gene loss and duplication in particular lineages (Somorjai et al., 2018). One possibility is that the activities of different members have become segregated during evolution to function in different spatiotemporal contexts in the developing embryo. Here, we address this question by making detailed comparisons of comprehensive gene expression patterns using an approach provided by the Mouse Atlas Project that is based on the idea of mapping gene expression and other data onto a series of digital reference models of the mouse at successive stages of development (Davidson and Baldock, 2001). These reference models provide a framework in which the data can be interrogated computationally, integrated in a database with other gene expression data and, importantly, visualised in numerous ways to examine 3D spatial relations in an anatomical context (Armit et al., 2017). Crucially, spatial mapping onto an explicit coordinate model embryo enables exploration and analysis of the underlying molecular anatomy that is unbiased by anatomical interpretation based on histology, as has been demonstrated by a number of projects, including the comprehensive Allen Brain Atlas (Lein et al., 2007).

We previously reported comprehensive 3D expression of Wnt and Fzd encoding genes at E11.5 [Theiler stage (TS) 19] (Theiler, 1989; Summerhurst et al., 2008) and of Tcf/Lef transcription factors across time (Vendrell et al., 2009). Here, we extend this work using the Mouse Atlas approach to examine and compare RNA expression patterns of genes encoding Wnt ligands (19 genes), Fzd receptors (ten genes), Tcf/Lef transcription factors (four genes), Sfrp (five genes) and other modulatory proteins, Wif1 and Wise, as well as canonical pathway activity revealed through a reporter mouse line TCF/Lef:H2B-GFP (Ferrer-Vaquer et al., 2010). We studied three key stages when the body plan is being elaborated and various organ systems established: TS 15 [embryonic day (E) 9.5], 17 (E10.5) and 19 (E11.5). This approach can be used to pose questions not possible in any other way, such as where no Wnt expression is detected or where specific groups of components are co-expressed or relate to territories of canonical pathway read-out. We set out to test the hypothesis that Wnt and Fzd expression is a mosaic of domains in each of which only one or a few Wnts and Fzds are expressed. Our results also provide insight into the similarity and divergence of expression of different Wnt genes in the mouse, including the deployment of the more recently duplicated paralogous genes and the relation between Wnt pathway component gene expression and canonical pathway activity.

## RESULTS

### Integrated visualisation of Wnt pathway component gene expression patterns from E9.5 to E11.5 in the mouse embryo

**Fig. 1.**
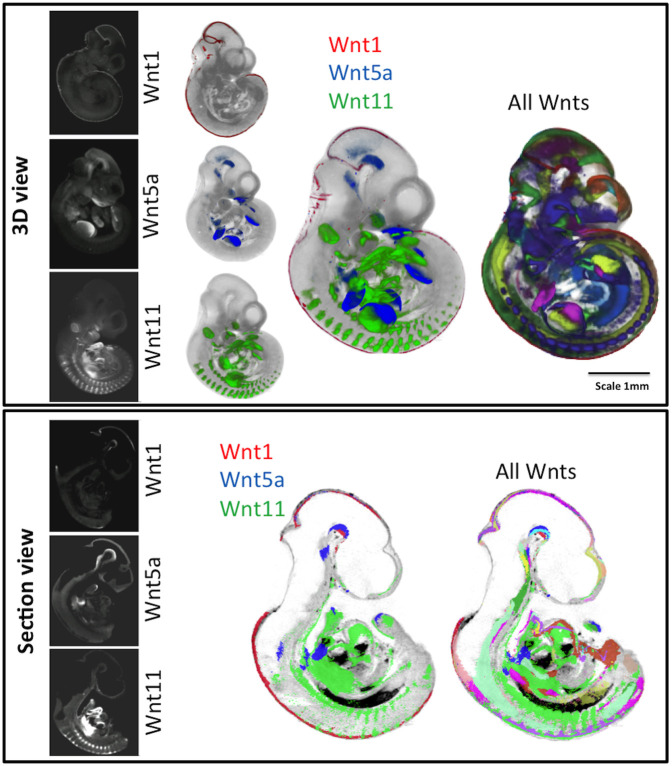
**Visualisation of mapped and integrated gene expression patterns, exemplified at E10.5.** Top: Whole embryo external views of 3D data. The original OPT reconstructions showing the expression of *Wnt1*, *Wnt5a* and *Wnt11* are on the left and individually mapped to the same reference model in the next column (see full 3D movies of each in Movies 1-3). The right-hand columns show the three patterns integrated and all Wnt expression patterns integrated (see 3D Movies 4, 5). Bottom: Virtual sagittal section views through the same 3D data, showing original OPT data and mapped data as above. Red, *Wnt1*; green, *Wnt11*; blue, *Wnt5a*. For more detail on visualisation of each pattern, see Movie 5.

3D expression patterns for all Wnt, Fzd, Tcf/Lef, Sfrp, Wif1 and Wise genes as well as canonical Wnt pathway read-out (Ferrer-Vaquer et al., 2010) were mapped using WlzWarp (Hill et al., 2022 preprint) onto reference embryos at E9.5, E10.5 and E11.5 (both data and reference embryos were staged precisely to TS 15, 17 and 19, respectively; Theiler, 1989). All data are available through University of Edinburgh DataShare (Murphy et al., 2021a,b) (https://doi.org/10.7488/ds/3141; https://doi.org/10.7488/ds/3142) and can also be viewed using an on-line 3D section viewer such as the Mouse Atlas IIP viewer (Armit et al., 2015; Husz et al., 2012; available at www.emouseatlas.org/WntAnalysis) or downloaded and viewed using ITK-SNAP (Yushkevich et al., 2006). Examples of mapped and integrated data are shown for Wnt genes (Fig. 1; Movies 1-5). The accuracy of mapping (Fig. S1) enabled an integrated analysis to reveal higher order patterns. Virtual sections through the original, unmapped optical projection tomography (OPT) data were examined on a gene-by-gene basis to complement this analysis, for example to distinguish epithelial-specific expression. Movies 1-3 show mapped domains of individual gene expression patterns at E10.5, shown in Fig. 1, and Movies 4 and 5 show the integration of three Wnt genes and of all Wnt genes added incrementally, respectively.

**Fig. 2.**
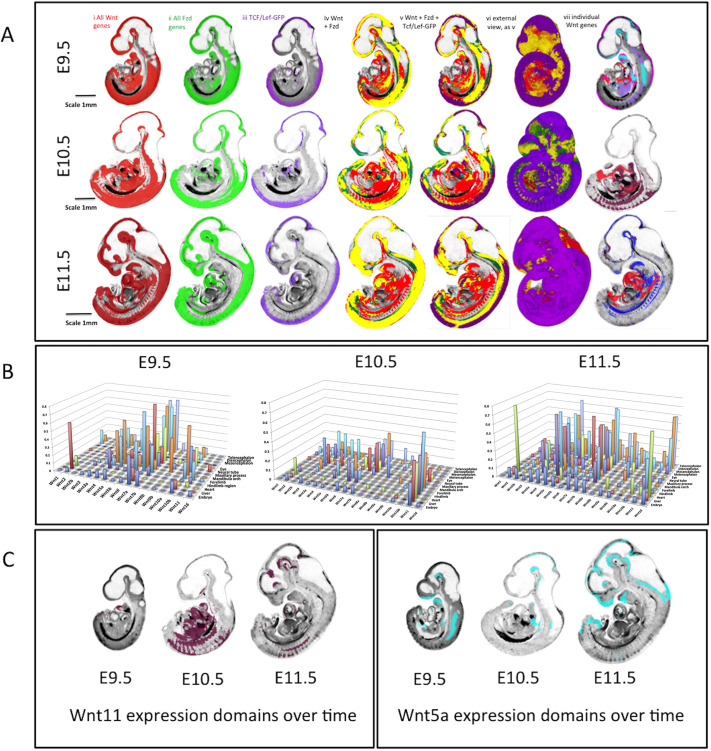
**Overview of integrated expression patterns.** (A) Union of the expression of all Wnt genes (red, i), all Fzd genes (green, ii) and the canonical pathway read-out reporter (purple, iii) across the three stages of development as indicated. Column iv shows the ‘all-Fzd’ domain overlaid on the ‘all-Wnt’ domain with overlap shown in yellow. Columns v and vi add the canonical read-out domain in purple (vi is an external view). Column vii shows individual Wnt expression patterns that contribute to the ventral Wnt domain (red, *Wnt2*; purple, *Wnt10b*; pink, *Wnt4*; pale blue, *Wnt5a*; dark red, *Wnt11*; dark blue, *Wnt5b*). (B) 3D graphs showing the extent (proportional size) of each Wnt gene domain in the whole embryo and in individual anatomical domains across stages as indicated; the *y*-axis shows the proportion of the anatomical domain (*z*-axis) occupied by each gene expression domain (*x*-axis). (C) *Wnt11* and *Wnt5a* expression domains on midline sagittal sections across stages; these patterns illustrate the dynamic changes in extent of expression across stages.

Fig. 2A shows combined domains for all Wnt ligand genes (red, i), Fzd receptor genes (green, ii) and canonical pathway read-out from the transgenic line TCF/Lef:H2B-GFP (referred to as Tcf/Lef-GFP throughout) (purple, iii) across stages. Overlay images of the Fzd and Wnt patterns show the overlap in yellow (iv). Across stages, combined Fzd domains are more restricted than combined Wnt domains, so some regions can be seen to express Wnt genes in the absence of detected Fzd expression (red, iv). These regions are extensive, largely ventral and visceral, and notably do not overlap with canonical pathway read-out (v). In contrast, domains that show detection of Fzd expression without Wnt expression (green, iv) are more restricted but with some notable examples, such as the anterior telencephalon at E10.5, where *Fzd3*, *6*, *7*, *8* and *10* are expressed, the ventral diencephalon at E10.5 and 11.5, and part of the developing limbs at E10.5, contributed to largely by *Fzd10*. As expected, canonical read-out, at any stage, largely fits within the overlap of Fzd and Wnt domains (v), in the dorsal aspects of the main body axis, prominent in the neural tube, and also in the limb, branchial arches and heart. We noted surprising instances in which Tcf/Lef-GFP is detected in the absence of concurrently detected Wnt or Fzd expression, notably the nasal epithelium at E11.5, proximal mesenchyme of the first branchial arch at E10.5 and E11.5 (Fig. S2) and regions in the ventral diencephalon at E10.5 and E11.5.

As all expression domains for each gene at each stage are digitally mapped, it is possible to quantitatively analyse the domains in the context of the whole embryo or within anatomical subdomains (Baldock et al., 2003; Brune et al., 1999) (Fig. 2B, Table S1). Therefore, one can computationally determine the proportion of the embryo, or of a delineated anatomical structure such as the neural tube, occupied by an expression domain over time. For example, at E10.5, the most broadly expressed Wnt gene in the embryo is *Wnt11* (22%), whereas *Wnt1*, *Wnt8a* and *Wnt8b* are very restricted (Fig. 2B; ≤1%). The extent of expression of Wnt genes is dynamic. Overall expression domains are more restricted at E10.5 compared with E9.5, becoming more expansive again at E11.5 (Fig. 2B), *Wnt5a* being a striking example (Fig. 2C), but there are notable exceptions, including *Wnt11*, *Wnt6*, *Wnt3*, *Wnt2* and *Wnt10a*, for which expression domains become expanded between E9.5 and E10.5, then become more restricted again at E11.5 (Fig. 2C).

**Fig. 3.**
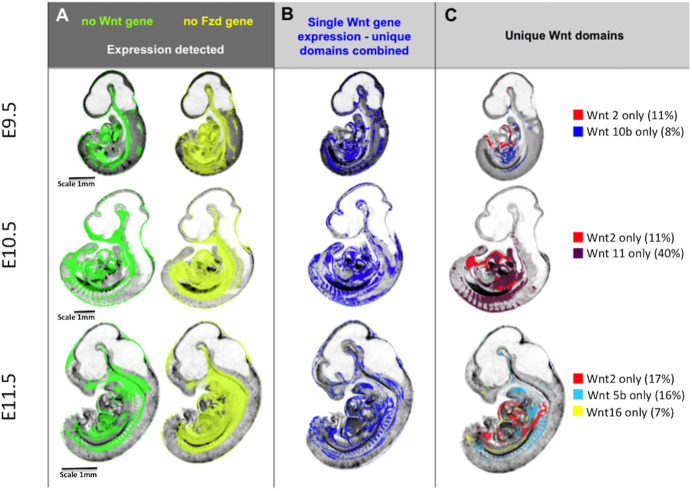
**Integrative mapping of all Wnt and Fzd domains allows visualisation of the territories where no Wnt or Fzd gene is expressed or where unique Wnt genes are expressed.** (A) Domains across stages where no Wnt expression (green) or no Fzd expression (yellow) is detected. (B) Domains across stages where a single Wnt gene is detected, i.e. unique detection of a single Wnt gene transcript. (C) Individual Wnt gene domains that account for much of the single Wnt gene domain at each stage, i.e. much of the unique Wnt gene territory in the ventral embryo at E10.5 is occupied by *Wnt2* and *Wnt11* expression domains, whereas *Wnt11* contributes little at E11.5 when *Wnt5b* is more prominent. The figures noted in brackets are the percentage of the unique Wnt gene expression domain at that stage contributed to by that gene. Note that the unique Wnt domains reported here were obtained by subtraction of multiple mapped expression domains and, as such, are sensitive to cumulative effects of noise in the data for each gene, in particular small differences in thresholding the original, continuously variable signals into binary (expressed versus not detected) values. Although the images show the general location of the domains, the boundaries should be considered approximate.

Comprehensive mapping shows territories in which no Wnt and Fzd expression is detected (Fig. 3A; see www.emouseatlas.org/WntAnalysis). These territories are largely ventral and visceral and are broadly similar across stages. Territories in which expression of a single Wnt gene is uniquely detected (Fig. 3B) are mostly represented in ventral, visceral regions, but also in parts of the nervous system, e.g. neural tube and diencephalon. These territories are similar between E9.5 and E10.5, but become noticeably more restricted at E11.5 (Fig. 3B). To determine which Wnts are expressed in these single-gene domains, we used parallel coordinate visualisation (Moustafa, 2011) across time, showing that a major contributor is *Wnt2* at all stages (8%, 11% and 17% of the combined single gene domain at stages E9.5, E10.5 and E11.5, respectively; representing 49%, 54% and 37% of the *Wnt2* domains, respectively; Table S2). Other major contributors are more dynamic (Fig. 3C). At E9.5 and E10.5, approximately one-third of canonical Wnt read-out domains are contained within unique Wnt expression territories, becoming about one-fifth at E11.5.

### Co-expression of multiple Wnt or Fzd genes

At the stages examined, most of the embryo displays the expression of up to two Wnt or up to two Fzd genes. However, certain localised regions express a notably large number of Wnt or Fzd genes where the expression of 4-11 Wnts or 4-8 Fzds maps to each image voxel in the reference model. We refer to the number of genes with expression mapped to a voxel as the ‘occupancy’ of that voxel, i.e. just one gene mapping to a particular voxel would be defined as occupancy level 1, two genes mapping to a voxel would be level 2, etc.

**Fig. 4.**
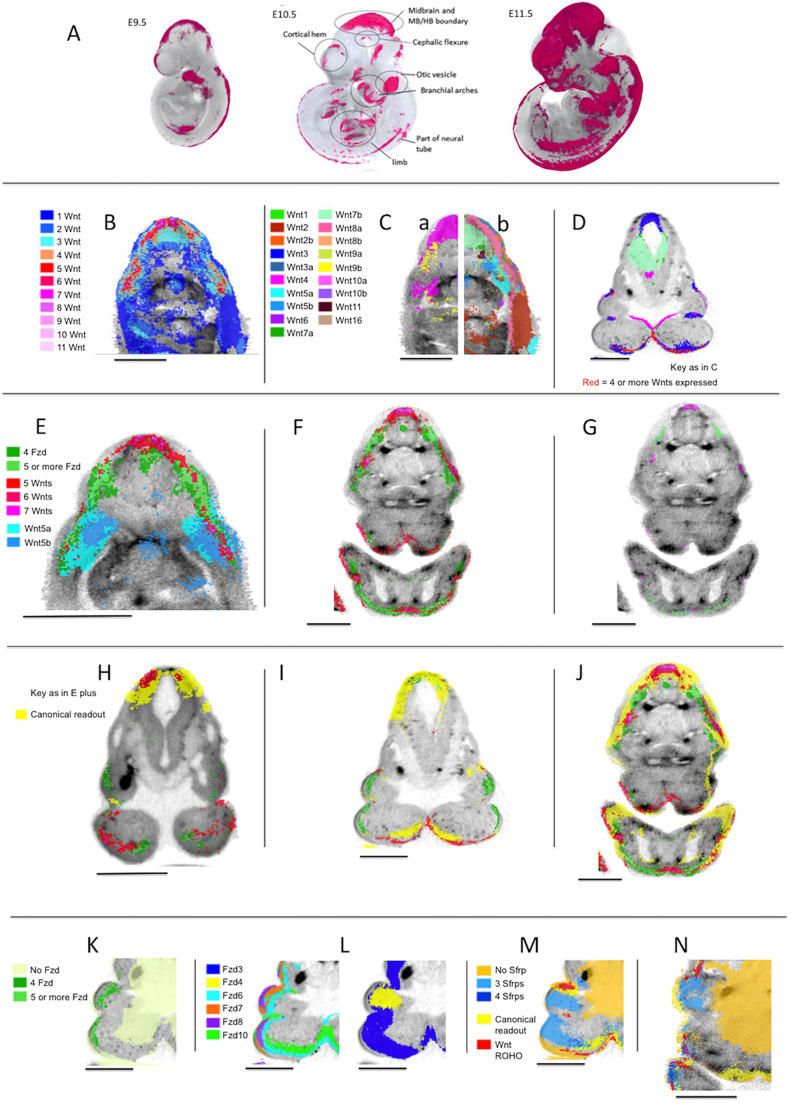
**Regions of high occupancy Wnt and Fzd expression.** (A) Regions of high occupancy of Wnt gene expression (magenta) at E9.5, E10.5 and E11.5. (B) Transverse section through an OPT reference model of an E11.5 embryo in the mid-flank region showing the distribution of occupancy of Wnt expression as indicated. This section reveals three ROHOs. (C) Same section as in B, showing the mapped expression of individual Wnt genes as indicated. For clarity, left (a) and right (b) halves of the section are shown with the expression of *Wnt3*, *4*, *8b*, *9b*, *10a*, *10b* (a) and *Wnt1*, *2*, *2b*, *3a*, *5a*, *5b*, *6*, *7a*, *7b*, *8a*, *9a*, *11*, *16* (b). (D) A section through an OPT reference model of an E10.5 embryo in the mandibular region. Regions of high occupancy of Wnt expression (red; four or more Wnts expressed) and individual Wnt gene expression domains are shown for *Wnt3*, *4*, *7a*, *7b*, *10a* and *10b* (key as in C). (E) Transverse section through an OPT reference model of an E11.5 embryo in the mid-flank region showing Fzd ROHOs (key as indicated) in the context of Wnt ROHOs (key as in B showing only 5+ Wnt genes) and expression of *Wnt5a* and *Wnt5b*. (F) Transverse section through an OPT reference model of an E11.5 embryo in the mandibular region showing Wnt and Fzd ROHOs (key as in E). (G) The same section as in F, showing only the peaks of Wnt and Fzd occupancy (Wnt occupancy of seven or more; Fzd occupancy of six or more. (H-J) Sections through the mandibular region in OPT reference models of embryos at E9.5, E10.5 and E11.5, respectively. The images show the canonical pathway read-out (Tcf/Lef-GFP RNA) (yellow) in the context of Wnt and Fzd ROHOs (key as in E). (K) Section through the mandibular region of an OPT reference model of an E10.5 embryo showing Fzd ROHOs compared with where no Fzds are detected (as indicated). (L) The same section as in K showing the expression of individual Fzds genes as indicated (the section is repeated for clarity). (M) The same section as in K showing Sfrp occupancy of 0, 3 and 4 (as indicated) in the context of Wnt ROHOs (key as in E) and canonical pathway read-out (Tcf/Lef-GFP) in yellow. (N) The same key as M on a section through the maxillary and mandibular region of an OPT reference model of an E11.5 embryo. Scale bars: 500 µm.

Generally, regions with high occupancy of Wnt or Fzd expression are distinct from one another (Fig. 4), each with one or a few peaks of occupancy (e.g. Fig. 4B, E11.5). At all three stages, Wnt occupancy levels 1 and 2 are widely dispersed, but most level 3 Wnt occupancy domains and almost every domain of occupancy level 4 and above, contains, or abuts, a region with occupancy level 5 or greater (Fig. 4B). In several locations, gradients of Wnt occupancy level 4 and above are steep. We have distinguished ‘regions of high occupancy’ (ROHOs) computationally as territories with occupancy above a certain threshold. For Wnt genes, we used five or more (5+) at E9.5 and E11.5 and 4+ at E10.5. For Fzd genes, we used 4+ at all three stages. ROHO territories have been analysed in detail (Fig. 4, Table S3).

In general, each ROHO defined in this way has a consistent location through successive stages (Fig. 4A). The number of Wnt ROHOs is approximately the same at E9.5 and E10.5 and increases at E11.5 when many Wnt ROHOs are more extensive and have higher levels of occupancy (Fig. S4C). Fzd ROHOs also become more extensive between E9.5 and E11.5.

Broadly speaking, Wnt ROHOs and Fzd ROHOs are localised to the same parts of the embryo, but not in all cases (Fig. 4E-I). In particular, many peaks of occupancy in Wnt ROHOs do not coincide with peaks in Fzd ROHOs, even at E11.5 when intersection between Wnt and Fzd ROHOs is maximal (Fig. 4F,G).

### The expression of individual Wnts in relation to regions of multiple Wnt expression

For most Wnt genes, expression is predominantly in domains that intersect Wnt ROHOs (compare Fig. 4C with 4B). However, the existence of Wnt ROHOs is not simply the result of the random intersection of large, unrelated expression domains. The Wnt genes most commonly expressed in ROHOs typically have small- to medium-sized domains that individually intersect discrete ROHOs and may extend through the adjacent epithelium or mesenchyme in a manner that appears to be localised around the ROHO (Fig. 4C-E). The ROHO-related expression of, for example, *Wnt3* at all stages (Fig. 4D) and *Wnt7a* at E11.5, is largely epithelial. Others, for example *Wnt5a*, have expression domains that include the epithelium of the ROHO and extend into the sub-adjacent mesenchyme (Fig. 4E).

For a few Wnts, e.g. *Wnt3* (Fig. 4D), most expression is ROHO-related. However, the majority have both ROHO-related and apparently non-related expression domains. *Wnt2* has extensive expression domains in the ventral trunk that display no consistent relation to ROHOs. *Wnt6*, although expressed in ROHOs, is expressed widely in surface epithelium and does not display convincingly localised expression in ROHOs. However, with the single exception of *Wnt16*, each Wnt displays at least one instance of local expression in a ROHO at one of the stages we examined.

For a selection of 36 Wnt ROHOs, we examined serial virtual sections to determine which genes have expression domains that intersect any voxel in the ROHO, referred to as the gene set for that ROHO (Table S3 shows ROHO location and gene set; W1-W36, counting ROHOs at each stage as separate). The number of ROHOs in which each Wnt is expressed and the distribution of the number of Wnts per ROHO are shown in Fig. S3A,C. It can be seen in Fig. S3A that *Wnt3*, *Wnt4*, and *Wnt10a* or *Wnt10b* are expressed in almost all the ROHOs we examined. In addition, though their expression is more widespread, *Wnt7a* or *Wnt7b*, or both, are expressed in 33/36 ROHOs. Thus, *Wnt3*, *4*, *7a*, *7b*, *10a* and *10b* are commonly expressed in Wnt ROHOs across the three stages (see, for example, Fig. 4D). *Wnt3a* could arguably be considered as a member of this common Wnt ROHO set at E9.5 when, strikingly, it is expressed locally and almost specifically in ROHOs.

Apart from the common Wnt ROHO gene set described above, the composition of expression in Wnt ROHOs is dynamic. As development proceeds through the stages we examined, there is a general increase in the number of Wnts expressed in ROHOs (Table S3, Fig. S3C). At E11.5, with only seven exceptions (*Wnt2/2b* in W16; *Wnt5a/5b* in W19; *Wnt8a/8b* in W13, W14, W21; *Wnt9a/9b* in W19, W20), each paralogous pair is represented by at least one member in each of the 14 ROHOs examined at that stage. There are some notable instances, for example, *Wnt10a* and *Wnt10b*, where the expression of a paralogue at one stage is apparently substituted by its partner at the following stage (Table S3).

### The expression of individual Fzds in relation to regions of multiple Fzd expression

Like the Wnts, most Fzd expression domains intersect Fzd ROHOs, for example *Fzd3*, *6*, *7*, *8* and *10* (Fig. 4L). Unlike Wnt ROHOs, Fzd ROHOs are characterised by the expression of most members of the gene family (Table S3, Fig. S3B). Five of the ten Fzds (*Fzd3*, *6*, *7*, *8*, *10*) are expressed in all or nearly all Fzd ROHOs, two (*Fzd1* and *9*) are expressed in more than half the Fzd ROHOs we examined and two (*Fzd4* and *5*) are expressed in about one-third of Fzd ROHOs. Thirty-one of the 39 Fzd ROHOs we examined express six or more Fzd genes (Fig. S3D).

### Wnt and Fzd ROHOs and canonical Wnt pathway activity

At all three stages, most Wnt ROHOs and Fzd ROHOs show at least partial intersection with Tcf/Lef-GFP activity. In some instances, the correlation is tight (for example the Wnt ROHOs in the distal forelimb and distal hindlimb at E10.5), but in the majority of cases Tcf/Lef-GFP activity, either partly or wholly intersecting the ROHO, extends beyond the ROHO (Fig. 4H-J). Some discrete domains of Tcf/Lef-GFP activity, although not intersecting any ROHO, lie in tissue immediately adjacent to one. One example is the isthmus (Table S3, W17, F17), where Tcf/Lef-GFP activity is absent in the flexure but present in the adjacent neural tissue. There are a few instances where Wnt and Fzd ROHOs do not display any apparent correlation with Tcf/Lef-GFP activity, for example in the mandible (Table S3, W2 and F2) at E9.5 (Fig. 4H). However, in these cases, Tcf/Lef-GFP is active at the subsequent stage, intersecting with the corresponding ROHO (Fig. 4I).

### Comparison of patterns: similarity and divergence of paralogous pairs of Wnt genes

**Fig. 5.**
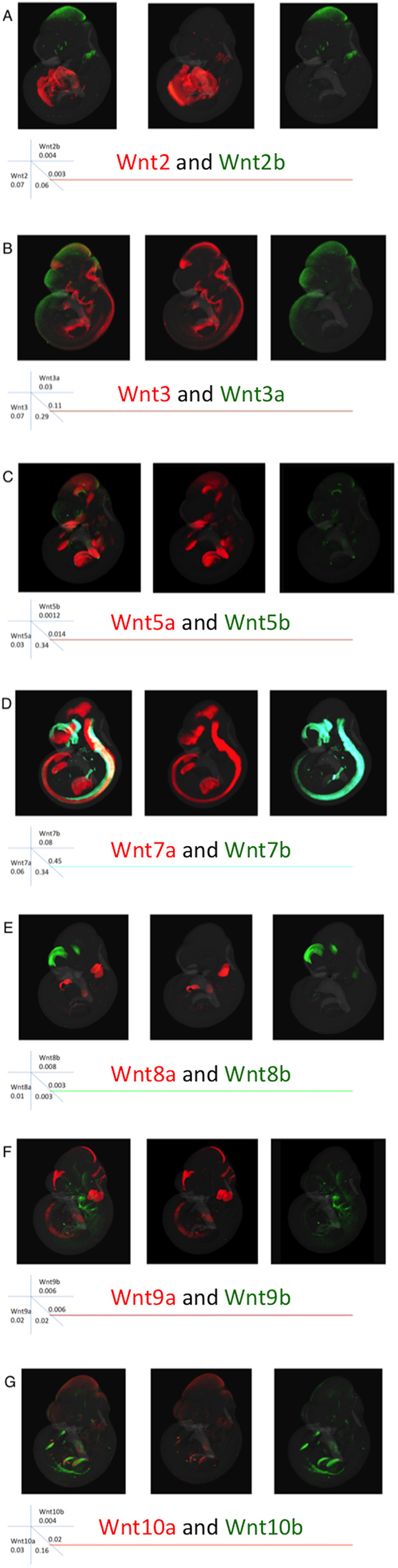
**Comparison of expression of paralogous pairs of Wnt genes at E10.5 mapped to the reference embryo model.** Each row shows a different pair of the seven Wnt paralogues, as indicated. The combined image of both genes is shown on the left and the two individual patterns in the order listed from left to right (colour coded). The rubric indicates the relative size of each domain (e.g. *Wnt2* occupies 7% of the embryo); the intersecting numbers show the proportion of one pattern overlapping the other so 6% of the *Wnt2b* domain overlaps the *Wnt2* domain. Note the highest level of overlap for the *Wnt7* paralogues, followed by the *Wnt3* and *Wnt5* paralogues.

We previously compared the expression of four pairs of Wnt paralogues (*Wnt2*, *5*, *7* and *8*) between mouse and chick embryos (Martin et al., 2012). Here, we compare seven pairs of paralogues (*Wnt1*, *3*, *5*, *7*, *8*, *9*, *10*) for overall similarity and divergence of expression in the mouse. Fig. 5 compares the whole embryo expression patterns at E10.5 and Table 1 shows the Jaccard similarity indices (JI) for each pair in the whole embryo (JI=volume of intersection/volume of union of the two domains). *Wnt7a* and *Wnt7b* show the greatest similarity of any pair of Wnt genes across all stages with extensive overlap in the CNS (Table 1, Fig. 5D, Table S4). Their expression patterns, however, show complementarity especially in the dorsal and ventral aspects of the forebrain (Fig. 5D). *Wnt7a* is more widely expressed than *Wnt7b* in the neural tube and limb. The *Wnt3* paralogues also show extensive similarity with the second highest JI at E9.5 among any Wnt gene pair and a high score across stages. Although distinct, extensive overlap in the midbrain is clear (Fig. 5B). There is also overlap in the posterior neural tube. The *Wnt10* paralogues are distinct at E9.5 (*Wnt10b* expression is extensive at this stage and more similar to other patterns) and most similar to each other at E10.5 and E11.5, particularly in the limbs, although the level of similarity is low for E11.5 (JI=0.06). *Wnt5* and *Wnt9* paralogues show increased expression similarity over time, whereas *Wnt5* genes become more divergent from other patterns in general. *Wnt2* and *Wnt8* paralogues show the most distinct patterns with weaker similarity indices than most other pairs of non-paralogous Wnt genes (Table S4).

**Table 1.**
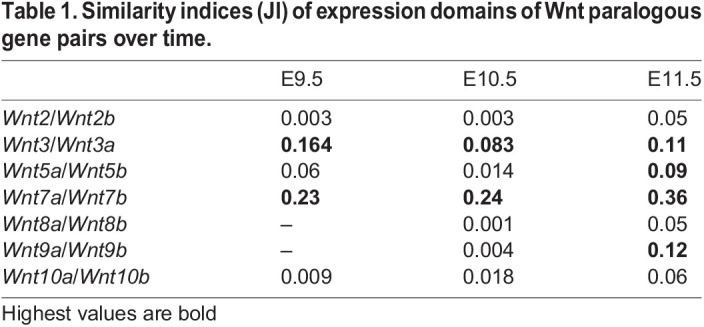
Similarity indices (JI) of expression domains of Wnt paralogous gene pairs over time.

### Comparison of patterns: all gene expression patterns and canonical pathway activity

**Fig. 6.**
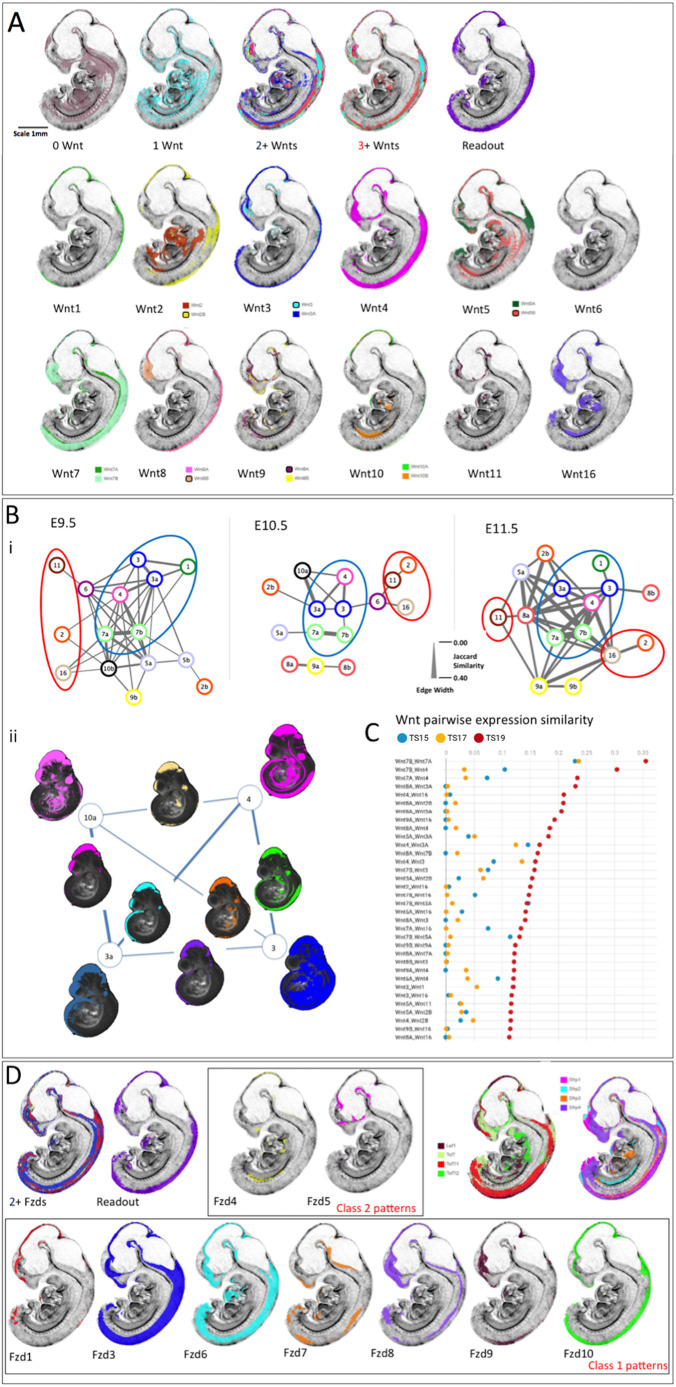
**Integrated comparison of gene expression pattern similarity across Wnt, Fzd and other pathway component genes.** (A) E11.5 example of the visual analysis carried out; all virtual sections are identical, mid-sagittal. The top row shows the territories where zero Wnts are detected (0 Wnt), where individual Wnt genes are expressed uniquely (1 Wnt), where 2 or more (2+ Wnts) or 3 or more (3+ Wnts) genes are co-expressed and where canonical Wnt pathway read-out is detected (Tcf/Lef-GFP). Rows 2 and 3 represent mapped expression of each of the Wnt family genes as indicated. The analysis included viewing the full set of sections in all orientations and across stages. (Bi) Network diagrams representing the similarities between Wnt expression pattern across time. The lines connecting nodes represent the JI of similarity (intersection/union), with thickness scaled as shown. Each ‘node’ represents a Wnt gene as indicated (e.g. 3a=*Wnt3a*). For comparison of the network, thresholds were adjusted to show the 15 genes with expression patterns most similar to other Wnt genes at each stage. Blue circles enclose the group with most highly similar expression patterns, consistent across stages (Group 1). Red circles enclose the most divergent expression patterns (Group 3). (Bii) Visual illustration of the nature of the lines connecting genes in the network focussing on the most highly connected genes at E10.5; *Wnt3*, *3a*, *4* and *10a*. The mapped Wnt expression patterns are shown here in projection through a 3D view of the reference model embryo, at each corner of the network (as indicated). Intersection domains, where each pair of expression patterns intersect, are shown on the lines connecting that gene pair. (C) Top 34 similarity scores among all Wnt genes across stages. The horizontal axis refers to JI (red, E11.5; yellow, E10.5; blue, E9.5). (D) Domains of multiple Fzd expression patterns (2+ Fzds) correspond well to territories of canonical pathway read-out (Tcf/Lef-GFP). Fzd expression patterns fall within two classes: class 1 (row 2) are similar to canonical read-out, Tcf/Lef transcription factor and Sfrp family member expression patterns (row 1, right). Example patterns at E11.5 are shown.

The expression domains of all genes and canonical read-out were examined for overlap using parallel-coordinate analysis, JI similarity (Table S4) and visual comparison. Fig. 6A shows striking similarity between where three or more Wnts are co-expressed and canonical pathway read-out, compared with where no genes are expressed or unique genes are expressed. Fig. 6A also illustrates each component gene expression pattern at E11.5. Table S4 shows the JI for each pair of genes. Plotting pairwise JI across stages (Fig. 6C), it is clear that Wnt gene family expression patterns generally become more similar to each other over time, with some notable exceptions (*Wnt1*, *6* and *10*). This is also the case when comparing across Wnt and Fzd gene families. For example, 50% of the *Wnt16* expression domain lies outside any Fzd expression domain at E9.5, but this drops to 13% at later stages. This is also reflected in the proportion of any pattern that is uniquely expressed (Table S2).

Wnt gene similarity indices were plotted as networks to compare the relationships between expression patterns across stages. For each stage, we plotted network graphs in which genes are presented as nodes connected by ‘edges’ with a line thickness that represents the similarity (JI) between expression patterns. Fig. 6B illustrates networks for the 15 Wnt genes that display the most similar patterns to other Wnts. The patterns can be divided into three groups as described below.

Group 1 consists of a core set of genes with expression patterns that show high JIs when paired (Fig. 6Bi, blue circled). This includes *Wnt7* and *Wnt3* paralogues, and *Wnt4*. *Wnt1* is also included at E9.5 and E11.5. These expression patterns are most similar to canonical read-out (Fig. 6A, Table S4). *Wnt4* changes somewhat over time; although consistently similar to *Wnt3*, *7* and *5a*, it also becomes more similar to other patterns (*Wnt8a*, *16*, *9a*, *2b*) at E11.5, largely owing to a new territory of forebrain expression (Fig. 6B).

Group 2 consists of *Wnt11*, *Wnt16* and *Wnt2*, which show low similarity across stages (Fig. 6Bi, red circled) and are among the genes with the largest proportion of unique expression (Table S2, Fig. 3C). Visual analysis shows that the patterns are distinct from canonical read-out (Fig. 6A). Both *Wnt11* and *Wnt16* show low similarity generally at E9.5 with some similarity to each other by E10.5 (JI=0.04). Both begin to be expressed in the brain at E11.5 driving increased similarity to other patterns at that stage; however, although *Wnt16* shares brain expression domains with more commonly expressed Wnt genes it is most similar to *Wnt2*, *Wnt9b* and *Wnt11* in the trunk.

Group 3 consists of the remaining gene expression patterns (Fig. 6Bi, uncircled), which show intermediate similarity that can vary across stages; at some stages they may be more similar to Group 1 genes. These include *Wnt5*, *Wnt8*, *Wnt9* and *Wnt10* paralogues, and *Wnt2b*. It is striking that both *Wnt8a* and *Wnt8b* patterns are most similar to *Wnt9a* at E10.5 although they show very low similarity to each other; *Wnt9a* shares different aspects of both patterns. *Wnt9a* and *9b* become more similar to each other and share the most expression territories with *Wnt16* at E11.5. *Wnt2b* becomes more typical of Group 1 patterns at E11.5. At E9.5, *Wnt5a* shows strong similarity to *Wnt7* paralogues whereas *Wnt5b* shows an intermediate pattern. However, *Wnt5b* expression becomes more distinct from other Wnt patterns with time; at E11.5 it shows some similarity with Group 1 genes largely through neural expression whereas it intersects *Wnt16* and *Wnt2* in the trunk. *Wnt10b* expression is extensive at E9.5 driving more similarity with Group 1 genes, but the pattern is overall very distinct. At E10.5, *Wnt10a* becomes more similar to Group 1, largely as a result of midbrain expression whereas *Wnt10b* is more similar to *Wnt2* and *Wnt16*.

The expression pattern of each Wnt gene generally comprises several sub-domains that are shared with some other Wnts but absent or much reduced in others. Thus, for example, at E10.5 Wnt expression in the dorso-lateral neuro-epithelium of the future telencephalon, in the anterior neural tube, mandible, branchial arches (grooves and pouches) and proximal limb comprises different combinations of genes. We examined the pattern similarities represented by the lines connecting pairs of genes in the network graph (Fig. 6Bi, edges) from this perspective. Fig. 6Bii presents a visual picture of edges in the network graph: in some instances, multiple connections to the same gene in the network reflect similar, though not identical, sets of intersecting expression domains (e.g. *Wnt3a* to *Wnt10a* and to *Wnt4* at E10.5), whereas, in other instances, different connections to the same gene reflect different combinations of intersecting domains (e.g. *Wnt4* to *Wnt3a* and to *Wnt3* at E10.5). Fig. 6Bii strikingly reveals that a domain centred on the midbrain is shared by all Group 1 genes.

Pair-wise comparison reveals that Fzd gene expression falls broadly into two pattern classes. Class 1 genes (*Fzd3*, *6*, *7*, *8*, *9*, *10*, and to a lesser extent *Fzd1*) show general overlap and similarity of expression across stages (*Fzd2* is not detected at any stage, *Fzd5* is not detected and *Fzd1* very little expression detected at E9.5; *Fzd9* expression is restricted largely to the brain) (Fig. 6D). Class 1 patterns also show general overlap with canonical pathway read-out [at E11.5, Fzd versus read-out JIs range from 0.26 (*Fzd10*) to 0.01 (*Fzd9*)], with extensive commonality with expression of the Tcf/Lef transcription factors and Sfrp modulators. In contrast, Class 2 patterns (*Fzd4* and *Fzd5*) show much less similarity with the canonical pathway read-out (JI=0.057 for *Fzd4* and 0.025 for *Fzd5*), each with distinct patterns. These different groups of Fzd patterns also overlap with different Wnt expression patterns, although the individual Wnts involved are dynamic over time. Whereas Class 1 Fzd patterns predominantly show similarity with Wnt Group 1, *Fzd4* and *Fzd5* both show greatest similarity to *Wnt9a* and *Wnt8a* at E10.5, with increased similarity with *Wnt16* (JI=0.124 and 0.149 for *Fzd4* and *Fzd5*, respectively) and *Wnt11* (JI=0.059 and 0.12) at E11.5.

Among Tcf/Lef transcription factor gene patterns, *Tcf7* and *Lef1* are most similar and *Tcf7l2* is the most divergent pattern across stages (Fig. 6D, Table S4). Sfrp gene expression patterns are dynamic, with *Sfrp4* more divergent at E10.5 but more similar, particularly to *Sfrp1* by E11.5 (JI=0.15 at E10.5 and 0.33 at E11.5) (Fig. 6D) (note that *Sfrp5* expression was not detected). *Sfrp1-4* patterns were also analysed visually for co-expression (Fig. 4M,N). Regions with co-expression of three or four Sfrp genes are generally well-defined and discrete. At E10.5, these regions rarely coincide with Wnt ROHOs, e.g. in the core mesenchyme and distal epithelium of the developing branchial arches (Fig. 4M). However, by E11.5, domains of multiple Sfrp gene expression intersect the expanding Wnt ROHOs, for example within, or close to, the surface epithelium in the developing face and limbs (Fig. 4N).

The Fzds show some interesting relationships to Sfrp patterns, but the Wnts are more complex and do not show consistent general relations with Sfrps. For example, at E10.5, the group of *Fzd6*, *7*, *8* and *10* show similarities of expression to *Sfrp2* in the face whereas *Fzd4*, *5* and *9* patterns display no apparent relation to *Sfrp2* expression. At E11.5, *Sfrp2* intersects *Fzd7* expression in interesting patterns in the face, limb and trunk, and *Sfrp4* intersects *Fzd7* in an interesting pattern in the limb.

### Detailed analysis of integrated expression patterns in the ventral diencephalon

**Fig. 7.**
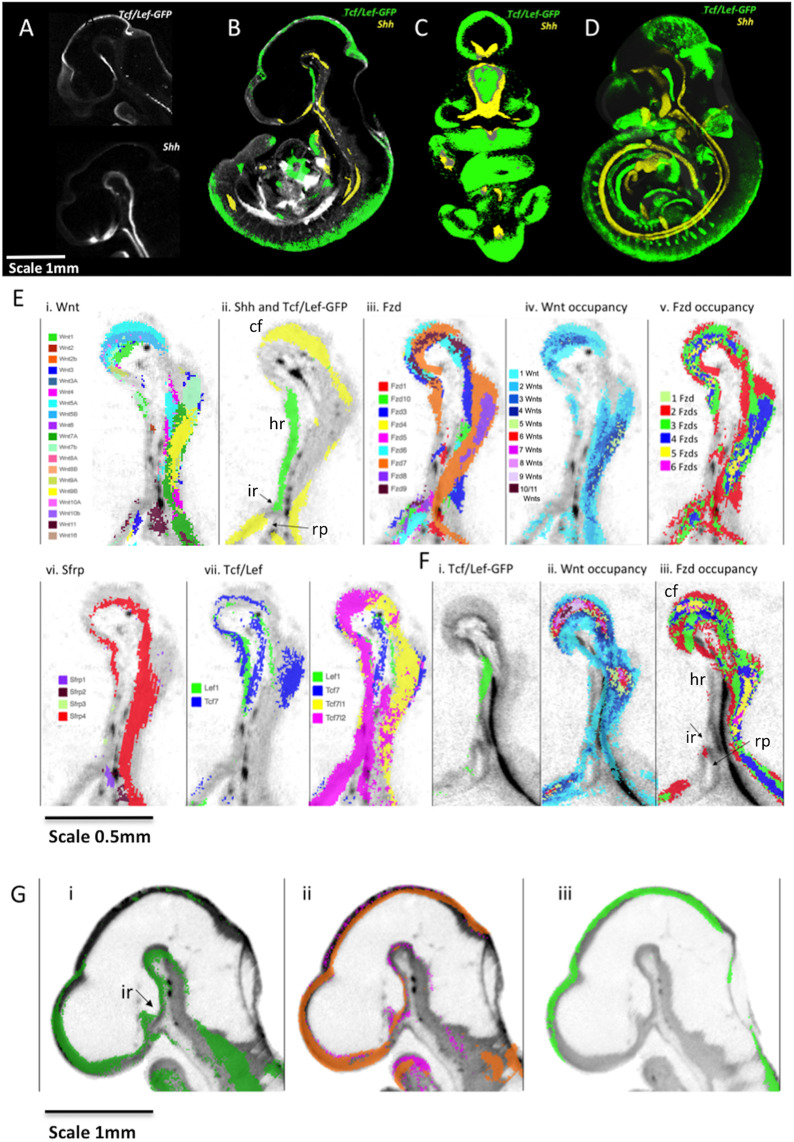
**Integrated expression patterns in the VD: complementary expression of Shh and canonical Wnt pathway read-out.** (A) Tcf/Lef-GFP read-out pattern and *Shh* expression in virtual sagittal sections through the diencephalon of raw OPT data. (B-D) Mapped data with greyscale render of the 3D model showing anatomy. (B) Virtual sagittal section through the 3D model emphasising the complementary expression of Tcf/Lef-GFP read-out pattern (green) and *Shh* (yellow) in the VD. (C) Thick virtual coronal section (288 µm) through the 3D model of mapped data again showing complementarity in the patterns (absence of overlap verified on serial sections). (D) Full 3D representation (point-cloud render). (A-D) E10.5. Scale bar in A also applies to B-D. (E) Sagittal sections through the VD at E10.5 showing: (i) mapped expression of all Wnt genes as indicated by the key; (ii) canonical Wnt read-out (Tcf/Lef-GFP, green) and *Shh* (yellow) expression on the same section; (iii) mapped expression of all Fzd genes as indicated by the key; (iv) Wnt occupancy; the number of Wnt genes co-expressed as indicated (no colour indicates that no Wnts are detected); (v) Fzd occupancy, with the number of Fzd genes co-expressed as indicated (no colour indicates no genes detected); (vi) mapped expression of Sfrp genes as indicated by key; (vii) mapped expression of Tcf/Lef transcription factor genes as indicated by key. (F) Sagittal sections through the VD at E11.5 showing (i) canonical Wnt read-out (Tcf/Lef-GFP, green); (ii) the number of Wnt genes co-expressed (key as in Eiv) and (iii) the number of Fzd genes co-expressed (key as in Ev) (no colour indicates no genes detected). (G) Sagittal sections through the brain at E9.5 showing (i) mapped expression of *Wnt7a* (green), (ii) mapped expression of *Fzd5* (magenta) and *Fzd7* (orange) and (iii) canonical Wnt read-out (Tcf/Lef-GFP, green). cf, cephalic flexure; hr, hypothalamic region; ir, infundibular recess; rp, Rathke's pouch.

Using the resource provided here, novel insights can be gleaned through focused analysis of any region of the embryo and used to build testable hypotheses. For example, examining the ventral diencephalon (VD) at E10.5 revealed a striking complementarity between Shh and canonical Wnt read-out (Fig. 7A-D). Tcf/Lef-GFP showed a gradient of expression through the midline of the VD that was strongest in the peduncular hypothalamus and the terminal hypothalamus caudal to the infundibulum. In particular, 3D rendered images of mapped data show that the Shh expression domain surrounds the Tcf/Lef-GFP domain (Fig. 7C).

We investigated which Wnt and Fzd expression combinations might drive expression of the Tcf/Lef-GFP reporter in the VD by digitally segmenting (Baldock et al., 2003; Brune et al., 1999) the VD anatomical domain in the E10.5 reference model and examining the mapped expression of genes in the Wnt signalling system using parallel coordinate analysis. We determined which genes are detected (1) where Tcf/Lef-GFP is active and (2) where Shh is expressed (Fig. S4). We then confirmed expression of these Wnt and Fzd genes in the VD neuroepithelium and Rathke's pouch through visualisation of mid-sagittal sections of both mapped and original 3D OPT data. Visual examination revealed that the region positive for canonical Wnt read-out shows limited detection of Wnt and Fzd expression at E10.5 (Fig. 7Ei-iii). Indeed, most of the region shows zero Wnt detection and there are only restricted regions of single Wnt genes: *Wnt2*, *Wnt4*, *Wnt7b* and *Wnt1* within the territory (Fig. 7Ei). Similarly, much of the region shows no detectable expression of Fzd genes; only *Fzd7*, *Fzd3* and *Fzd1* are expressed in restricted sub-regions (Fig. 7Eiii). In contrast, rostrally and caudally, where Shh is detected, multiple Wnts and Fzds are expressed (Fig. 7Eiv and v). For other members of the Wnt signalling system, only *Sfrp4* is expressed in the cephalic flexure and in the caudal VD but not throughout the rostral VD where the pathway is active (Fig. 7Evi). Among Tcf/Lef transcription factors, there is extensive expression of *Tcf7l1* and *Tcf7l2* whereas *Lef1* and *Tcf7* are restricted to the caudal VD (Fig. 7Evii). *Ror2* is expressed most strongly in the caudal VD (Fig. S4). In summary, canonical read-out is seen in the VD where Tcf/Lef transcription factors are expressed and Sfrp expression is restricted, but where, in a substantial part of the region, no Wnt or Fzd expression was detected. Shh is expressed where no canonical output is detected.

A day later, at E11.5, the region still has limited Wnt and Fzd gene expression, but canonical read-out is restricted more caudally (Fig. 7F). To investigate whether the activity at E10.5 could be due to earlier expression of Wnts and Fzds, we examined data at E9.5, which showed expression of a single Wnt gene, *Wnt7a*, and two Fzd genes (*Fzd5* and *Fzd7*) (Fig. 7G). Although we detected no canonical activity at E9.5, these pathway components could be involved in triggering later activity.

## DISCUSSION

Wnt signalling is one of the most studied sets of biological pathways, yet the challenge to understand the basis of spatial and temporal control of its biological outputs during development remains. Decades of work has described the expression of individual or small sets of genes in the vertebrate system, but the picture that emerges is patchy and driven by the focus of diverse studies. Here, we have used the Mouse Atlas approach to capture comprehensively the gene expression patterns of all Wnts, their Fzd receptors, Sfrp modulators, Tcf/Lef transcription factors as well as other interacting factors in mouse embryos in 3D over the developmental period when the patterning of different organ rudiments is being elaborated. Mapping the expression of different genes to common 3D digital models of embryos at each stage enabled an integrated spatiotemporal analysis of the patterns and comparison to a reporter (Ferrer-Vaquer et al., 2010) that reveals activation of one of several pathway outputs, the canonical pathway. This study provides insight into a level of organisation of the patterns that was not previously apparent, as well as presenting a resource that can be utilised further by the research community.

Our comprehensive approach indicated that the territories where no Wnt and no Fzd expression is detected across the three stages are largely ventral and visceral. Given the dynamism of the patterns, it is possible that a region where there is no detected expression at one stage might show expression at another; however, there is clearly some consistency in the regions that show no detected expression across the three stages assessed here. Territories in which the expression of only one Wnt gene was detected are also predominantly ventral, in particular expression of *Wnt2* across stages and *Wnt11* at E10.5 (Fig. 3C). Both genes show very divergent expression in our cross-pattern analysis (Fig. 6B), pointing to possible unique roles. They show mutant phenotypes in the placenta, kidneys and lungs that are consistent with these patterns (Goss et al., 2009; Majumdar et al., 2003; Monkley et al., 1996). Domains in which only one Wnt is expressed may also represent evolutionary diversification involving changes in gene regulation, more recently evolved territories of expression and neofunctionalisation (Ohno, 1970). By contrast, those domains with expression of multiple genes in all families are in dorsal and lateral regions, particularly the central nervous system, limbs, flank and face, consistent with the primary body axis being determined along the dorsal midline (see, for example, Arraf et al., 2016).

Not surprisingly, regions of the embryo show extensive overlap in domains of Wnt and Fzd expression and canonical read-out. In relation to the proximity of Wnt mRNA signal to sites expressing Fzd receptor RNA or canonical read-out, it is worth noting that in regions where *Vangl2* is co-expressed (largely the nervous system) active Wnt protein may be present in long cytoneme processes extending from Wnt-expressing cells so that the signalling activity may be distant from cell bodies expressing Wnt mRNA (Brunt et al., 2021).

To address the question of why there are so many Wnt and Fzd genes in a single organism, we have examined the hypothesis that Wnt and Fzd expression is a mosaic of domains, each expressing only one or a few members of these families. Our results are not consistent with this hypothesis. Some anatomical regions show the co-expression of one or a few Wnts, similar to what would be expected from this hypothesis, but there are many regions co-expressing strikingly large numbers of Wnts and Fzds. The focus of the question thus shifts to why are so many Wnts and Fzds co-expressed in these regions?

A striking finding is the co-expression of a large fraction of all Wnt or Fzd genes in localised regions of the embryo (termed ROHOs). These regions may reflect regulatory ‘hot spots’ for the gene families. Some coincide with known Wnt signalling centres, such as the isthmus and the cortical hem, but others have not been previously detected, for example the flank anterior to the forelimb (W5 in Table S4) and the ventral aspect where the forelimb meets the flank (W8 in Table S4). It is important to note that, generally, each gene has a distinctive expression pattern extending beyond the ROHO, often including domains unconnected to ROHOs. The intersection of patterns in ROHOs generally suggests spatiotemporal regulation centred on a small region of tissue. These observations open an avenue for investigating the signalling characteristics of these regions and their importance in patterning.

In terms of how ROHOs relate to canonical pathway activity, there is generally correspondence between Wnt and Fzd ROHOs and pathway read-out, but this is neither universal nor precise. The respective peaks of Wnt and Fzd ROHOs are usually offset. The same is true of regions of Tcf/Lef-GFP reporter activity. Even taking account of the possible involvement of cytonemes in signalling, this suggests that it is not simply the additive effect of multiple Wnt genes that activates the pathway; the relationship is more complex, reflecting the full regulatory landscape. Indeed, canonical signalling is not restricted to Wnt and Fzd ROHOs. We have quantified the extent of each expression domain and compared the territories in which different numbers of Wnt genes are co-expressed with canonical Wnt read-out showing that 33% of read-out falls within regions of unique Wnt gene expression at E9.5 and E10.5. We also found canonical Wnt pathway read-out in the absence of any detectable concurrent Wnt or Fzd gene expression (e.g. in nasal epithelium at E11.5 and the ventral diencephalon at E10.5 and E11.5). In the case of the VD, earlier (E9.5) expression of individual Wnt and Fzd genes could account for the later activity.

Another striking feature of all Wnt patterns is that they generally become more similar over the developmental time period covered (Fig. 6C); this is also reflected in the increased overlap of genes in ROHOs over time. Pairwise comparisons of similarity between expression patterns using the JI (Table S4) provides a view of the deployment of Wnt function that complements the co-expression analysis. Network analysis of pairwise comparisons (Fig. 6B) revealed three groupings of patterns. The first (Group 1) are similar across stages and often associated with regions of canonical pathway activity and Wnt ROHOs (Table S4; expression of *Wnt3*, *7* and *4* occurs in >30/36 ROHOs analysed) whereas the second (Group 2) are less associated with canonical activity and include the genes most expressed in unique territories and in ventral and visceral domains (*Wnt2* and *11*). A third group (Group 3) are intermediate and/or change their similarities over time, e.g. *Wnt10a* is very closely related to Group 1 patterns at E10.5 but dramatically less so at other stages. Group 1 expression patterns might lie closer to an ancestral pattern aligned with the primary body axis, whereas other patterns are more divergent and associated with recently added Wnt system functions, e.g. *Wnt2* association with placental development and *Wnt11* with kidney development. The predominant elements in the similarity between Group 1 patterns lie within the CNS including the dorsal midbrain, the cortical hem in the forebrain and the dorsal neural tube. Interestingly, further analysis of the similarity between Group 1 patterns (Fig. 6Bii) suggests that the expression of each of these genes comprises combinations of subdomains shared with some but not all members of the set indicative of modular regulation.

Turning to the question why so many Wnts and Fzds are co-expressed in localised regions, there are three non-exclusive possibilities: (1) convergent evolution of independent family members to satisfy a functional requirement for the expression of multiple genes, e.g. a threshold for Wnt ligand concentration; (2) conserved regulation of Wnts and Fzds, either active or passive; and (3) the existence of spatiotemporal regulatory control that spans the family, i.e. a form of ‘meta-regulation’ of the family. This raises the possibility that there exists a level of regulation, hitherto unknown, that directs the expression of the Wnts as a suite, and similarly for Fzds. One possibility, for example, would be positive-feedback regulation across the gene family.

We envisage that these possibilities apply not to the entire expression pattern of any gene, but rather to independently regulated sub-domains of expression. Indeed, there is no obvious simple relation between the net similarity of expression of pairs of genes represented in Fig. 6Bi and either their phylogenetic relationships based on DNA sequence (Somorjai et al., 2018), or certain widely conserved, tight chromosomal linkages (between *Wnt1* and *10b*; *6* and *10a*; *3a* and *9a*; *3* and *9b*; Ensemble genome browser https://www.ensembl.org/index.html). The potential for evolutionary conservation and shuffling of cis or trans regulatory modules, perhaps controlling different parts of each pattern (as, for example, in Fig. 6Bii), may be a fruitful area for future investigation (Marlétaz et al., 2018)*.* Interestingly, Wnt expression studies in amphioxus, which has 13 Wnt genes, also shows regions where multiple Wnts are expressed, for example posterior nested expression domains (Somorjai et al., 2018). This suggests that spatiotemporal intersection is not unique to the more complex gene family in the mouse. Evolutionary studies comparing amphioxus and the tunicate *Oikopleura dioica* have suggested three modes of evolutionary change in the Wnt gene family, namely conservation of function, function shuffling and gene loss (Martí-Solans et al., 2021). It is possible that the co-expression of subdomains of different Wnts in the mouse reflects the operation of a conserved ancestral regulation, though not necessarily conservation of precise gene function, across different Wnts.

We previously compared the expression of four pairs of Wnt paralogues within and between mouse and chick embryos (*Wnt2*, *Wnt5*, *Wnt7* and *Wnt8*) (Martin et al., 2012) showing evidence of greater divergence between subgroup paralogues than the respective orthologues, consistent with conserved subfunctionalisation/neofunctionalisation in the common vertebrate ancestor. Here, we compare all seven paralogue gene pairs reinforcing earlier observations and adding new insight. The JI shows that *Wnt7* and *Wnt3* paralogues are most similar of all Wnt pairwise patterns (Table 1, Table S4), yet the patterns are distinct, often complementary, in the same anatomical region. In contrast, *Wnt2* and *Wnt8* paralogues have diverged enormously in their expression characteristics, across all stages. *Wnt10* genes present an interesting case where they appear to ‘swap’ territories over time; especially evident in ROHO analysis where the same ROHO switches between expressing *Wnt10a* and *Wnt10b* (Table S3). These results add to our understanding of how paralogues arising by duplication of highly conserved genes evolve individually, sometimes maintaining aspects of their regulatory inputs while adjusting precise expression domains within that territory, and/or by acquiring new territories of expression. These findings present interesting contrasting cases (e.g. *Wnt7* versus *Wnt2*) to dissect the regulatory inputs for each gene pair to fully understand the regulatory changes involved.

In addition to the global analysis described above, our results can be used with a focus on individual organs and as a resource to complement hypothesis-driven approaches. As a case study, we analysed the VD in some detail. The VD goes on to form the hypothalamus and the neurohypophysis, which innervates the oral ectoderm-derived pituitary (adenohypophysis) through the infundibular stalk; together, these components form the hypothalamic-pituitary axis, of major importance in homeostasis. Initially using *Shh* as a marker gene, we immediately noticed a striking complementary pattern between *Shh* and the Tcf/Lef-GFP reporter (Fig. 7), suggestive of a repressive relationship between canonical Wnt signalling and *Shh* expression in this territory. Indeed Osmundsen and others (Camper et al., 2017; Osmundsen et al., 2017) have demonstrated that rostral expansion of β-catenin activity leads to coincident loss of *Shh* expression, elegantly demonstrating our original conjecture from visual analysis.

We further suggest a role for Wnt/β-catenin signalling in development of the neurohypophysis, in particular the evaginating infundibulum, and reveal Wnt signalling pathway gene expression patterns that could contribute to this important regulatory output. By digitally dissecting the territories that express *Shh* and those which show Tcf/Lef-GFP activity, coupled with parallel-coordinates visualisation, we could find potential regulatory inputs to the observed Tcf/Lef-GFP output, i.e. the cocktail of Wnts, Fzds and other regulatory components that are expressed in the region. Surprisingly few Wnts and Fzds are expressed in the region of Tcf/Lef-GFP activity, and none throughout the region at E10.5 and E11.5, whereas many genes are expressed in the *Shh*-positive territory where Tcf/Lef-GFP is not active. However, at the earlier stage of E9.5, *Wnt7a*, *Fzd5* and *Fzd7* are expressed in the region that later becomes Tcf/Lef-GFP positive. This cautions against drawing conclusions about pathway activity based on component gene expression patterns alone.

To explore the ventral diencephalon further, making use of the Mouse Atlas EMAGE database, we carried out a spatial query for genes with similar patterns to *Shh* and Tcf/Lef-GFP. This identified *Vax1* as having a complementary pattern to Tcf/Lef-GFP. Vax genes are of particular interest because they are known to inhibit canonical Wnt signalling through activation of an internal promoter transcribing a dominant-negative isoform of Tcf7l2 (Vacik et al., 2011). Furthermore, they are dependent on Shh signalling (Zhao et al., 2010), consistent with a mutually repressive relationship between Shh and Wnt signalling through expression of *Vax1*. We hypothesise that this Shh-dependent inhibition of Wnt/β-catenin signalling in the rostral VD is necessary to limit the pituitary-forming territory, consistent with ectopic pituitary formation in *Vax1*-deficient mice (Bharti et al., 2011).

The data reported here can be used to help direct future investigation of the global regulation and function of Wnt and Fzd family genes. In particular, it will be interesting to investigate the effects of manipulating individual genes on the expression and function of co-expressed members of the family. By providing a means to directly visualise comparisons between data in 3D and to incorporate retrospective and future data, the approach provides an opportunity to complement the data reported here with mutational and high-resolution, multiplex approaches (Lohoff et al., 2021).

## MATERIALS AND METHODS

### Gene expression and imaging

Expression patterns were generated by *in situ* hybridisation as previously described (Summerhurst et al., 2008) for *Sfrp1-4*, *Ror2*, *Wif1* and *Wise*, as well as previously reported Wnts, Fzds and Tcf/Lef genes. The cDNA sequences used for generation of RNA probes are detailed in Table S5. Read-out of the canonical pathway was revealed using GFP expression [both RNA *in situ* and anti-GFP immunofluorescence (Invitrogen, A11122, 1:200)] in a previously characterised transgenic mouse line (Ferrer-Vaquer et al., 2010).

3D imaging was carried out using OPT as previously described (Summerhurst et al., 2008).

Mouse embryos are referred to by embryonic day (E); however, embryos analysed were staged according to Theiler criteria (Theiler, 1989) to Stages 15, 17 and 19, here referred to as E9.5, E10.5 and E11.5, respectively. Expression patterns have been submitted to the Edinburgh Mouse Atlas of Gene Expression (EMAGE; IDs noted on Table S5), available at https://www.emouseatlas.org/emage/home.php. Patterns can also be viewed openly at https://www.tcd.ie/Zoology/research/groups/murphy/WntPathway/.

### Mapping of gene expression data

3D gene and reporter patterns were mapped onto reference embryos at each stage using a manual image-editing tool WlzWarp (Hill et al., 2022 preprint). This uses the ‘Constrained Distance Transform’ method (Hill and Baldock, 2015), which can deliver the complex non-linear transforms required for the variable shape and pose of mouse embryos. WlzWarp is an open-source tool (github.com/ma-tech) and provides interactive non-linear spatial mapping of 3D image data. This has been used for mapping significant volumes of gene expression data and tested by mapping multiple images of the same gene from independent samples (Hill et al., 2022 preprint). The process is straightforward, with the key required competence being an understanding of the biology and anatomy rather than technical IT expertise. More detail is provided by Hill et al. (2022 preprint), but the mappings for this data required operator time per embryo of about 60 min. Fig. 1 illustrates mapping examples. Mapping accuracy was assessed for each gene by comparing virtual sections of original 3D data (unmapped) against the mapped data (Fig. S1) showing good fidelity in most cases, which was reduced in some instances when mapping surface (ectodermal) expression (Fig. S1F).

### Analysis of integrated data

Primary checking and visual analysis of 3D expression data and mapped patterns used open-source tools MAPaint and MA3DView (github.com/ma-tech). For visualisation of mapped patterns, we used the IIP Viewer providing access to the data within a standard web-browser (Armit et al., 2015; Husz et al., 2012). The IIPViewer allows the user interactive selection of arbitrary section views through the mouse embryo and an overlay of all or any combination of the gene-expression patterns (available at www.emouseatlas.org/WntAnalysis). For convenience, we have included many of the derived patterns of multiple gene occupancy including regions where a single gene within a gene family is expressed.

In addition to this section-based visualisation, we provide a full 3D-rendered view using the ‘point-cloud’ approach, which delivers a volumetric style view of the entire pattern. Again, viewing any gene combination can be interactively selected including the entire gene set. The IIPViewer and point-cloud software and tools for generating the associated data are all open-source from the GitHub ma-tech repositories.

Analysis of mapped expression regions was undertaken using bespoke software tools based on the Woolz image processing system (Piper and Rutovitz, 1985; github.com/ma-tech/woolz). The tools are csh scripts that can be executed on any Unix-based system (e.g. Linux, Mac OSX) to generate all of the data values used for the downstream analysis. Specifically, they generate: (1) Tables of pair-wise intersection volumes as a count of the number of voxels in common between the two patterns normalised either by the test-pattern volume (row normalised) or the target pattern volume (column normalised). The overall volumes are provided to enable calculation of absolute volume values and using the voxel resolution these can be converted to real-space (μm^3^) values. (2) Tables of pair-wise similarity values using the JI (Levandowsky and Winter, 1971) based on the voxel set intersection and union volumes. (3) Volumetric domains of gene occupancy, which for a given gene set (e.g. Wnt) are calculated from the gene count, i.e. number of genes expressed at every voxel location within the embryo. This occupancy ‘image’ is then thresholded to define regions where, for example, there are five or more Wnts expressed at the same location. This can then be further analysed to reveal which genes are expressed within that region. Such occupancy data was used to reveal the regions of high occupancy (ROHOs) as well as regions of single gene occupancy where there is no overlapping expression within the gene family. (4) Re-formatted data for visualisation using the IIPViewer, point-cloud viewer and for the parallel-coordinate visual analysis using D3.js (d3js.org) Javascript visualisation library. (5) Re-formatted data for network analysis and input to Cytoscape.

All data required for these views are provided in a series of datasets held at the University of Edinburgh public data repository for Wnt Pathway Analysis (Murphy et al., 2021a,b; https://doi.org/10.7488/ds/3141; https://doi.org/10.7488/ds/3142). In addition, links to the parallel-coordinate views we have used are available at www.emouseatlas.org/WntAnalysis for convenience.

The network analysis software igraph (Csardi and Nepusz, 2006; igraph.org/) was used to construct networks according to the JIs of similarity across a variety of threshold levels across stages. The threshold level that showed the top 15 most similar genes at each stage was selected for detailed comparison and network visualisation using Cytoscape (Shannon et al., 2003; cytoscape.org) with the network layout unchanged.

## Supplementary Material

Supplementary information

Reviewer comments
