# Supplementary material for: Integrated analysis of Wnt signalling system component gene expression
Source: Development. 2022 Aug 15;149(16):dev200312. doi: 10.1242/dev.200312 (PMC9481969; doi:10.1242/dev.200312)
Supplement: Supplementary information [file develop-149-200312-s1.pdf]

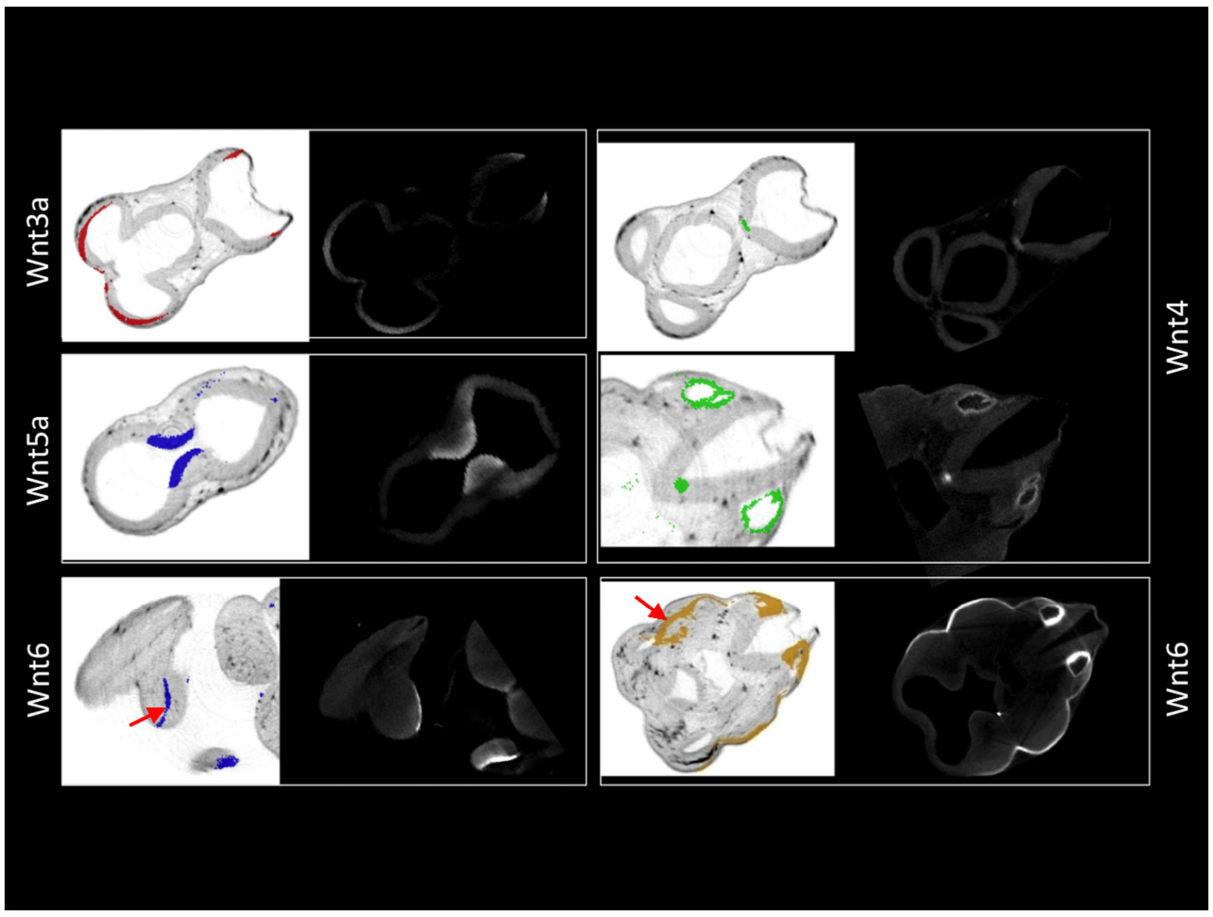

Fig. S1. Illustration of mapping accuracy using WlzWarp. Examples of mapped data (bright-field image on left) compared to equivalent virtual sections through 3D reconstructions of the original gene expression data (on right) for genes as indicated. All data are from E10.5 embryos through the forebrain and midbrain (Wnt3a, Wnt5a, Wnt4 (upper)), the hindbrain (Wnt4 lower), the forelimb (Wnt6 left) and hindbrain and forebrain (Wnt6 right). Note the mapping accuracy overall but the mis-mapping of ectodermal Wnt6 on one side of the embryo (arrows). Please note that mapped data are used for integrated analysis and any interpretation is supported by viewing the original scanned expression pattern. Relative scale as indicated on Figure 1.

Development • Supplementary information

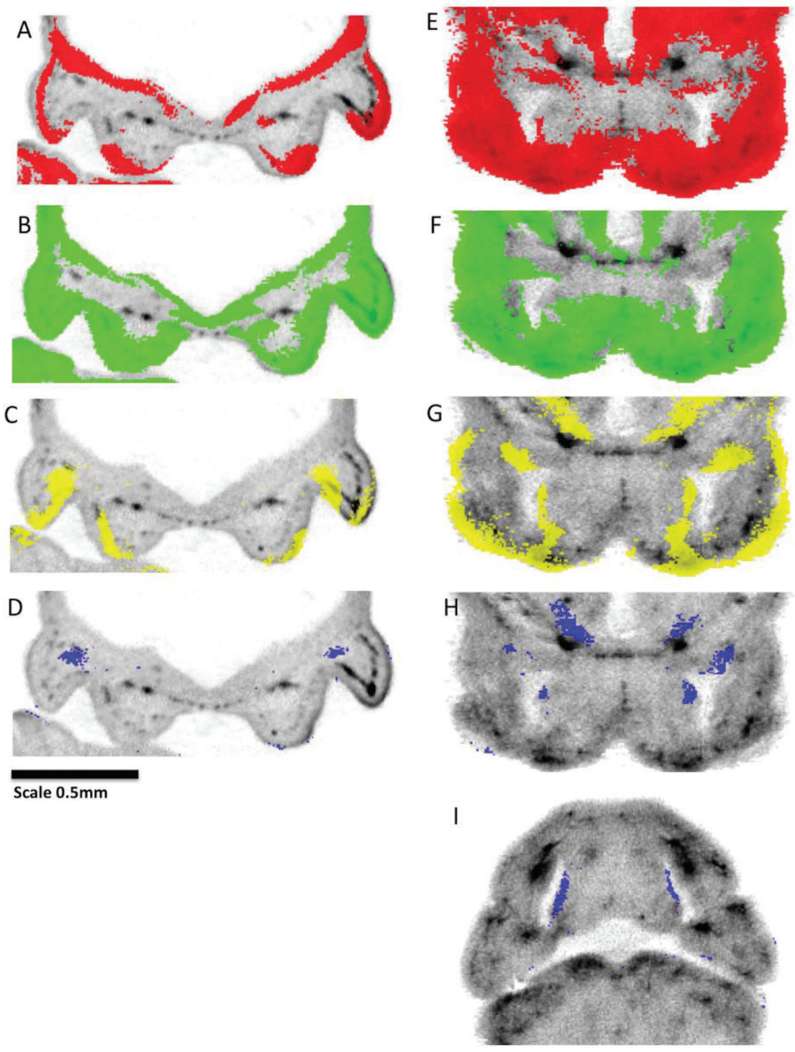

Fig S2. **Canonical Wnt pathway activity in the context of detectable Wnt and Fzd expression in the nasal region.** A-H: Frontal sections in the nasal region in OPT reference models of E10.5 (A-D) and E11.5 (E-H) embryos; I is a transverse section at E11.5. A and E, show combined expression domain for all Wnt genes; B and F, combined expression domain for all Fzd genes; C and G, Tcf/Lef-GFP; D, H and I shows the domain of Tcf/Lef-GFP activity where neither Wnt nor Fzd expression was detected.

Development • Supplementary information

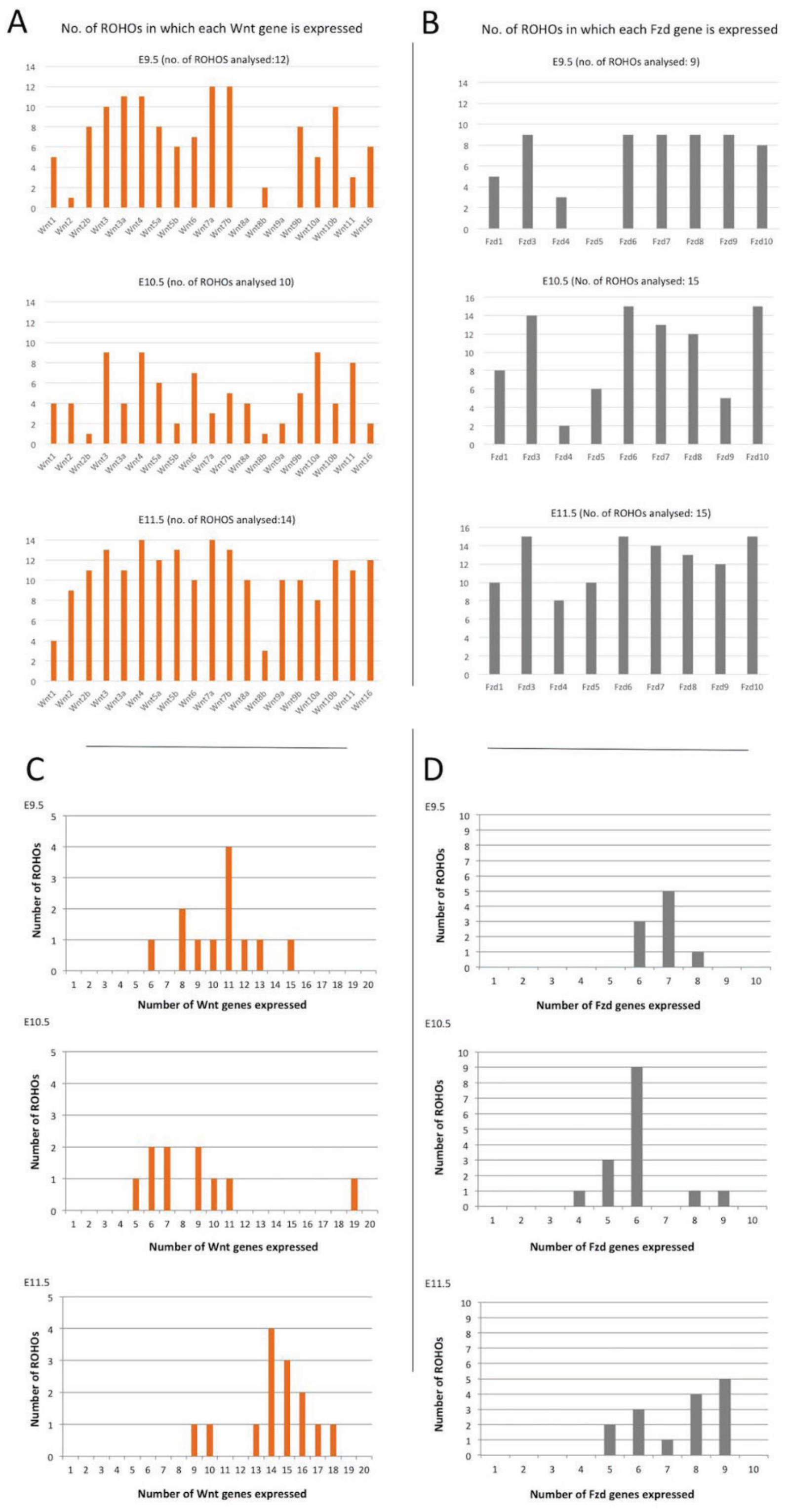

Fig. S3. Analysis of Wnt and Fzd ROHOs. A and B show the number of ROHOs in which each Wnt (A) and Fzd (B) gene are expressed, across stages. C and D graphically represent the number of Wnt (C) and Fzd (D) genes expressed per ROHO. Note the shift to more genes expressed per ROHO at E11.5

Development • Supplementary information

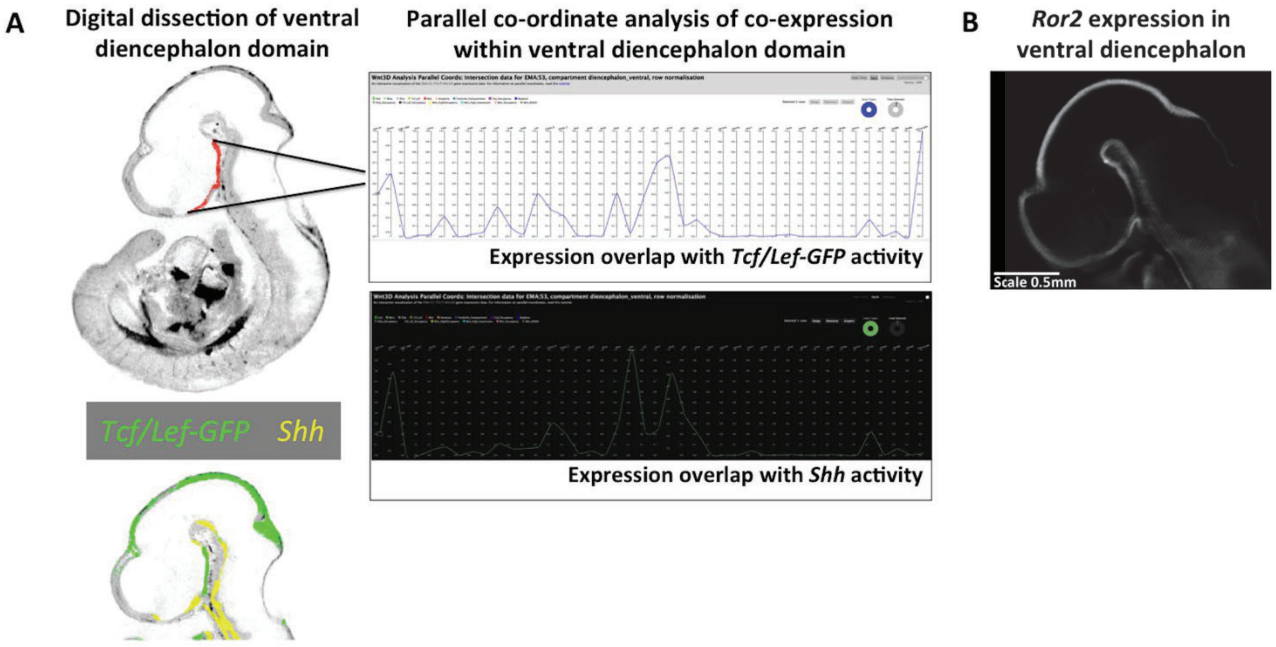

Fig. S4. Analysis of genes expressed in the ventral diencephalon. A represents the digital analysis of the full set of genes expressed using parallel co-ordinates of mapped data. B shows Ror2 expression in an OPT reconstruction.

Table S1. Proportional Wnt Expression Volumes

Gene	Embryo			Liver			Heart			Hindlimb Region		
	E9.5	E10.5	E11.5	E9.5	E10.5	E11.5	E9.5	E10.5	E11.5	E9.5	E10.5	E11.5
Wnt1	0.0183	0.0108	0.0171	0.0000	0.0001	0.0004	0.0005	0.0003	0.0035	0.0000	0.0000	0.0006
Wnt2	0.0498	0.0681	0.1134	0.5845	0.0389	0.0904	0.1287	0.1819	0.7694	0.0073	0.0625	0.0074
Wnt2b	0.0115	0.0035	0.1230	0.0001	0.0000	0.0000	0.0014	0.0001	0.0329	0.0091	0.0003	0.0186
Wnt3	0.0327	0.0714	0.0550	0.0018	0.0050	0.0002	0.0019	0.0214	0.0519	0.0525	0.0176	0.0147
Wnt3a	0.0705	0.0262	0.1417	0.0000	0.0000	0.0014	0.0001	0.0003	0.0009	0.0182	0.0090	0.3740
Wnt4	0.0849	0.0411	0.1567	0.0004	0.0067	0.0005	0.0140	0.0342	0.1991	0.2570	0.0024	0.0158
Wnt5a	0.1255	0.0298	0.1981	0.0035	0.0000	0.0002	0.0100	0.0027	0.0401	0.0587	0.0845	0.6995
Wnt5b	0.0228	0.0012	0.1110	0.0003	0.0000	0.0614	0.0039	0.0004	0.1450	0.0399	0.0011	0.0706
Wnt6	0.0619	0.0858	0.0262	0.0012	0.0000	0.0001	0.0037	0.0230	0.0064	0.1767	0.2243	0.1157
Wnt7a	0.3555	0.0614	0.1494	0.1613	0.0000	0.0063	0.0175	0.0002	0.0484	0.0223	0.1318	0.0268
Wnt7b	0.1863	0.0814	0.1547	0.0037	0.0024	0.0009	0.0099	0.0014	0.0059	0.1159	0.0000	0.0444
Wnt8a	0.0000	0.0099	0.1340	0.0000	0.0033	0.0104	0.0000	0.0400	0.0076	0.0000	0.0000	0.2404
Wnt8b	0.0054	0.0075	0.0199	0.0005	0.0000	0.0004	0.0013	0.0000	0.0011	0.0050	0.0000	0.0001
Wnt9a	0.0000	0.0188	0.0915	0.0000	0.0050	0.0042	0.0000	0.0447	0.1288	0.0000	0.0000	0.0062
Wnt9b	0.0241	0.0058	0.0595	0.0005	0.0019	0.0299	0.0068	0.0039	0.0172	0.0065	0.0002	0.0316
Wnt10a	0.0048	0.0287	0.0138	0.0002	0.0273	0.0001	0.0000	0.0133	0.0198	0.0001	0.0121	0.0006
Wnt10b	0.1359	0.0036	0.0236	0.1056	0.0001	0.0084	0.0416	0.0010	0.1765	0.0305	0.0269	0.0155
Wnt11	0.0177	0.2219	0.0580	0.0000	0.4035	0.0018	0.0550	0.3513	0.0953	0.0729	0.3941	0.1605
Wnt16	0.0603	0.0321	0.1576	0.0159	0.0000	0.0824	0.0161	0.0000	0.6911	0.0095	0.0002	0.0275

Gene	Forelimb			Mandibular Arch			Maxillary Process			Neural Tube		
	E9.5	E10.5	E11.5	E9.5	E10.5	E11.5	E9.5	E10.5	E11.5	E9.5	E10.5	E11.5
Wnt1	0.000	0.000	0.000	0.001	0.000	0.000	0.001	0.001	0.000	0.030	0.034	0.034
Wnt2	0.003	0.001	0.029	0.009	0.000	0.024	0.000	0.004	0.009	0.000	0.000	0.000
Wnt2b	0.006	0.000	0.093	0.005	0.000	0.061	0.006	0.005	0.101	0.007	0.000	0.259
Wnt3	0.041	0.054	0.028	0.010	0.112	0.035	0.043	0.317	0.038	0.038	0.218	0.075
Wnt3a	0.063	0.000	0.319	0.097	0.000	0.029	0.031	0.000	0.131	0.123	0.049	0.123
Wnt4	0.103	0.003	0.041	0.414	0.018	0.034	0.209	0.012	0.084	0.118	0.184	0.451
Wnt5a	0.391	0.157	0.704	0.563	0.011	0.193	0.003	0.000	0.522	0.112	0.005	0.057
Wnt5b	0.009	0.000	0.076	0.002	0.000	0.204	0.000	0.078	0.123	0.005	0.000	0.076
Wnt6	0.362	0.172	0.103	0.149	0.224	0.042	0.094	0.003	0.134	0.014	0.032	0.002
Wnt7a	0.736	0.097	0.047	0.533	0.000	0.036	0.272	0.006	0.020	0.748	0.252	0.615
Wnt7b	0.340	0.003	0.059	0.116	0.018	0.101	0.177	0.073	0.115	0.217	0.318	0.527
Wnt8a	0.000	0.000	0.373	0.000	0.004	0.068	0.000	0.002	0.168	0.000	0.001	0.151
Wnt8b	0.000	0.000	0.000	0.001	0.000	0.000	0.000	0.237	0.000	0.000	0.000	0.000
Wnt9a	0.000	0.000	0.001	0.000	0.000	0.003	0.000	0.000	0.008	0.000	0.001	0.017
Wnt9b	0.017	0.001	0.021	0.430	0.060	0.115	0.071	0.005	0.185	0.008	0.000	0.007
Wnt10a	0.001	0.023	0.004	0.000	0.007	0.000	0.000	0.000	0.001	0.006	0.007	0.009
Wnt10b	0.203	0.037	0.029	0.609	0.000	0.019	0.324	0.000	0.051	0.234	0.000	0.010
Wnt11	0.225	0.593	0.330	0.028	0.360	0.003	0.000	0.001	0.063	0.003	0.141	0.019
Wnt16	0.033	0.001	0.014	0.011	0.002	0.032	0.006	0.016	0.049	0.087	0.010	0.009

Gene	Eye			Metencephalon			MesenCephalon			Diencephalon			Telencephalon		
	E9.5	E10.5	E11.5	E9.5	E10.5	E11.5	E9.5	E10.5	E11.5	E9.5	E10.5	E11.5	E9.5	E10.5	E11.5
Wnt1	0.000	0.001	0.000	0.000	0.035	0.054	0.193	0.073	0.129	0.088	0.015	0.016	0.008	0.000	0.003
Wnt2	0.000	0.010	0.096	0.000	0.002	0.015	0.000	0.000	0.000	0.000	0.002	0.001	0.000	0.001	0.001
Wnt2b	0.007	0.017	0.206	0.000	0.002	0.046	0.021	0.088	0.199	0.016	0.007	0.005	0.005	0.000	0.016
Wnt3	0.009	0.040	0.003	0.000	0.168	0.131	0.252	0.293	0.337	0.042	0.138	0.254	0.001	0.001	0.206
Wnt3a	0.017	0.008	0.100	0.000	0.026	0.061	0.275	0.272	0.465	0.152	0.093	0.119	0.119	0.143	0.272
Wnt4	0.015	0.004	0.013	0.000	0.024	0.080	0.082	0.214	0.527	0.078	0.035	0.486	0.060	0.018	0.649
Wnt5a	0.003	0.000	0.025	0.000	0.020	0.253	0.297	0.234	0.327	0.139	0.036	0.197	0.012	0.000	0.169
Wnt5b	0.012	0.011	0.342	0.000	0.001	0.045	0.144	0.012	0.335	0.062	0.006	0.135	0.090	0.002	0.093
Wnt6	0.002	0.114	0.029	0.000	0.085	0.002	0.104	0.057	0.001	0.031	0.006	0.000	0.000	0.007	0.003
Wnt7a	0.343	0.000	0.005	0.000	0.198	0.439	0.517	0.238	0.441	0.620	0.131	0.385	0.673	0.222	0.312
Wnt7b	0.439	0.095	0.139	0.000	0.256	0.414	0.648	0.000	0.354	0.508	0.327	0.483	0.686	0.212	0.604
Wnt8a	0.000	0.001	0.140	0.000	0.010	0.141	0.000	0.001	0.642	0.000	0.001	0.249	0.000	0.000	0.155
Wnt8b	0.001	0.000	0.000	0.000	0.000	0.013	0.001	0.000	0.042	0.018	0.038	0.163	0.100	0.148	0.310
Wnt9a	0.000	0.000	0.030	0.000	0.027	0.126	0.000	0.048	0.227	0.000	0.014	0.359	0.000	0.073	0.222
Wnt9b	0.049	0.012	0.105	0.000	0.007	0.123	0.000	0.001	0.141	0.037	0.003	0.187	0.073	0.002	0.126
Wnt10a	0.032	0.038	0.000	0.000	0.033	0.003	0.002	0.304	0.188	0.026	0.085	0.006	0.015	0.082	0.097
Wnt10b	0.236	0.000	0.001	0.000	0.000	0.003	0.076	0.001	0.128	0.134	0.002	0.003	0.086	0.001	0.005
Wnt11	0.000	0.042	0.008	0.000	0.027	0.029	0.000	0.002	0.085	0.005	0.024	0.190	0.000	0.015	0.098
Wnt16	0.003	0.001	0.014	0.000	0.011	0.100	0.000	0.000	0.389	0.001	0.000	0.611	0.001	0.001	0.590

Table S2.

(i) Proportion of the single-Wnt gene expression domain occupied by each Wnt across stages

Wnt gene	9.5 dpc	10.5 dpc	11.5 dpc
Wnt1	0.00684	0.00861	0.00454
Wnt2b	0.00466	0.00073	0.08383
Wnt2	0.07945	0.10506	0.16993
Wnt3a	0.02398	0.01722	0.07222
Wnt3	0.01189	0.06084	0.00597
Wnt4	0.0538	0.02939	0.03607
Wnt5a	0.07124	0.02666	0.11043
Wnt5b	0.00835	0.00074	0.16437
Wnt6	0.03493	0.09375	0.00921
Wnt7a	0.39379	0.05526	0.07819
Wnt7b	0.10618	0.09457	0.03283
Wnt8a	0	0.0059	0.02262
Wnt8b	0.00159	0.0053	0.003
Wnt9a	0	0.01962	0.06795
Wnt9b	0.01084	0.00259	0.02925
Wnt10a	0.00186	0.02698	0.00243
Wnt10b	0.1139	0.00136	0.01015
Wnt11	0.01592	0.39775	0.02279
Wnt16	0.06071	0.0476	0.07415

(ii) Proportions of each Wnt gene expression domain (and canonical read-out) within the single Wnt expression domain (normalised by the individual Wnt expression domain volume).

Wnt	9.5 dpc	10.5 dpc	11.5 dpc
Wnt1	0.11466	0.28179	0.06599
Wnt2	0.48927	0.54252	0.37266

Development • Supplementary information

Wnt2b	0.1242	0.07291	0.16949
Wnt3	0.11151	0.29977	0.02701
Wnt3a	0.10426	0.23143	0.12668
Wnt4	0.1944	0.25136	0.05721
Wnt5a	0.17406	0.31449	0.13857
Wnt5b	0.11239	0.21475	0.36803
Wnt6	0.1731	0.3844	0.0875
Wnt7a	0.3396	0.31664	0.13011
Wnt7b	0.17472	0.40856	0.05277
Wnt8a	0	0.20901	0.04197
Wnt8b	0.09059	0.24741	0.03749
Wnt9a	0	0.3669	0.18468
Wnt9b	0.13795	0.15841	0.12216
Wnt10a	0.11904	0.3306	0.04382
Wnt10b	0.25689	0.13489	0.10676
Wnt11	0.27627	0.63064	0.09769
Wnt16	0.30858	0.52106	0.11702
**Canonical readout**	0.31377	0.35058	0.18978

Note that the domains represented in Supplementary Table 2 were obtained by subtraction of multiple mapped expression domains and, as such, are sensitive to cumulate effects of noise in the data for each gene, in particular small differences in thresholding the original, continuously variable signals into binary (expressed versus not detected) values. The volumes should therefore be considered as approximate.

Development • Supplementary information

Table S3. Genes expressed in each of the Wnt and Fzd ROHOs examined. Right hand columns show location with Wnt ROHO in red and Fzd ROHO in green

STRUCTURE	ROHO	E9.5 set	E10.5 set	E11.5set	Location	Wnt ROHO and Fzd ROHO
Maxilla	**W1**	2b, **3, 3a, 4, 6,** 7a, **7b, 9b**, 10b	2, **3, 3a, 4,** 5a, **6, 7b, 9b**, 10a, 11	2, 2b, **3, 3a, 4,** 5a, 5b, **6,** (7a), **7b,** 8a, 9a, **9b,** 10b, 11, (16)	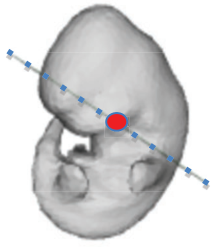	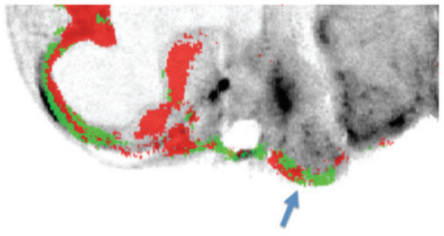
Maxilla	**F1**	**1**, **3**, 4, **6, 7, 8,** (9), (**10**)	(**1**), **3**, **6, 7, 8**, **10**	**1**, **3**, 4, **6, 7, 8**, (9), **10**		Location indicated above (in green)
Mandible	**W2** Postero-lateral	**(2b), 3, 3a, 4, 5a, 6, 7a, 7b, 9b, 10b, (16)**	NO ROHO	2, **2b, 3, 3a, 4, 5a**, 5b, **6, 7a, 7b,** 8a, **9b**, 10a, **10b**, 11, **16**	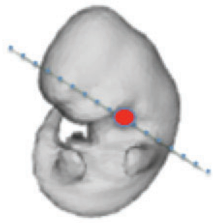	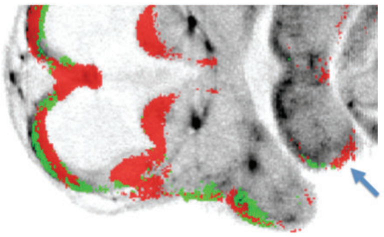
Mandible	**F2** Postero-lateral	(1), **3, 6, 7**, 8, 9, **10**	(1), **3, 6, 7**, 8, **10**	**3**, 4, **6, 7,** (9), **10**		Location indicated above (in green)
Mandible	**W3** Antero-medial	2b, **(3)**, 3a, **4, 5a, 6,** 7a, **7b, 9b**, 10b	**3, 4, 5a, 6, 7b,** 8a, **9b**, 10a, 11	NO ROHO	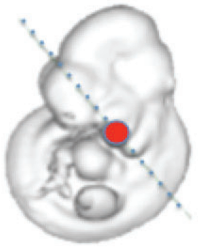	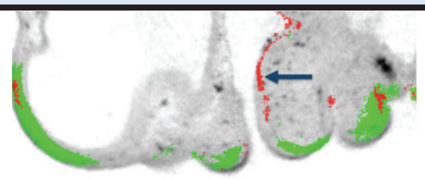

Development • Supplementary information

Mandible	**F3**<br>Antero-medial	**3**, 4, **6**, 7, 8, 9, **10**	1, **3**, **6**, **10**	NO ROHO		Location indicated above (in green)
Arch 2	**W4**	(2b), 3, 3a, 4, 5a, (5b), 7a, 7b, 9b, 10b, (16)	NO ROHO	NO ROHO	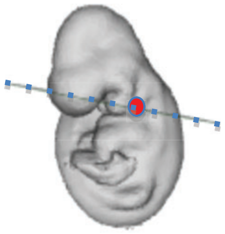	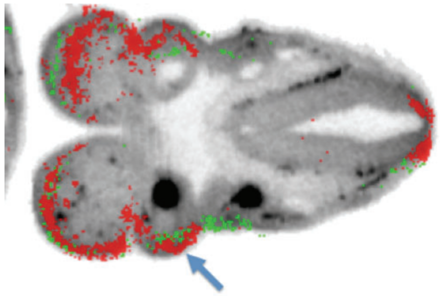
Arch 2	**F4**	(1), **3, 6, 7, 8**, 9, **10**	**3**, 4, **6, 7, 8, 10**	1, **3**, 4, (5), **6, 7, (8),** (9), **10**		Location indicated above (in green)
Neck	**W5**	No ROHO	**2, 3, (4), 5a, 6, 7a, 9b, 10a, 11**	**2,** 2b, **3,** 3a, **4, 5a,** 5b, **6, 7a,** 7b, 8a, 9a, **9b, 10a,** 10b, **11,** 16	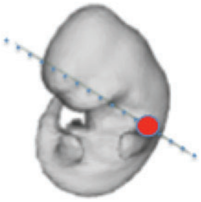	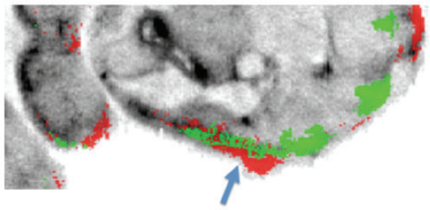
Neck	**F5**	**(1), 3,** 4, **(6), 7, 8, 9,**	**1, 3,** (5), **6, 7, 8, (9),** (10)	**1, 3,** 4, 5, **6, 7, 8, 9,** 10		Location indicated above (in green)
Fore Limb	**W6**<br>Early distal<br>E9.5 only	(2b), 3, 3a, 4, 5a, (5b), 6, 7a, 7b, (8b), 9b, 10a, 10b, 11, (16)			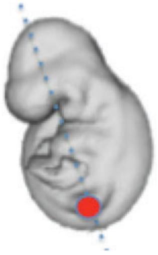	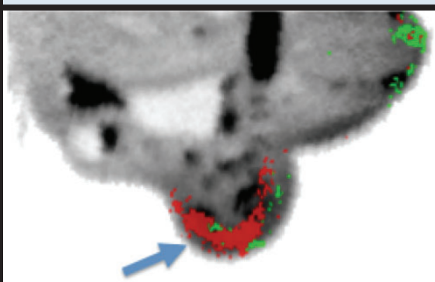
Fore Limb	**F6**<br>Early distal<br>E9.5 only	1, 3, 6, 7, 8, (9), 10				Location indicated above (in green)

Development • Supplementary information

Fore Limb	**W7**<br>Distal	N/A	5a, 6, 7a, 10a, 10b, 11	NO ROHO	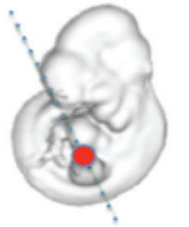	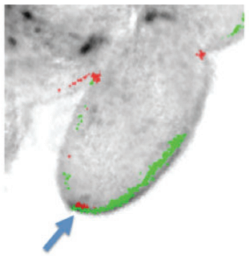
Forelimb	**F7**<br>Distal	N/A	continuous with ROHO F10 (5), 6, 7, 8, 9, 10	NO ROHO		Location indicated above (in green)
Forelimb	**W8**<br>Proximo-ventral	No ROHO	**2, 3, 4, 6, 7b**, **8a, 9a, 9b**, 10a, **10b, 11**	**2, 3**, 3a, **4**, 5a, 5b, **6,** 7a, **7b**, **8a, 9a, 9b, 10b, 11**, (16)	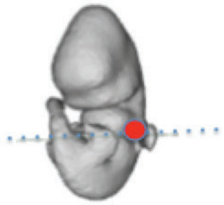	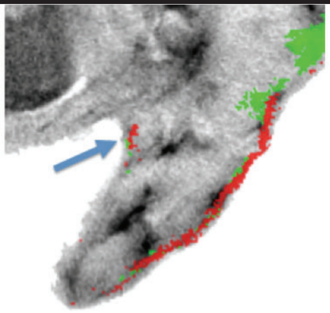
Forelimb	**F8**<br>Proximo-ventral	NO ROHO	**1, 3, 6, 7, 10**	**1, 3**, 5, **6, 7**, 8, **10**		Location indicated above (in green)
Forelimb	**W9**<br>Proximo-dorsal	NO ROHO	3, 4, 6, 10a, 10b, 11, 16	NO ROHO	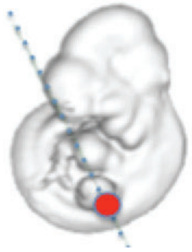	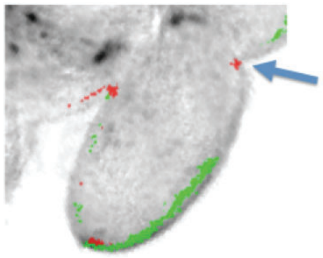
Forelimb	**F9**<br>Proximo-dorsal	NO ROHO	**1, 3, 6, 7, 8, 10**	**1, (3)**, 4, (5), **6, 7, 8, 10**		Location indicated above (in green)

Forelimb	**W10**<br>Dorsal surface	NO ROHO	NO ROHO	2, 2b, 3a, 4, 5a, 5b, 6, 7a, (7b), 8a, (9b), 10a, 10b, 11	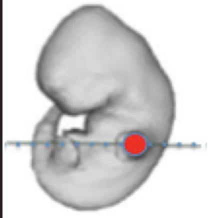	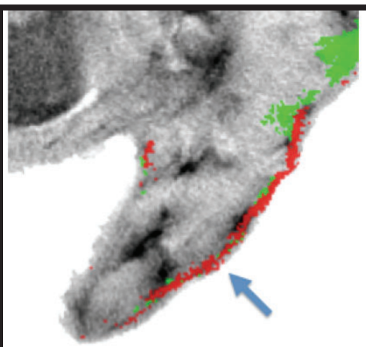
Forelimb	**F10**<br>Dorsal surface	NO ROHO	**3, 6, 7, 8, (9), 10**	1, **(3),** 4, 5, **6, 7, 8, 9, 10**		Location indicated above (in green)
Flank	**F11**<br>Flank dorsal to fore limb	NO ROHO	**1, 3, 6, 7, 8, 10**	**1, (3),** (5), **6, 7, 8,** (9), **10**	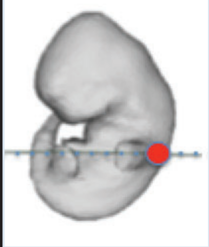	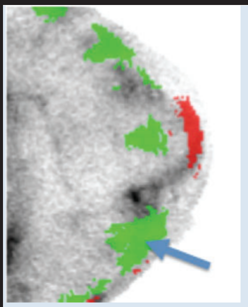
Flank	**W12**<br>Mid Flank	**2, (2b), 3, (3a), 4, 5a, 6, 7a, 7b, 9b, (10a), 10b, 11**<br>Small but distinct ROHO	NO ROHO	**2, 2b, (3**), **3a, 4, 5a**, 5b, **6, 7a, (7b)**, 8a, (8b), 9a, **9b, (10a)**, **10b**, **11**, 16	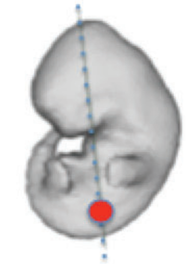	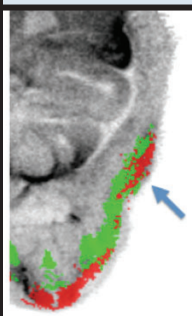
Flank	**F12**<br>Mid Flank	NO ROHO	**3, 6, 7, 8, 10**	1, **3,** (4), 5, **6, 7, 8,** 9, **10**		Location indicated above (in green)

Development • Supplementary information

Ventral tail	**W13**	**2b, 3, 3a, 4, 5a, 5b, (6), 7a, 7b,** 11, **16**	NO ROHO	2, **(2b), 3, 3a, 4, 5a, 5b, 6, 7a, 7b**, 9a, 9b, (10b), **16**	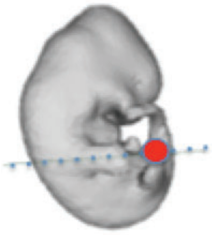	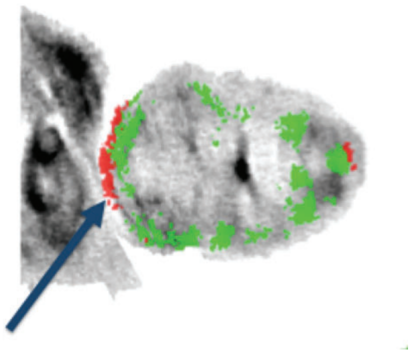
Ventral tail	**F13**	NO ROHO	NO ROHO	3, 6, 7, 9, 10		Location indicated above (in green)
Heart	**W14** Right atrium	NO ROHO	NO ROHO	1, 2 2b, 3, 4, 5a, 5b, 6, 7a, 7b, 9a, 9b, 10a, 10b, 11, 16.	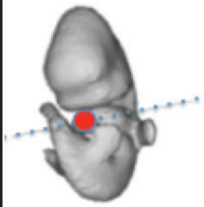	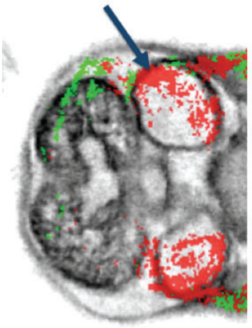
Heart	**W15** Left atrium	NO ROHO	NO ROHO	2, 3, 4, 5a, 5b, 6, 7a, 7b, 8a, 9a, 10a, 10b, 11, 16	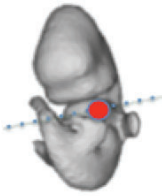	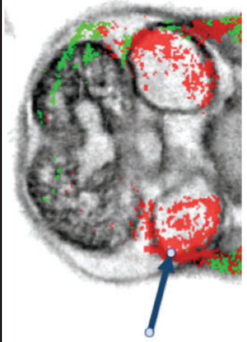

Development • Supplementary information

Cortical hem	**W16**	(1), **3a, 4, 5b, 7a, 7b, 8b, 9b,** 10a 10b, **(16)**	NO ROHO	3,**3a, 4**,5a,**5b,7a,7b**,8a,**8b**,9a , **9b,** 11,**16**	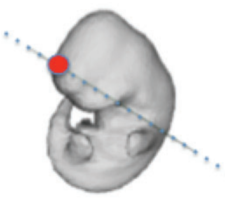	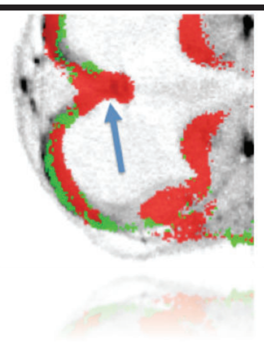
Cortical hem	**F16**	NO ROHO	NO ROHO	3, 5, 6, 8, 9, 10		Location indicated above (in green)
Isthmus	**W17**	**1, 5a, 5b,** 7a, **7b,** 10b	**1,** 3, (3a), 4, **5a, 5b, 7b**	**1,** 2b, 3, 4, **5a, 5b,** 7a, **7b,** 8a, 8b, 9a, 9b, (10b), 11, 16	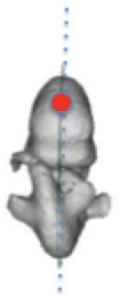	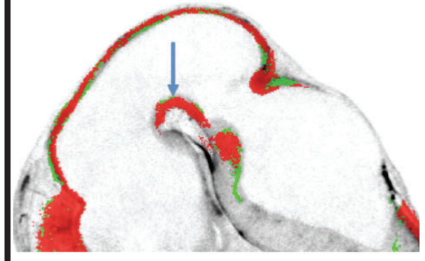
Isthmus	**F17**	NO ROHO	**3, (5), 6, 7, 9, 10,**	1, **3, 5, 6, 7**, 8, **9, (10)**		Location indicated above (in green)
Midbrian	**W18**	**1, 2b, 3, 3a, 4, (5a), 5b, 6, 7a,** 7b, **10a, 10b**	**1,** (2), **2b, 3, 3a, 4, 5a, 5b, 6, 7a,** 7b, 8a, 8b, 9a, 9b, **10a, 10b**, 11, 16,	**1, 2b, 3, 3a, 4, 5a, 5b 6, 7a,** 9a, **10a, 10b** (11), 16	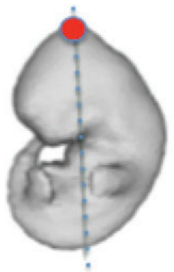	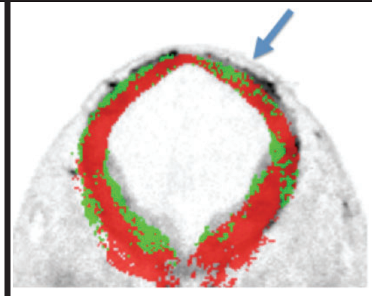
Midbrian	**F18**	**3, 6, 7, 8, 9, 10**	1, **3,** 4, 5, **6, 7, 8, 9, 10**	1, **3,** 4, 5, **6, 7, 8, 9, 10**		Location indicated above (in green)

Development • Supplementary information

Dorsal anterior NT	**W19**	**1, 3,** 3a, **4**, 7a, 7b, 10b, 16	**1, 3, 4**, (8a), 10a	**1**, 2b, **3**, 3a, **4**, 7a, 7b, 8a, 10a, 16,	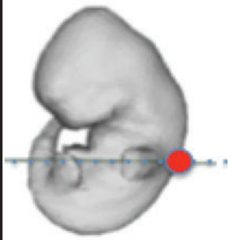	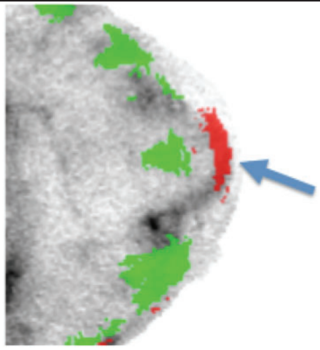
Dorsal anterior NT	**F19**	**3, 6,** 7, **8**, (9), **10**	**3,** (5), **6, 8, 10**	NO ROHO		Location indicated above (in green)
Mid-d-v anterior NT	**F20**	NO ROHO	NO ROHO	3, 6, 7, 8, 10	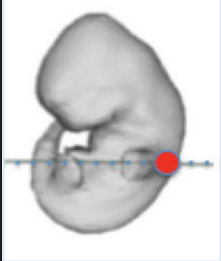	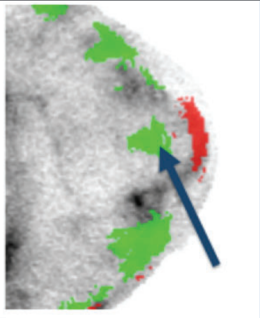
Dorsal trunk NT	**W21**	v. small ROHO **1, (3)**, 3a, **4**, 7a, 7b, 9b, (10a)	**1**, **3**, 3a, **4**, 10a, 11	**1**, 2b, **3**, 3a **4**, (5b), 7a, 7b, 10b	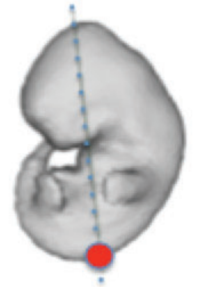	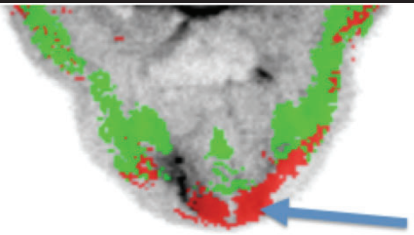
Dorsal trunk NT	**F21**	**3, (6), 7, 8**, (9), **10**	**3,** (5), **6, 7, 8, 10**	NO ROHO		Location indicated above (in green)

Development • Supplementary information

Mid-ventral alar plate trunk NT	**F22**	NO ROHO	NO ROHO	3, 6, 7, 8, 9, 10	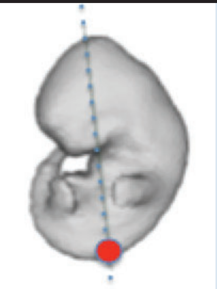	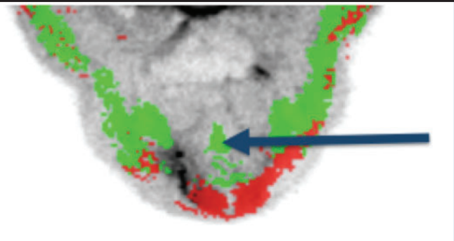

In the Table above, the Wnt and Fzd ROHOs are numbered W1, W2, etc., F1, F2, etc. respectively. The location of each ROHO is illustrated at the latest stage the ROHO is present. **Bold** gene numbers indicate genes expressed in the ROHO at all stages when this ROHO is present. Gene numbers in parenthesis indicate that the expression domain or intersection with the ROHO is small, comprising only a few voxels. Note the number of genes listed can exceed the maximum occupancy for that ROHO because some genes intersect only part of the ROHO.

Development • Supplementary information

Table S4. Integrated Wnt expression patterns: Jaccard similarity indices for all gene expression domains for each stage. The full data is available as a Microsoft Excel file SuppTable3_AllData.xlsx.

Supplementary table 4a: E9.5 (Theiler 15), embryo ID EMA36

NAME	Wnt1	Wnt2	Wnt2b	Wnt3	Wnt3a	Wnt4	Wnt5a	Wnt5b	Wnt6	Wnt7a	Wnt7b	Wnt8b	Wnt9b
Wnt1	1	0.000292	0.01641	0.12098	0.104072	0.039686	0.012792	0.02368	0.024837	0.022408	0.053683	0.005726	0.001363
Wnt2	0.000292	1	0.002707	0.009472	0.000588	0.049788	0.030308	0.002943	0.022243	0.015042	0.013397	0.001087	0.004344
Wnt2b	0.01641	0.002707	1	0.022938	0.023191	0.026378	0.035986	0.044288	0.021496	0.015561	0.019761	0.001575	0.004366
Wnt3	0.12098	0.009472	0.022938	1	0.163961	0.085149	0.018225	0.026435	0.078908	0.031704	0.075516	0.001061	0.011226
Wnt3a	0.104072	0.000588	0.023191	0.163961	1	0.146193	0.040396	0.070042	0.099222	0.076969	0.146899	0.037853	0.025217
Wnt4	0.039686	0.049788	0.026378	0.085149	0.146193	1	0.092639	0.024236	0.090004	0.073733	0.104925	0.011215	0.057103
Wnt5a	0.012792	0.030308	0.035986	0.018225	0.040396	0.092639	1	0.060112	0.084335	0.152687	0.114619	0.000979	0.061876
Wnt5b	0.02368	0.002943	0.044288	0.026435	0.070042	0.024236	0.060112	1	0.020623	0.029848	0.045317	0.038145	0.003157
Wnt6	0.024837	0.022243	0.021496	0.078908	0.099222	0.090004	0.084335	0.020623	1	0.057404	0.077685	0.003064	0.030888
Wnt7a	0.022408	0.015042	0.015561	0.031704	0.076969	0.073733	0.152687	0.029848	0.057404	1	0.229856	0.00623	0.033576
Wnt7b	0.053683	0.013397	0.019761	0.075516	0.146899	0.104925	0.114619	0.045317	0.077685	0.229856	1	0.016501	0.018873
Wnt8b	0.005726	0.001087	0.001575	0.001061	0.037853	0.011215	0.000979	0.038145	0.003064	0.00623	0.016501	1	0.001582
Wnt9b	0.001363	0.004344	0.004366	0.011226	0.025217	0.057103	0.061876	0.003157	0.030888	0.033576	0.018873	0.001582	1
Wnt10a	0.011335	0.001227	0.009812	0.011386	0.018079	0.00945	0.00263	0.012009	0.005964	0.005335	0.009828	0.026009	0.003095
Wnt10b	0.008277	0.052501	0.008438	0.024461	0.044081	0.081135	0.113331	0.009587	0.05867	0.143652	0.072086	0.004561	0.066999
Wnt11	0.00091	0.010701	0.00452	0.013578	0.007424	0.015264	0.02556	0.005594	0.059226	0.018579	0.022398	0.00125	0.012951
Wnt16	0.000882	0.005608	0.002394	0.004792	0.007223	0.007589	0.029014	0.002503	0.031574	0.075534	0.051609	0.001104	0.002693
Fzd1	0.001524	0.003243	0.002371	0.008186	0.008404	0.00878	0.010385	0.003179	0.018977	0.010725	0.011453	0.000761	0.013567
Fzd3	0.062834	0.00231	0.008848	0.068102	0.147621	0.088204	0.10494	0.036264	0.046005	0.251652	0.240044	0.01115	0.018494
Fzd4	0.001251	0.017938	0.0044	0.004357	0.010122	0.019594	0.051865	0.005115	0.015264	0.024784	0.015229	0.001512	0.038309
Fzd6	0.021898	0.006153	0.011036	0.032704	0.060852	0.051614	0.068774	0.027264	0.042519	0.080518	0.066056	0.009617	0.06994
Fzd7	0.005028	0.023599	0.005885	0.021055	0.040771	0.089144	0.115796	0.005441	0.074447	0.209308	0.118586	0.00137	0.049267
Fzd8	0.035362	0.012412	0.020213	0.056125	0.12839	0.099516	0.139507	0.034882	0.09746	0.300161	0.248579	0.012243	0.036627
Fzd9	0.062981	0.013652	0.010706	0.110091	0.134616	0.070619	0.035629	0.025042	0.076423	0.059647	0.08712	0.003157	0.014718
Fzd10	0.067792	0.008432	0.012569	0.109207	0.191777	0.134491	0.055903	0.01466	0.083748	0.067129	0.103153	0.000941	0.011029
Tcf/Lef-GFP	0.051334	0.009737	0.016449	0.079164	0.158866	0.100253	0.076301	0.044515	0.083713	0.206389	0.207961	0.014757	0.007457

NAME	Wnt10a	Wnt10b	Wnt11	Wnt16	Fzd1	Fzd3	Fzd4	Fzd6	Fzd7	Fzd8	Fzd9	Fzd10	Tcf/Lef-GFP
Wnt1	0.011335	0.008277	0.00091	0.000882	0.001524	0.062834	0.001251	0.021898	0.005028	0.035362	0.062981	0.067792	0.051334
Wnt2	0.001227	0.052501	0.010701	0.005608	0.003243	0.00231	0.017938	0.006153	0.023599	0.012412	0.013652	0.008432	0.009737
Wnt2b	0.009812	0.008438	0.00452	0.002394	0.002371	0.008848	0.0044	0.011036	0.005885	0.020213	0.010706	0.012569	0.016449
Wnt3	0.011386	0.024461	0.013578	0.004792	0.008186	0.068102	0.004357	0.032704	0.021055	0.056125	0.110091	0.109207	0.079164
Wnt3a	0.018079	0.044081	0.007424	0.007223	0.008404	0.147621	0.010122	0.060852	0.040771	0.12839	0.134616	0.191777	0.158866
Wnt4	0.00945	0.081135	0.015264	0.007589	0.00878	0.088204	0.019594	0.051614	0.089144	0.099516	0.070619	0.134491	0.100253
Wnt5a	0.00263	0.113331	0.02556	0.029014	0.010385	0.10494	0.051865	0.068774	0.115796	0.139507	0.035629	0.055903	0.076301
Wnt5b	0.012009	0.009587	0.005594	0.002503	0.003179	0.036264	0.005115	0.027264	0.005441	0.034882	0.025042	0.01466	0.044515
Wnt6	0.005964	0.05867	0.059226	0.031574	0.018977	0.046005	0.015264	0.042519	0.074447	0.09746	0.076423	0.083748	0.083713
Wnt7a	0.005335	0.143652	0.018579	0.075534	0.010725	0.251652	0.024784	0.080518	0.209308	0.300161	0.059647	0.067129	0.206389
Wnt7b	0.009828	0.072086	0.022398	0.051609	0.011453	0.240044	0.015229	0.066056	0.118586	0.248579	0.08712	0.103153	0.207961
Wnt8b	0.026009	0.004561	0.00125	0.001104	0.000761	0.01115	0.001512	0.009617	0.00137	0.012243	0.003157	0.000941	0.014757
Wnt9b	0.003095	0.066999	0.012951	0.002693	0.013567	0.018494	0.038309	0.06994	0.049267	0.036627	0.014718	0.011029	0.007457
Wnt10a	1	0.009519	0.001507	0.001304	0.008017	0.00954	0.002112	0.01098	0.00329	0.008587	0.006373	0.005082	0.007757
Wnt10b	0.009519	1	0.013561	0.041459	0.010137	0.108146	0.053956	0.093205	0.165389	0.077787	0.054251	0.023553	0.038032
Wnt11	0.001507	0.013561	1	0.012765	0.013214	0.004693	0.002136	0.007528	0.027676	0.025459	0.00725	0.050029	0.018768
Wnt16	0.001304	0.041459	0.012765	1	0.003536	0.059501	0.011343	0.016064	0.070179	0.02538	0.022459	0.010717	0.090441
Fzd1	0.008017	0.010137	0.013214	0.003536	1	0.006988	0.010826	0.009171	0.012073	0.01252	0.003349	0.012919	0.003458
Fzd3	0.00954	0.108146	0.004693	0.059501	0.006988	1	0.016915	0.072866	0.133171	0.169692	0.100582	0.086464	0.210052
Fzd4	0.002112	0.053956	0.002136	0.011343	0.010826	0.016915	1	0.039325	0.053637	0.024121	0.01653	0.004023	0.004695
Fzd6	0.01098	0.093205	0.007528	0.016064	0.009171	0.072866	0.039325	1	0.070093	0.07369	0.043877	0.028851	0.044701
Fzd7	0.00329	0.165389	0.027676	0.070179	0.012073	0.133171	0.053637	0.070093	1	0.154256	0.048241	0.054443	0.125497
Fzd8	0.008587	0.077787	0.025459	0.02538	0.01252	0.169692	0.024121	0.07369	0.154256	1	0.073077	0.093129	0.19975
Fzd9	0.006373	0.054251	0.00725	0.022459	0.003349	0.100582	0.01653	0.043877	0.048241	0.073077	1	0.074191	0.098592
Fzd10	0.005082	0.023553	0.050029	0.010717	0.012919	0.086464	0.004023	0.028851	0.054443	0.093129	0.074191	1	0.125781
Tcf/Lef-GFP	0.007757	0.038032	0.018768	0.090441	0.003458	0.210052	0.004695	0.044701	0.125497	0.19975	0.098592	0.125781	1

Supplementary table 4b: E10.5 (Theiler 17), embryo ID EMA53

NAME	Wnt1	Wnt2	Wnt2b	Wnt3	Wnt3a	Wnt4	Wnt5a	Wnt5b	Wnt6	Wnt7a	Wnt7b	Wnt8a	Wnt8b
Wnt1	1	0.001277	0.048133	0.05556	0.057992	0.057702	0.01682	0.013172	0.018201	0.001889	0.001915	0.003318	0.000482
Wnt2	0.001277	1	0.0029	0.024976	0.001633	0.021415	0.00323	0.001483	0.052622	0.000589	0.004891	0.022377	0.000287
Wnt2b	0.048133	0.0029	1	0.030353	0.067231	0.048723	0.027648	0.001227	0.00708	0.003806	0.000889	0.017514	0.000013
Wnt3	0.05556	0.024976	0.030353	1	0.083367	0.135623	0.050023	0.002837	0.076564	0.045976	0.061839	0.02124	0.000478
Wnt3a	0.057992	0.001633	0.067231	0.083367	1	0.124487	0.051111	0.001814	0.014986	0.037452	0.011712	0.002876	0.020229
Wnt4	0.057702	0.021415	0.048723	0.135623	0.124487	1	0.039038	0.001211	0.026147	0.035402	0.032964	0.017969	0.000514
Wnt5a	0.01682	0.00323	0.027648	0.050023	0.051111	0.039038	1	0.013565	0.054634	0.068244	0.008096	0.002379	0.000007
Wnt5b	0.013172	0.001483	0.001227	0.002837	0.001814	0.001211	0.013565	1	0.001404	0.001701	0.000442	0.00272	0.000048
Wnt6	0.018201	0.052622	0.00708	0.076564	0.014986	0.026147	0.054634	0.001404	1	0.02037	0.016248	0.021839	0.001085
Wnt7a	0.001889	0.000589	0.003806	0.045976	0.037452	0.035402	0.068244	0.001701	0.02037	1	0.236869	0.003069	0.005203
Wnt7b	0.001915	0.004891	0.000889	0.061839	0.011712	0.032964	0.008096	0.000442	0.016248	0.236869	1	0.020777	0.052469
Wnt8a	0.003318	0.022377	0.017514	0.02124	0.002876	0.017969	0.002379	0.00272	0.021839	0.003069	0.020777	1	0.001424
Wnt8b	0.000482	0.000287	0.000013	0.000478	0.020229	0.000514	0.000007	0.000048	0.001085	0.005203	0.052469	0.001424	1
Wnt9a	0.041788	0.015961	0.034841	0.030423	0.033989	0.035827	0.00998	0.001534	0.02924	0.00189	0.028475	0.127942	0.074764
Wnt9b	0.002355	0.008199	0.00053	0.030775	0.000702	0.007514	0.015383	0.001195	0.022585	0.00616	0.007651	0.008153	0.002108
Wnt10a	0.029282	0.01229	0.040004	0.068284	0.194639	0.078685	0.046614	0.002842	0.035303	0.014539	0.010071	0.017561	0.007409
Wnt10b	0.005499	0.005585	0.003789	0.005353	0.001974	0.00229	0.035721	0.001239	0.017384	0.007252	0.000655	0.001442	0.000294
Wnt11	0.002692	0.080299	0.00139	0.045217	0.002415	0.031374	0.027296	0.000764	0.093777	0.018485	0.032394	0.020914	0.00051
Wnt16	0.002689	0.000867	0.000788	0.008736	0.001507	0.001144	0.00075	0.000012	0.081982	0.000899	0.00247	0.005281	0.000079
Fzd1	0.006331	0.020904	0.000862	0.03128	0.027375	0.013984	0.02634	0.001029	0.034484	0.010473	0.013957	0.001704	0.017809
Fzd3	0.028325	0.011495	0.011006	0.139732	0.082674	0.092095	0.044286	0.002676	0.110971	0.115183	0.112093	0.008561	0.012241
Fzd4	0.021386	0.009567	0.058398	0.026086	0.061685	0.04442	0.019293	0.001853	0.027968	0.00693	0.016884	0.134401	0.001181
Fzd5	0.009223	0.01018	0.009936	0.010613	0.011586	0.038956	0.006838	0.00057	0.005264	0.002139	0.003868	0.048048	0.001218
Fzd6	0.028387	0.025228	0.013013	0.117949	0.093224	0.090438	0.066822	0.002787	0.157539	0.086936	0.049028	0.017543	0.018426
Fzd7	0.019848	0.021969	0.015346	0.098782	0.099642	0.052909	0.076913	0.002372	0.110393	0.144888	0.098585	0.006371	0.016888
Fzd8	0.032896	0.016368	0.014083	0.104759	0.106662	0.087954	0.073162	0.001244	0.149961	0.066974	0.021528	0.007417	0.007995
Fzd9	0.021354	0.008696	0.029723	0.036728	0.119606	0.057062	0.052031	0.007954	0.00738	0.034381	0.010425	0.025589	0.013772
Fzd10	0.027561	0.018711	0.010865	0.132912	0.086771	0.09128	0.064867	0.001358	0.142394	0.100172	0.050225	0.005308	0.008086
Lef1	0.009445	0.029201	0.005649	0.081287	0.03569	0.059397	0.062221	0.002044	0.111677	0.100231	0.087977	0.013645	0.013849
Tcf7	0.007382	0.035706	0.002787	0.072195	0.024907	0.051922	0.048092	0.001444	0.118319	0.106098	0.110832	0.014649	0.010825
Tcf7l1	0.003305	0.021915	0.001705	0.060549	0.015926	0.034056	0.037203	0.002252	0.060712	0.121342	0.116455	0.009741	0.00326
Tcf7l2	0.003828	0.030037	0.001545	0.061878	0.020049	0.029789	0.028283	0.002009	0.028603	0.05796	0.09789	0.008217	0.006245
Sfrp1	0.016244	0.031158	0.009533	0.070698	0.026585	0.026281	0.073226	0.00143	0.103844	0.029548	0.019007	0.01265	0.006239
Sfrp2	0.014309	0.012377	0.007655	0.100266	0.061181	0.054988	0.037226	0.00089	0.113427	0.099886	0.103181	0.004868	0.009195
Sfrp3	0.00467	0.073907	0.003018	0.065234	0.027486	0.020079	0.05597	0.001142	0.093211	0.064485	0.083697	0.010755	0.010468
Sfrp4	0.004607	0.034308	0.003248	0.049356	0.02055	0.018528	0.0184	0.002779	0.044661	0.09753	0.105809	0.037083	0.007122
Tcf/Lef-GFP	0.038614	0.02888	0.01357	0.129179	0.063825	0.127234	0.04216	0.001587	0.114262	0.036039	0.051084	0.018322	0.006126

NAME	Wnt9a	Wnt9b	Wnt10a	Wnt10b	Wnt11	Wnt16	Fzd1	Fzd3	Fzd4	Fzd5	Fzd6	Fzd7	Fzd8
Wnt1	0.041788	0.002355	0.029282	0.005499	0.002692	0.002689	0.006331	0.028325	0.021386	0.009223	0.028387	0.019848	0.032896
Wnt2	0.015961	0.008199	0.01229	0.005585	0.080299	0.000867	0.020904	0.011495	0.009567	0.01018	0.025228	0.021969	0.016368
Wnt2b	0.034841	0.00053	0.040004	0.003789	0.00139	0.000788	0.000862	0.011006	0.058398	0.009936	0.013013	0.015346	0.014083
Wnt3	0.030423	0.030775	0.068284	0.005353	0.045217	0.008736	0.03128	0.139732	0.026086	0.010613	0.117949	0.098782	0.104759
Wnt3a	0.033989	0.000702	0.194639	0.001974	0.002415	0.001507	0.027375	0.082674	0.061685	0.011586	0.093224	0.099642	0.106662
Wnt4	0.035827	0.007514	0.078685	0.00229	0.031374	0.001144	0.013984	0.092095	0.04442	0.038956	0.090438	0.052909	0.087954
Wnt5a	0.00998	0.015383	0.046614	0.035721	0.027296	0.00075	0.02634	0.044286	0.019293	0.006838	0.066822	0.076913	0.073162
Wnt5b	0.001534	0.001195	0.002842	0.001239	0.000764	0.000012	0.001029	0.002676	0.001853	0.00057	0.002787	0.002372	0.001244
Wnt6	0.02924	0.022585	0.035303	0.017384	0.093777	0.081982	0.034484	0.110971	0.027968	0.005264	0.157539	0.110393	0.149961
Wnt7a	0.00189	0.00616	0.014539	0.007252	0.018485	0.000899	0.010473	0.115183	0.00693	0.002139	0.086936	0.144888	0.066974
Wnt7b	0.028475	0.007651	0.010071	0.000655	0.032394	0.00247	0.013957	0.112093	0.016884	0.003868	0.049028	0.098585	0.021528
Wnt8a	0.127942	0.008153	0.017561	0.001442	0.020914	0.005281	0.001704	0.008561	0.134401	0.048048	0.017543	0.006371	0.007417
Wnt8b	0.074764	0.002108	0.007409	0.000294	0.00051	0.000079	0.017809	0.012241	0.001181	0.001218	0.018426	0.016888	0.007995
Wnt9a	1	0.004328	0.035829	0.000963	0.018766	0.004935	0.008573	0.019366	0.09596	0.105737	0.031515	0.017287	0.017918
Wnt9b	0.004328	1	0.007608	0.002121	0.011189	0.000728	0.028568	0.010274	0.011967	0.003116	0.008471	0.009448	0.003241
Wnt10a	0.035829	0.007608	1	0.017597	0.00964	0.002764	0.025825	0.063919	0.08791	0.027727	0.076026	0.092271	0.092279
Wnt10b	0.000963	0.002121	0.017597	1	0.00582	0.000366	0.003097	0.001653	0.005015	0.002613	0.010826	0.00804	0.010844
Wnt11	0.018766	0.011189	0.00964	0.00582	1	0.039775	0.059931	0.095544	0.012577	0.011734	0.121452	0.062698	0.092943
Wnt16	0.004935	0.000728	0.002764	0.000366	0.039775	1	0.060741	0.048268	0.004912	0.001356	0.06432	0.039035	0.093281
Fzd1	0.008573	0.028568	0.025825	0.003097	0.059931	0.060741	1	0.039747	0.0104	0.004529	0.04568	0.043219	0.042157
Fzd3	0.019366	0.010274	0.063919	0.001653	0.095544	0.048268	0.039747	1	0.027288	0.005927	0.348018	0.233855	0.27085
Fzd4	0.09596	0.011967	0.08791	0.005015	0.012577	0.004912	0.0104	0.027288	1	0.013405	0.032358	0.027901	0.027884
Fzd5	0.105737	0.003116	0.027727	0.002613	0.011734	0.001356	0.004529	0.005927	0.013405	1	0.007369	0.006354	0.007136
Fzd6	0.031515	0.008471	0.076026	0.010826	0.121452	0.06432	0.04568	0.348018	0.032358	0.007369	1	0.300946	0.430345
Fzd7	0.017287	0.009448	0.092271	0.00804	0.062698	0.039035	0.043219	0.233855	0.027901	0.006354	0.300946	1	0.295513
Fzd8	0.017918	0.003241	0.092279	0.010844	0.092943	0.093281	0.042157	0.27085	0.027884	0.007136	0.430345	0.295513	1
Fzd9	0.067925	0.002752	0.111021	0.001033	0.007138	0.000056	0.01447	0.039308	0.038697	0.146118	0.042731	0.051449	0.040544
Fzd10	0.015164	0.007468	0.066704	0.006699	0.117972	0.048294	0.02995	0.36238	0.021237	0.005937	0.424354	0.304566	0.386747
Lef1	0.019361	0.009911	0.030665	0.006743	0.148798	0.039864	0.019948	0.226356	0.017761	0.004201	0.253435	0.143093	0.189799
Tcf7	0.015937	0.009195	0.023224	0.005653	0.17179	0.05044	0.023435	0.259503	0.013785	0.005237	0.282379	0.193284	0.233071
Tcf7l1	0.011685	0.008658	0.011061	0.001553	0.164843	0.067426	0.047192	0.222576	0.014547	0.005592	0.180575	0.148449	0.131862
Tcf7l2	0.006196	0.012845	0.024643	0.000944	0.168597	0.007438	0.051077	0.135165	0.007265	0.0124	0.070422	0.058357	0.043582
Sfrp1	0.018318	0.007791	0.040068	0.007603	0.151431	0.005543	0.033585	0.143459	0.027034	0.004158	0.141062	0.11094	0.140351
Sfrp2	0.012917	0.003565	0.05836	0.003753	0.132159	0.054017	0.041341	0.312773	0.022067	0.00579	0.294299	0.219069	0.275736
Sfrp3	0.014828	0.008762	0.021482	0.008668	0.15067	0.009773	0.023677	0.167255	0.021923	0.002819	0.151864	0.122528	0.128091
Sfrp4	0.023998	0.005142	0.014879	0.002439	0.134591	0.0228	0.022664	0.148889	0.015867	0.011132	0.148348	0.139034	0.097898
Tcf/Lef-GFP	0.031519	0.009159	0.069014	0.005733	0.110948	0.040658	0.022788	0.212233	0.031512	0.019085	0.227903	0.127378	0.206969

NAME	Fzd9	Fzd10	Lef1	Tcf7	Tcf7l1	Tcf7l2	Sfrp1	Sfrp2	Sfrp3	Sfrp4	Tcf/Lef-GFP
Wnt1	0.021354	0.027561	0.009445	0.007382	0.003305	0.003828	0.016244	0.014309	0.00467	0.004607	0.038614
Wnt2	0.008696	0.018711	0.029201	0.035706	0.021915	0.030037	0.031158	0.012377	0.073907	0.034308	0.02888
Wnt2b	0.029723	0.010865	0.005649	0.002787	0.001705	0.001545	0.009533	0.007655	0.003018	0.003248	0.01357
Wnt3	0.036728	0.132912	0.081287	0.072195	0.060549	0.061878	0.070698	0.100266	0.065234	0.049356	0.129179
Wnt3a	0.119606	0.086771	0.03569	0.024907	0.015926	0.020049	0.026585	0.061181	0.027486	0.02055	0.063825
Wnt4	0.057062	0.09128	0.059397	0.051922	0.034056	0.029789	0.026281	0.054988	0.020079	0.018528	0.127234
Wnt5a	0.052031	0.064867	0.062221	0.048092	0.037203	0.028283	0.073226	0.037226	0.05597	0.0184	0.04216
Wnt5b	0.007954	0.001358	0.002044	0.001444	0.002252	0.002009	0.00143	0.00089	0.001142	0.002779	0.001587
Wnt6	0.00738	0.142394	0.111677	0.118319	0.060712	0.028603	0.103844	0.113427	0.093211	0.044661	0.114262
Wnt7a	0.034381	0.100172	0.100231	0.106098	0.121342	0.05796	0.029548	0.099886	0.064485	0.09753	0.036039
Wnt7b	0.010425	0.050225	0.087977	0.110832	0.116455	0.09789	0.019007	0.103181	0.083697	0.105809	0.051084
Wnt8a	0.025589	0.005308	0.013645	0.014649	0.009741	0.008217	0.01265	0.004868	0.010755	0.037083	0.018322
Wnt8b	0.013772	0.008086	0.013849	0.010825	0.00326	0.006245	0.006239	0.009195	0.010468	0.007122	0.006126
Wnt9a	0.067925	0.015164	0.019361	0.015937	0.011685	0.006196	0.018318	0.012917	0.014828	0.023998	0.031519
Wnt9b	0.002752	0.007468	0.009911	0.009195	0.008658	0.012845	0.007791	0.003565	0.008762	0.005142	0.009159
Wnt10a	0.111021	0.066704	0.030665	0.023224	0.011061	0.024643	0.040068	0.05836	0.021482	0.014879	0.069014
Wnt10b	0.001033	0.006699	0.006743	0.005653	0.001553	0.000944	0.007603	0.003753	0.008668	0.002439	0.005733
Wnt11	0.007138	0.117972	0.148798	0.17179	0.164843	0.168597	0.151431	0.132159	0.15067	0.134591	0.110948
Wnt16	0.000056	0.048294	0.039864	0.05044	0.067426	0.007438	0.005543	0.054017	0.009773	0.0228	0.040658
Fzd1	0.01447	0.02995	0.019948	0.023435	0.047192	0.051077	0.033585	0.041341	0.023677	0.022664	0.022788
Fzd3	0.039308	0.36238	0.226356	0.259503	0.222576	0.135165	0.143459	0.312773	0.167255	0.148889	0.212233
Fzd4	0.038697	0.021237	0.017761	0.013785	0.014547	0.007265	0.027034	0.022067	0.021923	0.015867	0.031512
Fzd5	0.146118	0.005937	0.004201	0.005237	0.005592	0.0124	0.004158	0.00579	0.002819	0.011132	0.019085
Fzd6	0.042731	0.424354	0.253435	0.282379	0.180575	0.070422	0.141062	0.294299	0.151864	0.148348	0.227903
Fzd7	0.051449	0.304566	0.143093	0.193284	0.148449	0.058357	0.11094	0.219069	0.122528	0.139034	0.127378
Fzd8	0.040544	0.386747	0.189799	0.233071	0.131862	0.043582	0.140351	0.275736	0.128091	0.097898	0.206969
Fzd9	1	0.036447	0.017	0.011147	0.014271	0.022524	0.015138	0.025933	0.009808	0.02117	0.027311
Fzd10	0.036447	1	0.267503	0.318693	0.203086	0.087396	0.186085	0.339677	0.198154	0.16734	0.214248
Lef1	0.017	0.267503	1	0.347459	0.253198	0.118571	0.204368	0.19672	0.20455	0.160809	0.161687
Tcf7	0.011147	0.318693	0.347459	1	0.299636	0.124761	0.206923	0.257505	0.221908	0.214514	0.096471
Tcf7l1	0.014271	0.203086	0.253198	0.299636	1	0.221488	0.179855	0.195584	0.223796	0.262829	0.175777
Tcf7l2	0.022524	0.087396	0.118571	0.124761	0.221488	1	0.138548	0.128493	0.152077	0.153646	0.090695
Sfrp1	0.015138	0.186085	0.204368	0.206923	0.179855	0.138548	1	0.182682	0.307294	0.149561	0.06014
Sfrp2	0.025933	0.339677	0.19672	0.257505	0.195584	0.128493	0.182682	1	0.189741	0.129372	0.173543
Sfrp3	0.009808	0.198154	0.20455	0.221908	0.223796	0.152077	0.307294	0.189741	1	0.181928	0.083097
Sfrp4	0.02117	0.16734	0.160809	0.214514	0.262829	0.153646	0.149561	0.129372	0.181928	1	0.047474
Tcf/Lef-GFP	0.027311	0.214248	0.161687	0.096471	0.175777	0.090695	0.06014	0.173543	0.083097	0.047474	1

Supplementary table 4c: E11. 5 (Theiler 19), embryo ID EMA118

NAME	Wnt1	Wnt2	Wnt2b	Wnt3	Wnt3a	Wnt4	Wnt5a	Wnt5b	Wnt6	Wnt7a	Wnt7b	Wnt8a	Wnt8b
Wnt1	1	0.00537	0.024659	0.119715	0.064444	0.043505	0.022093	0.012047	0.004403	0.02665	0.043973	0.047951	0.011201
Wnt2	0.00537	1	0.047805	0.030677	0.020306	0.056152	0.05464	0.069977	0.023738	0.026059	0.023319	0.041351	0.000554
Wnt2b	0.024659	0.047805	1	0.048754	0.153378	0.115439	0.116082	0.058079	0.04913	0.095484	0.081044	0.209352	0.003366
Wnt3	0.119715	0.030677	0.048754	1	0.109404	0.159231	0.08063	0.049248	0.033445	0.086477	0.157837	0.141085	0.121857
Wnt3a	0.064444	0.020306	0.153378	0.109404	1	0.166796	0.18291	0.058506	0.0589	0.081699	0.144476	0.231141	0.028768
Wnt4	0.043505	0.056152	0.115439	0.159231	0.166796	1	0.120258	0.072765	0.020101	0.234245	0.304887	0.185272	0.086626
Wnt5a	0.022093	0.05464	0.116082	0.08063	0.18291	0.120258	1	0.091121	0.053647	0.111942	0.131332	0.206168	0.033288
Wnt5b	0.012047	0.069977	0.058079	0.049248	0.058506	0.072765	0.091121	1	0.014637	0.056257	0.057895	0.063715	0.021529
Wnt6	0.004403	0.023738	0.04913	0.033445	0.0589	0.020101	0.053647	0.014637	1	0.01644	0.033771	0.04165	0.00225
Wnt7a	0.02665	0.026059	0.095484	0.086477	0.081699	0.234245	0.111942	0.056257	0.01644	1	0.35565	0.122231	0.040471
Wnt7b	0.043973	0.023319	0.081044	0.157837	0.144476	0.304887	0.131332	0.057895	0.033771	0.35565	1	0.163768	0.084419
Wnt8a	0.047951	0.041351	0.209352	0.141085	0.231141	0.185272	0.206168	0.063715	0.04165	0.122231	0.163768	1	0.048266
Wnt8b	0.011201	0.000554	0.003366	0.121857	0.028768	0.086626	0.033288	0.021529	0.00225	0.040471	0.084419	0.048266	1
Wnt9a	0.018732	0.070175	0.052101	0.088864	0.026375	0.121623	0.082489	0.065142	0.008652	0.112641	0.10184	0.107948	0.081914
Wnt9b	0.009429	0.029912	0.052011	0.074494	0.023086	0.075331	0.091645	0.057934	0.029664	0.094748	0.095311	0.074115	0.051418
Wnt10a	0.048273	0.0106	0.02699	0.061305	0.059479	0.039532	0.008232	0.032928	0.009023	0.016786	0.02861	0.044679	0.042249
Wnt10b	0.023285	0.057389	0.029844	0.049132	0.037372	0.035657	0.02072	0.051136	0.040221	0.016148	0.018614	0.033149	0.003972
Wnt11	0.004972	0.052078	0.077388	0.084896	0.04978	0.079437	0.116526	0.040689	0.039053	0.060611	0.059933	0.105831	0.072527
Wnt16	0.013883	0.150735	0.055614	0.117095	0.075557	0.210153	0.142565	0.09562	0.009663	0.133633	0.148307	0.113476	0.076817
Fzd1	0.0319	0.028329	0.167778	0.088216	0.221429	0.138271	0.166305	0.068796	0.038992	0.071312	0.101471	0.271576	0.039573
Fzd3	0.033941	0.034588	0.15458	0.127614	0.165174	0.281742	0.215439	0.090925	0.026015	0.357024	0.355855	0.213947	0.049726
Fzd4	0.016651	0.124945	0.092831	0.029947	0.044238	0.051849	0.094022	0.075283	0.025514	0.037957	0.027789	0.08424	0.001909
Fzd5	0.0167	0.040673	0.021809	0.135098	0.026416	0.126795	0.065308	0.04494	0.013037	0.0782	0.102226	0.081746	0.156621
Fzd6	0.019758	0.077413	0.196323	0.08069	0.164657	0.219105	0.227536	0.08326	0.031419	0.234589	0.212031	0.190719	0.034471
Fzd7	0.01215	0.057684	0.146259	0.035671	0.110546	0.115117	0.192525	0.055613	0.045517	0.106124	0.105356	0.127352	0.019327
Fzd8	0.016992	0.047116	0.135584	0.081799	0.120985	0.19127	0.190074	0.09388	0.015909	0.164861	0.153807	0.168834	0.053601
Fzd9	0.038374	0.050286	0.089998	0.129809	0.094476	0.171511	0.100006	0.042842	0.019587	0.097589	0.141123	0.154882	0.130236
Fzd10	0.069017	0.036883	0.147139	0.13608	0.289397	0.196965	0.251985	0.055825	0.065559	0.120395	0.211717	0.283793	0.021099
Lef1	0.028071	0.060399	0.125984	0.092618	0.212149	0.218976	0.270728	0.098888	0.043077	0.159917	0.212104	0.213712	0.038021
Tcf7	0.016219	0.047169	0.124165	0.066345	0.168927	0.167069	0.293312	0.094313	0.036822	0.183634	0.199007	0.186314	0.033099
Tcf7l1	0.013551	0.026109	0.129313	0.058755	0.167765	0.162814	0.253672	0.086124	0.017961	0.198228	0.169554	0.180882	0.016035
Tcf7l2	0.009398	0.015088	0.009773	0.037959	0.026125	0.038678	0.050077	0.047076	0.004039	0.019951	0.03829	0.040931	0.009222
Sfrp1	0.037629	0.073289	0.159048	0.108698	0.228545	0.269069	0.22811	0.063517	0.033923	0.205101	0.261234	0.266297	0.043873
Sfrp2	0.032579	0.077636	0.078694	0.076919	0.157248	0.112424	0.17441	0.073608	0.022758	0.096299	0.108845	0.175583	0.014142
Sfrp3	0.020536	0.079737	0.079485	0.064695	0.130391	0.105267	0.189083	0.096453	0.034243	0.08558	0.112675	0.16282	0.011209
Sfrp4	0.023586	0.08309	0.160887	0.091513	0.142441	0.189735	0.229629	0.095257	0.032317	0.182075	0.193993	0.179096	0.034195
Tcf/Lef-GFP	0.031246	0.078235	0.220417	0.077919	0.245902	0.209716	0.150268	0.05342	0.052563	0.147404	0.182203	0.198223	0.012026

NAME	Wnt9a	Wnt9b	Wnt10a	Wnt10b	Wnt11	Wnt16	Fzd1	Fzd3	Fzd4	Fzd5	Fzd6	Fzd7	Fzd8
Wnt1	0.018732	0.009429	0.048273	0.023285	0.004972	0.013883	0.0319	0.033941	0.016651	0.0167	0.019758	0.01215	0.016992
Wnt2	0.070175	0.029912	0.0106	0.057389	0.052078	0.150735	0.028329	0.034588	0.124945	0.040673	0.077413	0.057684	0.047116
Wnt2b	0.052101	0.052011	0.02699	0.029844	0.077388	0.055614	0.167778	0.15458	0.092831	0.021809	0.196323	0.146259	0.135584
Wnt3	0.088864	0.074494	0.061305	0.049132	0.084896	0.117095	0.088216	0.127614	0.029947	0.135098	0.08069	0.035671	0.081799
Wnt3a	0.026375	0.023086	0.059479	0.037372	0.04978	0.075557	0.221429	0.165174	0.044238	0.026416	0.164657	0.110546	0.120985
Wnt4	0.121623	0.075331	0.039532	0.035657	0.079437	0.210153	0.138271	0.281742	0.051849	0.126795	0.219105	0.115117	0.19127
Wnt5a	0.082489	0.091645	0.008232	0.02072	0.116526	0.142565	0.166305	0.215439	0.094022	0.065308	0.227536	0.192525	0.190074
Wnt5b	0.065142	0.057934	0.032928	0.051136	0.040689	0.09562	0.068796	0.090925	0.075283	0.04494	0.08326	0.055613	0.09388
Wnt6	0.008652	0.029664	0.009023	0.040221	0.039053	0.009663	0.038992	0.026015	0.025514	0.013037	0.031419	0.045517	0.015909
Wnt7a	0.112641	0.094748	0.016786	0.016148	0.060611	0.133633	0.071312	0.357024	0.037957	0.0782	0.234589	0.106124	0.164861
Wnt7b	0.10184	0.095311	0.02861	0.018614	0.059933	0.148307	0.101471	0.355855	0.027789	0.102226	0.212031	0.105356	0.153807
Wnt8a	0.107948	0.074115	0.044679	0.033149	0.105831	0.113476	0.271576	0.213947	0.08424	0.081746	0.190719	0.127352	0.168834
Wnt8b	0.081914	0.051418	0.042249	0.003972	0.072527	0.076817	0.039573	0.049726	0.001909	0.156621	0.034471	0.019327	0.053601
Wnt9a	1	0.123735	0.00554	0.016904	0.103782	0.193132	0.051494	0.10476	0.091511	0.154717	0.120455	0.071736	0.125082
Wnt9b	0.123735	1	0.001637	0.01167	0.074415	0.114474	0.054737	0.109226	0.05706	0.101332	0.08926	0.092599	0.081272
Wnt10a	0.00554	0.001637	1	0.062097	0.004094	0.020924	0.059971	0.027389	0.014562	0.015452	0.020251	0.010343	0.029346
Wnt10b	0.016904	0.01167	0.062097	1	0.022583	0.053811	0.038236	0.019656	0.070808	0.024378	0.02268	0.013001	0.015296
Wnt11	0.103782	0.074415	0.004094	0.022583	1	0.092468	0.061922	0.065069	0.059969	0.121578	0.08585	0.074589	0.099428
Wnt16	0.193132	0.114474	0.020924	0.053811	0.092468	1	0.100214	0.17961	0.124962	0.149197	0.203632	0.100014	0.227001
Fzd1	0.051494	0.054737	0.059971	0.038236	0.061922	0.100214	1	0.174847	0.072348	0.040241	0.17566	0.181922	0.210003
Fzd3	0.10476	0.109226	0.027389	0.019656	0.065069	0.17961	0.174847	1	0.058493	0.078158	0.389291	0.195995	0.291621
Fzd4	0.091511	0.05706	0.014562	0.070808	0.059969	0.124962	0.072348	0.058493	1	0.04079	0.058216	0.08133	0.084906
Fzd5	0.154717	0.101332	0.015452	0.024378	0.121578	0.149197	0.040241	0.078158	0.04079	1	0.066762	0.041222	0.082899
Fzd6	0.120455	0.08926	0.020251	0.02268	0.08585	0.203632	0.17566	0.389291	0.058216	0.066762	1	0.198977	0.33404
Fzd7	0.071736	0.092599	0.010343	0.013001	0.074589	0.100014	0.181922	0.195995	0.08133	0.041222	0.198977	1	0.217198
Fzd8	0.125082	0.081272	0.029346	0.015296	0.099428	0.227001	0.210003	0.291621	0.084906	0.082899	0.33404	0.217198	1
Fzd9	0.110137	0.058997	0.035578	0.01646	0.078773	0.144668	0.154791	0.132038	0.05929	0.114766	0.121154	0.105804	0.143487
Fzd10	0.028178	0.045849	0.039476	0.042319	0.055314	0.068024	0.20876	0.240122	0.054473	0.035582	0.173436	0.143074	0.120025
Lef1	0.056076	0.067304	0.028984	0.025747	0.078973	0.125859	0.204506	0.30155	0.069231	0.048209	0.279514	0.189242	0.230823
Tcf7	0.099381	0.09087	0.015564	0.020513	0.082729	0.159781	0.154502	0.310665	0.062429	0.059082	0.352839	0.16881	0.239522
Tcf7l1	0.056927	0.068112	0.023787	0.016473	0.081823	0.135603	0.222847	0.278042	0.05233	0.036895	0.291446	0.234421	0.328346
Tcf7l2	0.020963	0.029799	0.008242	0.003046	0.05192	0.036428	0.042293	0.031498	0.015304	0.032559	0.032764	0.014887	0.050111
Sfrp1	0.098571	0.072478	0.032507	0.021211	0.072461	0.165945	0.232187	0.392399	0.050186	0.066408	0.371028	0.207792	0.319142
Sfrp2	0.043966	0.04351	0.055976	0.071923	0.073787	0.10679	0.187458	0.156737	0.104192	0.03301	0.140731	0.1235	0.159032
Sfrp3	0.04092	0.067772	0.036261	0.071419	0.055381	0.107639	0.150555	0.146805	0.139199	0.030907	0.114797	0.100068	0.103381
Sfrp4	0.135593	0.100361	0.021766	0.018959	0.079944	0.199364	0.180042	0.352793	0.089009	0.070511	0.39621	0.200187	0.337458
Tcf/Lef-GFP	0.040599	0.038505	0.031185	0.029781	0.067329	0.080825	0.162096	0.057242	0.025799	0.252304	0.146811	0.153245	0.097649

NAME	Fzd9	Fzd10	Lef1	Tcf7	Tcf7l1	Tcf7l2	Sfrp1	Sfrp2	Sfrp3	Sfrp4	Tcf/Lef-GFP
Wnt1	0.038374	0.069017	0.028071	0.016219	0.013551	0.009398	0.037629	0.032579	0.020536	0.023586	0.031246
Wnt2	0.050286	0.036883	0.060399	0.047169	0.026109	0.015088	0.073289	0.077636	0.079737	0.08309	0.078235
Wnt2b	0.089998	0.147139	0.125984	0.124165	0.129313	0.009773	0.159048	0.078694	0.079485	0.160887	0.220417
Wnt3	0.129809	0.13608	0.092618	0.066345	0.058755	0.037959	0.108698	0.076919	0.064695	0.091513	0.077919
Wnt3a	0.094476	0.289397	0.212149	0.168927	0.167765	0.026125	0.228545	0.157248	0.130391	0.142441	0.245902
Wnt4	0.171511	0.196965	0.218976	0.167069	0.162814	0.038678	0.269069	0.112424	0.105267	0.189735	0.209716
Wnt5a	0.100006	0.251985	0.270728	0.293312	0.253672	0.050077	0.22811	0.17441	0.189083	0.229629	0.150268
Wnt5b	0.042842	0.055825	0.098888	0.094313	0.086124	0.047076	0.063517	0.073608	0.096453	0.095257	0.05342
Wnt6	0.019587	0.065559	0.043077	0.036822	0.017961	0.004039	0.033923	0.022758	0.034243	0.032317	0.052563
Wnt7a	0.097589	0.120395	0.159917	0.183634	0.198228	0.019951	0.205101	0.096299	0.08558	0.182075	0.147404
Wnt7b	0.141123	0.211717	0.212104	0.199007	0.169554	0.03829	0.261234	0.108845	0.112675	0.193993	0.182203
Wnt8a	0.154882	0.283793	0.213712	0.186314	0.180882	0.040931	0.266297	0.175583	0.16282	0.179096	0.198223
Wnt8b	0.130236	0.021099	0.038021	0.033099	0.016035	0.009222	0.043873	0.014142	0.011209	0.034195	0.012026
Wnt9a	0.110137	0.028178	0.056076	0.099381	0.056927	0.020963	0.098571	0.043966	0.04092	0.135593	0.040599
Wnt9b	0.058997	0.045849	0.067304	0.09087	0.068112	0.029799	0.072478	0.04351	0.067772	0.100361	0.038505
Wnt10a	0.035578	0.039476	0.028984	0.015564	0.023787	0.008242	0.032507	0.055976	0.036261	0.021766	0.031185
Wnt10b	0.01646	0.042319	0.025747	0.020513	0.016473	0.003046	0.021211	0.071923	0.071419	0.018959	0.029781
Wnt11	0.078773	0.055314	0.078973	0.082729	0.081823	0.05192	0.072461	0.073787	0.055381	0.079944	0.067329
Wnt16	0.144668	0.068024	0.125859	0.159781	0.135603	0.036428	0.165945	0.10679	0.107639	0.199364	0.080825
Fzd1	0.154791	0.20876	0.204506	0.154502	0.222847	0.042293	0.232187	0.187458	0.150555	0.180042	0.162096
Fzd3	0.132038	0.240122	0.30155	0.310665	0.278042	0.031498	0.392399	0.156737	0.146805	0.352793	0.057242
Fzd4	0.05929	0.054473	0.069231	0.062429	0.05233	0.015304	0.050186	0.104192	0.139199	0.089009	0.025799
Fzd5	0.114766	0.035582	0.048209	0.059082	0.036895	0.032559	0.066408	0.03301	0.030907	0.070511	0.252304
Fzd6	0.121154	0.173436	0.279514	0.352839	0.291446	0.032764	0.371028	0.140731	0.114797	0.39621	0.146811
Fzd7	0.105804	0.143074	0.189242	0.16881	0.234421	0.014887	0.207792	0.1235	0.100068	0.200187	0.153245
Fzd8	0.143487	0.120025	0.230823	0.239522	0.328346	0.050111	0.319142	0.159032	0.103381	0.337458	0.097649
Fzd9	1	0.110467	0.132741	0.099892	0.098939	0.023461	0.14227	0.06964	0.067138	0.124087	0.262672
Fzd10	0.110467	1	0.284698	0.211102	0.192114	0.024284	0.290864	0.191132	0.173	0.172293	0.240747
Lef1	0.132741	0.284698	1	0.363785	0.275602	0.048891	0.318104	0.193365	0.205552	0.278893	0.245255
Tcf7	0.099892	0.211102	0.363785	1	0.283649	0.042487	0.298021	0.158331	0.183906	0.324668	0.304514
Tcf7l1	0.098939	0.192114	0.275602	0.283649	1	0.062675	0.279651	0.200125	0.15417	0.236497	0.142274
Tcf7l2	0.023461	0.024284	0.048891	0.042487	0.062675	1	0.033653	0.070635	0.059435	0.029332	0.110801
Sfrp1	0.14227	0.290864	0.318104	0.298021	0.279651	0.033653	1	0.193909	0.133177	0.330756	0.199996
Sfrp2	0.06964	0.191132	0.193365	0.158331	0.200125	0.070635	0.193909	1	0.225405	0.143433	0.206779
Sfrp3	0.067138	0.173	0.205552	0.183906	0.15417	0.059435	0.133177	0.225405	1	0.140759	0.177506
Sfrp4	0.124087	0.172293	0.278893	0.324668	0.236497	0.029332	0.330756	0.143433	0.140759	1	0.015043
Tcf/Lef-GFP	0.262672	0.240747	0.245255	0.304514	0.142274	0.110801	0.199996	0.206779	0.177506	0.015043	1

Table S5. details of gene expression probes and EMAGE entry IDs

*Note no specific expression detected for Fzd2 and Sfrp5 at these stages

Gene	Extent of Probe on Genbank Sequence	Source	EMAGE IDs
Wnt1	Nucleotide 138 to 2345 on genbank sequence BC005449.1	A. McMahon	EMAGE:6131<br>EMAGE:6132<br>EMAGE:3949
Wnt2	Nucleotide 19 to 1493 on genbank sequence BC026373	A. McMahon	EMAGE:6133<br>EMAGE:6134<br>EMAGE:3950
Wnt2b	Nucleotide 882 to 1103 on genbank sequence AF070988	L. Zakin	EMAGE:6135<br>EMAGE:6136<br>EMAGE:3951
Wnt3	Nucleotide 1610 to 2973 on genbank sequence NM_009521.1	P. Salinas	EMAGE:6137<br>EMAGE:6138<br>EMAGE:3952
Wnt3a	Nucleotide 2310 to 2676 on genbank sequence NM_009522.1	RT-PCR generated	EMAGE:6139<br>EMAGE:6141<br>EMAGE:3953
Wnt4	Nucleotide 853 to 1315 on genbank sequence AK012727	A. McMahon	EMAGE:6140<br>EMAGE:6142<br>EMAGE:3954
Wnt5a	Nucleotide 193 to 2324 on genbank sequence BC018425	A. McMahon	EMAGE:6143<br>EMAGE:6144<br>EMAGE:3955
Wnt5b	Nucleotide 111 to 1429 on genbank sequence BC010775	A. McMahon	EMAGE:6145<br>EMAGE:6146<br>EMAGE:3956
Wnt6	Nucleotide 27 to 2066 on genbank sequence NM_009526.3	A. McMahon	EMAGE:6147<br>EMAGE:6148<br>EMAGE:3957
Wnt7a	Nucleotide 24 to 3164 on genbank sequence BC049093	A. McMahon	EMAGE:6149<br>EMAGE:6150<br>EMAGE:3958
Wnt7b	Nucleotide 93 to 1581 on genbank sequence NM_009528.2	A. McMahon	EMAGE:6151<br>EMAGE:6152<br>EMAGE:3959
Wnt8a	Nucleotide 87 to 1746 on genbank sequence NM_009290.1	P. Chambon	EMAGE:6153<br>EMAGE:3960
Wnt8b	Nucleotide 942 to 1631 on genbank sequence NM_011720.2	J. Mason	EMAGE:6166<br>EMAGE:6154<br>EMAGE:3961
Wnt9a	Nucleotide 1698 to 2247 on genbank sequence NM_139298.2	RT-PCR generated	EMAGE:6155<br>EMAGE:6156<br>EMAGE:3962
Wnt9b	Nucleotide 2834 to 3436 on genbank sequence NM_011719.3	RT-PCR generated	EMAGE:6158<br>EMAGE:6157<br>EMAGE:3963
Wnt10a	Nucleotide 289 to 2469 on genbank sequence NM_009518.1	IMAGE clone	EMAGE:6159<br>EMAGE:6160<br>EMAGE:3964
Wnt10b	Nucleotide 27 to 473 on genbank sequence U61970	A. McMahon	EMAGE:6167<br>EMAGE:6161<br>EMAGE:3965
Wnt11	Nucleotide 169 to 1789 on genbank sequence NM_009519.1 + 419bp of 3' UTR	A. McMahon	EMAGE:6162<br>EMAGE:6163<br>EMAGE:3966
Wnt16	Nucleotide 538 to 1532 on genbank sequence NM_053116	PCR cloned	EMAGE:6164<br>EMAGE:6165<br>EMAGE:3967
Fzd1	Nucleotide 2774 to 3663 on genbank sequence NM_021457.2	PCR cloned	EMAGE:6129<br>EMAGE:6130<br>EMAGE:3939
Fzd2*	Nucleotide 3106 to 3441 on genbank sequence BC049774	IMAGE clone	Not detected
Fzd3	Nucleotide 127 o 1485 on genbank sequence BC050965	U. Borello/C. Cossu	EMAGE:6101<br>EMAGE:6115<br>EMAGE:3941
Fzd4	Nucleotide 1419 to 1862 on genbank sequence BC015256	U. Borello/C. Cossu	EMAGE:6116<br>EMAGE:6117<br>EMAGE:3942
Fzd5	Nucleotide 640 to 868 on genbank sequence AB052910	U. Borello/C. Cossu	EMAGE:6118<br>EMAGE:6119<br>EMAGE:3943
Fzd6	Nucleotide 1794 to 2642 on genbank sequence NM_008056.2	U. Borello/C. Cossu	EMAGE:6120<br>EMAGE:6121<br>EMAGE:3944
Fzd7	Nucleotide 191 to 1013 on genbank sequence BC049781	U. Borello/C. Cossu	EMAGE:6122<br>EMAGE:6123<br>EMAGE:3945
Fzd8	Nucleotide 968 to 1965 on genbank sequence NM_008058.1	U. Borello/C. Cossu	EMAGE:6124<br>EMAGE:6125<br>EMAGE:3946
Fzd9	Nucleotide 678 to 1178 on genbank sequence AF033585	U. Borello/C. Cossu	EMAGE:6126<br>EMAGE:3947
Fzd10	Nucleotide 1892 to 2988 on genbank sequence NM_175284.3	IMAGE library	EMAGE:6127<br>EMAGE:6128<br>EMAGE:3948
Lef1	Nucleotide 371 on NM_010703.3	J. Meeldijk	EMAGE:5753 (TS16)<br>EMAGE:5757<br>EMAGE:5758
Tcf7	Nucleotide 18 to 1572 on NM_009331.3	R.Grosschedl	EMAGE:5765<br>EMAGE:5759<br>EMAGE:5760
Tcf7l1	Nucleotide 86 to 1582 on NM_009332.2	Cloned: library screen	EMAGE:5766<br>EMAGE:5761<br>EMAGE:5762
Tcf7l2	Nucleotide 946 to 1361 on NM_009333.3	J.Rubenstein	EMAGE:5754 (TS16)<br>EMAGE:5763<br>EMAGE:5764
β-catenin	Nucleotide 2194 to 2621 on NM_007614.2	U.Borello/ G. Cossu	EMAGE 5752<br>EMAGE 5755<br>EMAGE 5756
Sfrp1	Nucleotide 12 to 784 on NM_013834	A Rattner	Not on EMAGE
Sfrp2	Nucleotide 82 to 852 on U88569	A Rattner	"
Sfrp3	Nucleotide 2059-2522 on NM_011356.4	PCR cloned	"
Sfrp4	Nucleotide 9 to 1756 on NM_016687.3	A Rattner	"
Sfrp5*	Nucleotide 1 to 1081 on NM_018780.3	PCR cloned	Not detected
Wif1	Nucleotide 876 to 2237 on NM_011915.2	IMAGE library	Not on EMAGE
Wise	Nucleotide 81 to 1038 on NM_025312.3	IMAGE library	"
Ror2	Nucleotide 1 to 3968 on NM_013846.3	IMAGE library	"

Development • Supplementary information

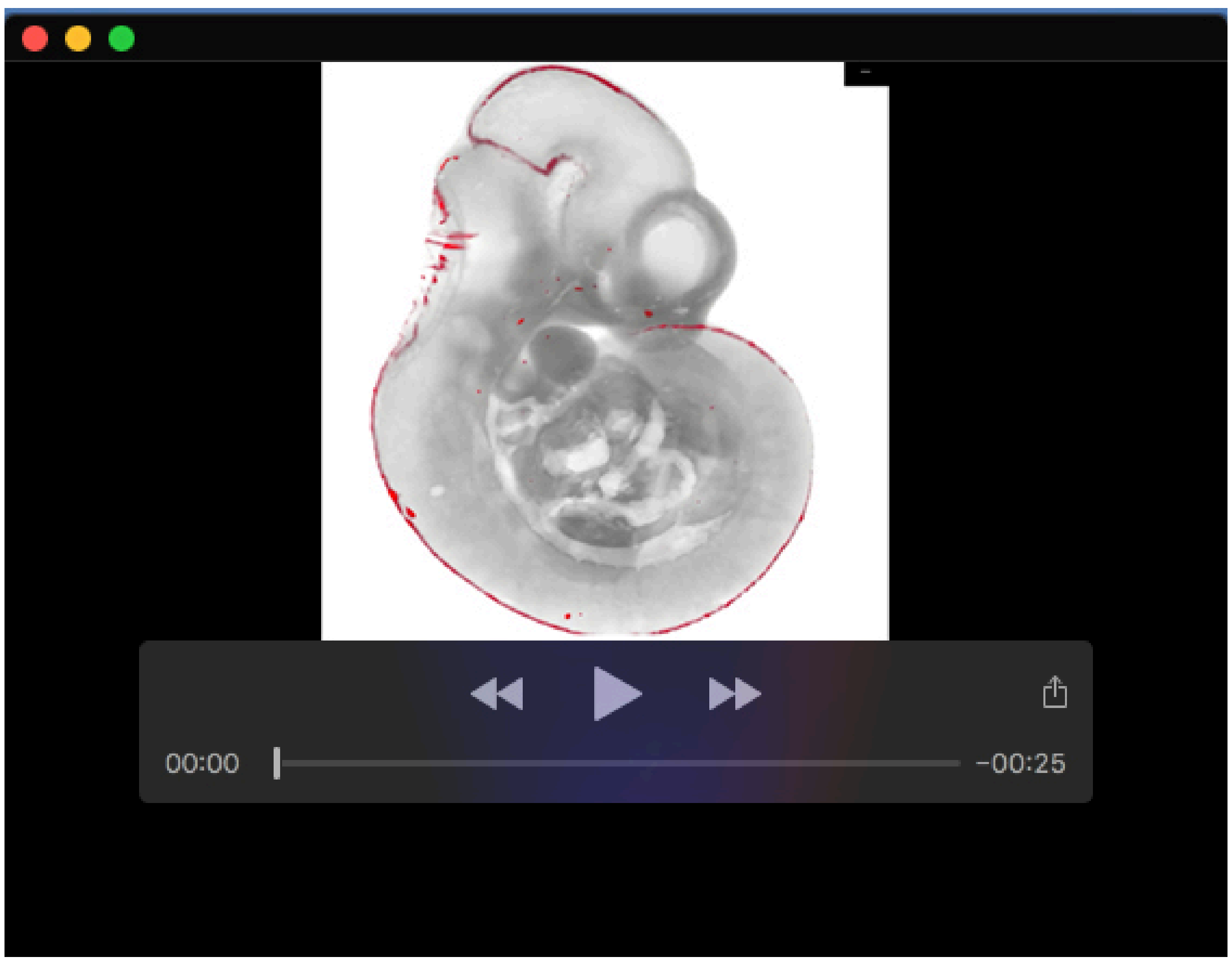

Movie 1. 3D movie of Wnt1 expression pattern at E10.5 mapped onto reference embryo

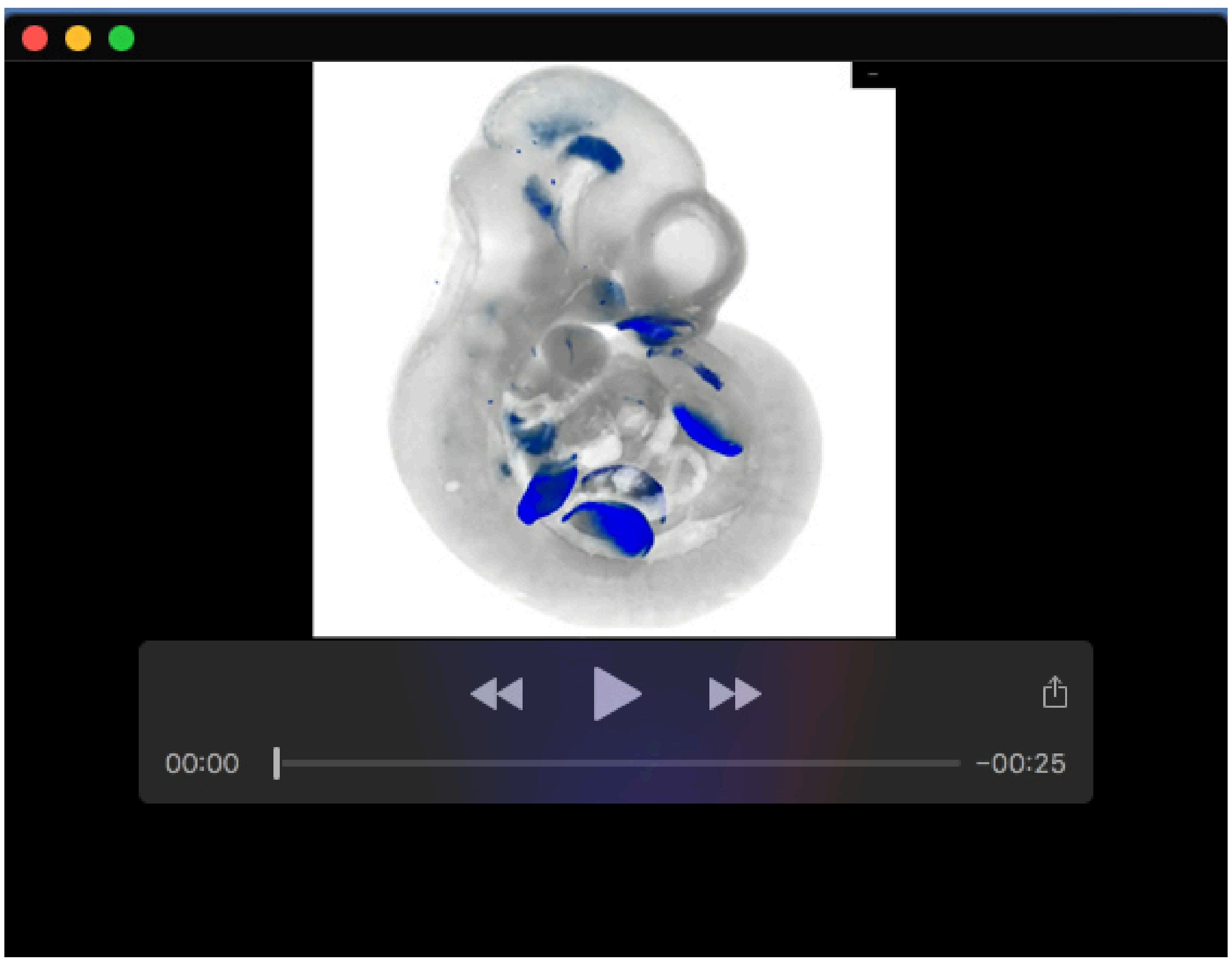

Movie 2. 3D movie of Wnt5a expression pattern at E10.5 mapped onto reference embryo

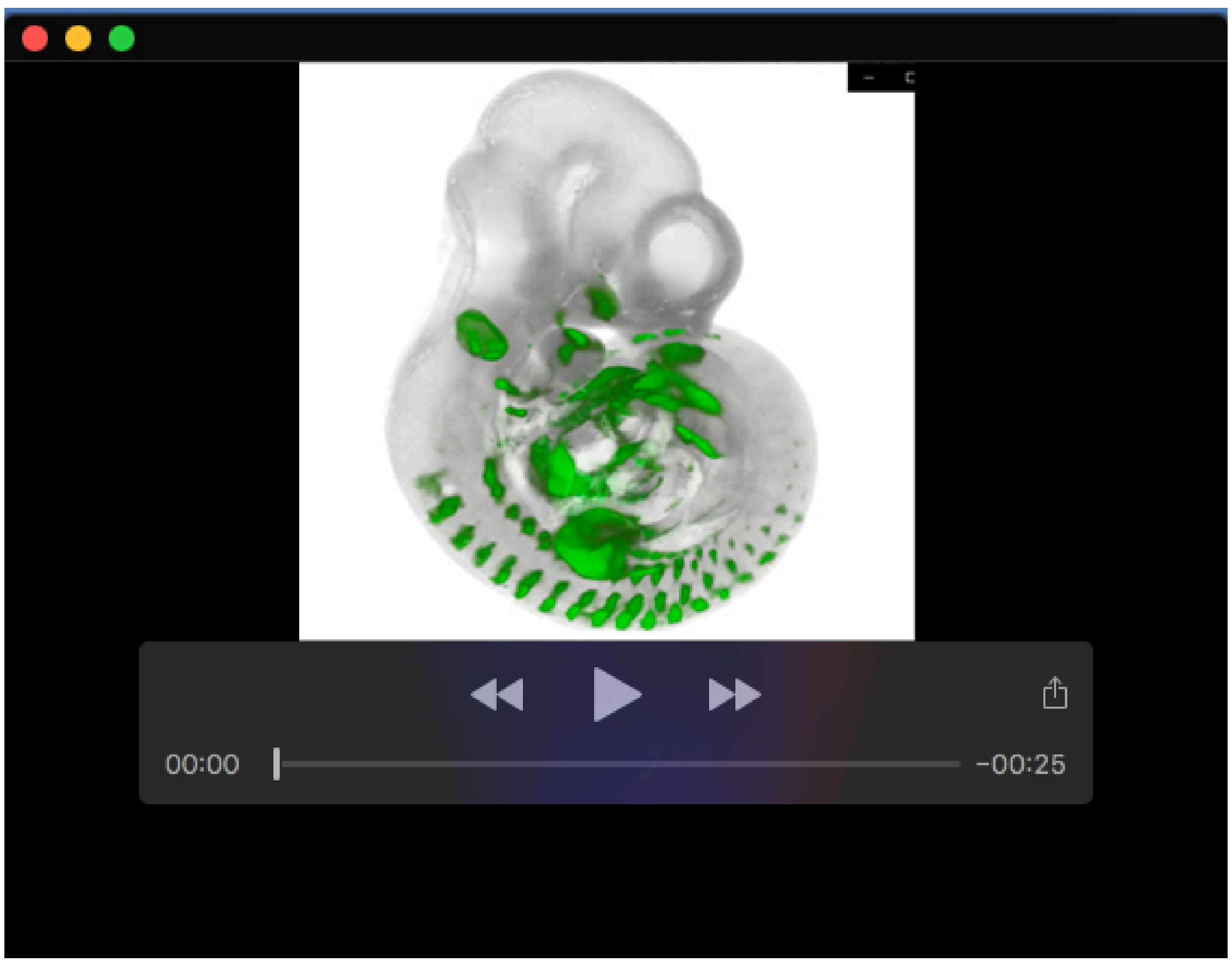

Movie 3. 3D movie of Wnt11 expression pattern at E10.5 mapped onto reference embryo

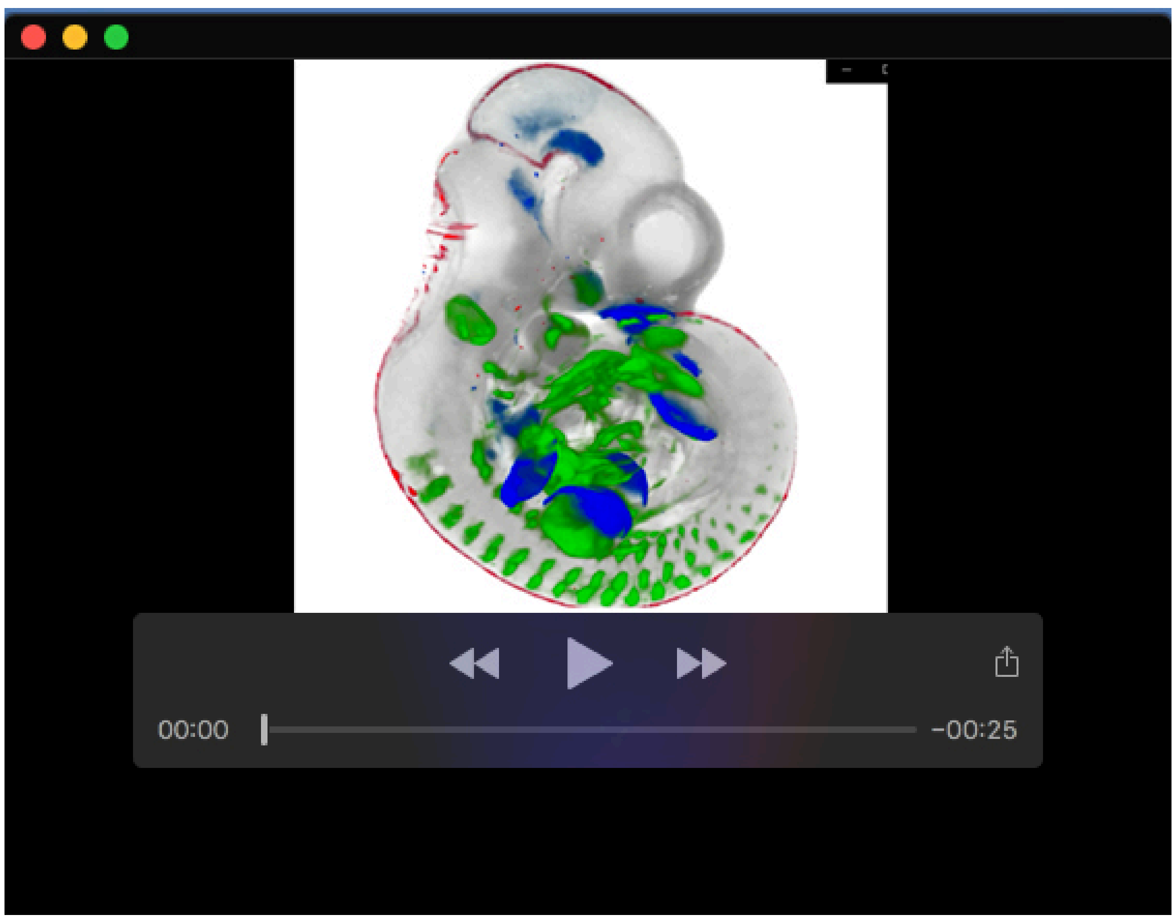

Movie 4. 3D movie of integrated Wnt1 (red), Wnt5a (blue) and Wnt11 (green) expression patterns at E10.5 mapped onto reference embryo

Development • Supplementary information

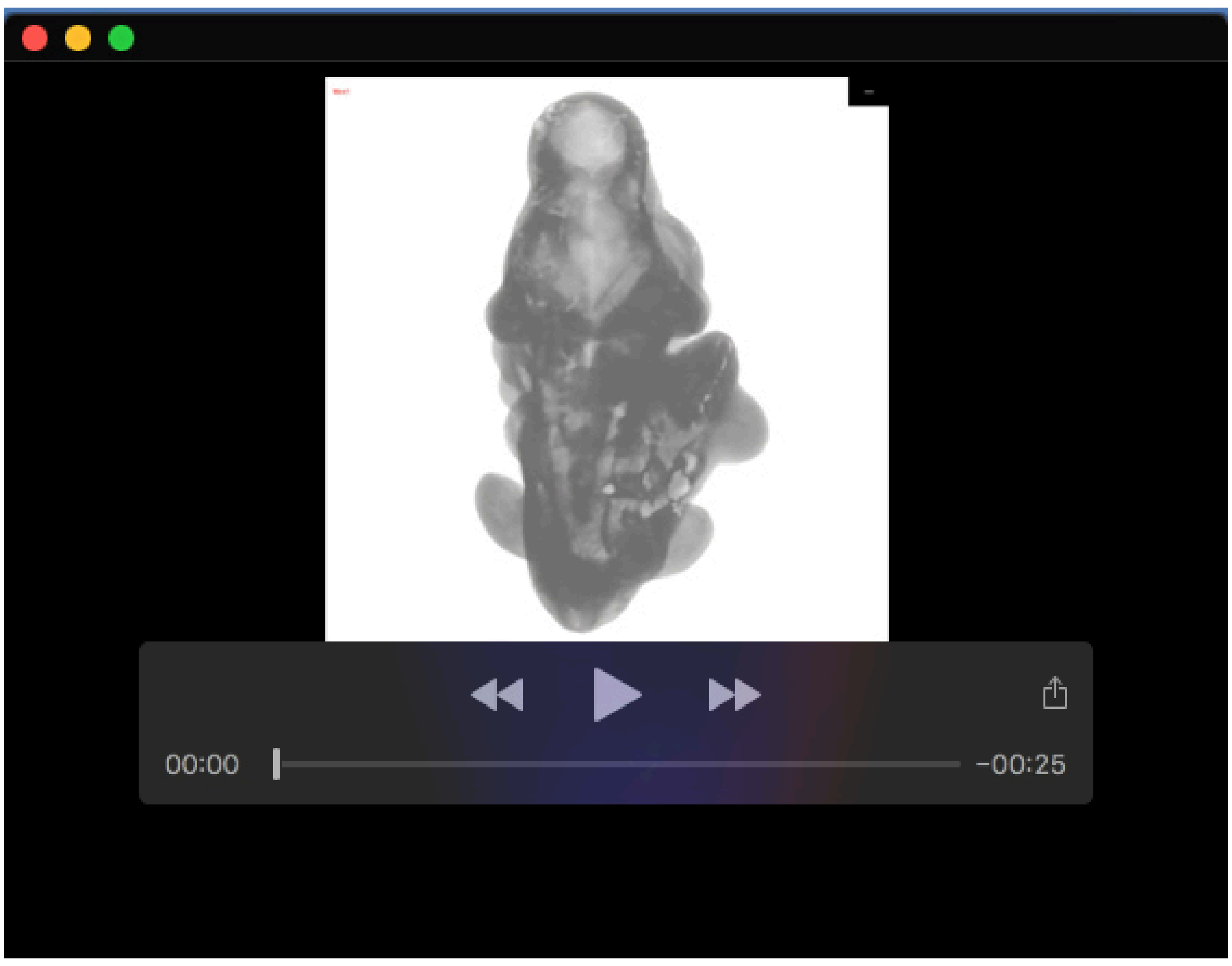

Movie 5. 3D movie of integrated expression patterns of all 19 Wnt genes (colour codes indicated on movie) at E10.5 mapped onto refence embryo.

Development • Supplementary information
