# Supplementary material for: Integrated analysis of Wnt signalling system component gene expression
Source: Development. 2022 Aug 15;149(16):dev200312. doi: 10.1242/dev.200312 (PMC9481969; doi:10.1242/dev.200312)
Supplement: Reviewer comments [file develop-149-200312_review_history.pdf]

Integrated analysis of Wnt signalling system component gene expression

Paula Murphy, Chris Armit, Bill Hill, Shanmugasundaram Venkataraman, Patrick Frankel, Richard A Baldock and Duncan R Davidson
DOI: 10.1242/dev.200312

Editor: Paul François

Review timeline
Original submission: 10 March 2022
Editorial decision: 16 May 2022
First revision received: 15 June 2022
Accepted: 6 July 2022

Original submission

First decision letter

MS ID#: DEVELOP/2021/200312

MS TITLE: Integrated analysis of Wnt signalling system component gene expression

AUTHORS: Paula Murphy, Chris Armit, Bill Hill, Shanmugasundaram Venkataraman, Patrick Frankel, Richard A Baldock, and Duncan R Davidson

I have now received all the referees reports on the above manuscript, and have reached a decision. The referees' comments are appended below, or you can access them online: please go to BenchPress and click on the 'Manuscripts with Decisions' queue in the Author Area.

The overall evaluation is positive and we would like to publish a revised manuscript in Development, provided that the referees' comments can be satisfactorily addressed. Please attend to all of the reviewers' comments in your revised manuscript and detail them in your point-by-point response. If you do not agree with any of their criticisms or suggestions explain clearly why this is so. If it would be helpful, you are welcome to contact us to discuss your revision in greater detail. Please send us a point-by-point response indicating your plans for addressing the referee's comments, and we will look over this and provide further guidance.

Reviewer 1

Advance summary and potential significance to field

This manuscript describes a new resource that I believe will be of great value and interest to the worldwide developmental biology community. The authors have carried out a very impressive systematic analysis of the expression patterns of all 19 Wnt ligands, all 10 frizzleds and the pattern of activation of Wnt/beta-catenin signalling in intact 3D mouse embryos at 3 key stages of development. Using a sophisticated analysis, they have systematically mapped regions of overlap between expression patterns of these components, thereby providing a highly detailed description of the complex and dynamic expression map. Wnt signalling regulates a plethora of different developmental processes in the embryo across a very wide range of tissues and has been widely studied for several decades, but this is the first systematic description of expression patterns of

© 2022. Published by The Company of Biologists under the terms of the Creative Commons Attribution License (https://creativecommons.org/licenses/by/4.0/).

genes that encode key components, and their relationship to each other. All of this data is freely available online in an easily accessible format. This type of highly detailed spatial expression analysis is an extremely valuable adjunct to the large amount of scRNAseq data now appearing in the literature.

Comments for the author

The manuscript is clear and well written, I have no substantial criticisms, but I would suggest that the authors address the following minor points.
1. Referring to Figure 2 (manuscript lines 99-114) the authors discuss regions of overlap and non-overlap between expression of Wnt ligand and frizzled receptors. I think that it is important to remember that Wnts are secretory proteins and have been found in long cytonemes which can project for considerable distances beyond the cell body, therefore the exact spatial distribution of active Wnt protein would be expected to be somewhat broader than the distribution of mRNA which is (presumably) mainly restricted to cell bodies. I think that this point should be included when describing/discussing Wnt/FZ co-localisation.
2. In supplementary movies 1,2 &3, please include a label (as in movies 4 & 5) so that the reader can immediately see which Wnt expression pattern the movie shows.
3. In Supp. Fig1 please add an arrow to indicate the location of the mis-mapped ectodermal Wnt6 expression.
4. In Figure 5, could the figure be amended by colour-coding the labels to show which colour signal corresponds to which Wnt gene (eg Wnt2/2b red/green)
5. Line 248. Please check the text carefully to make sure that the individual Wnt genes are correctly ascribed to Groups 1, 2 and 3 in Fig 6B. Specifically, the text describes Wnt1 as belonging to group 1 at E10.5 and E11.5, but the Figure indicates that this should be E9.5 and E11.5 – Wnt 1 is not present in the E10.5 diagram.
7. In panel 7C, include a key to indicate which developmental age that each coloured dot indicates (yellow blue, red)
8. In Figures 7E and F, please add labels for more anatomical landmarks to allow the reader to identify the tissue shown.
9. Line 347, typo, 'signalling'
10. Lines 442 & 458, typo 'complementary' (not 'complimentary')

<u>Reviewer 2</u>

Advance summary and potential significance to field

In "Integrated analysis of Wnt signalling system component gene expression", Murphy and colleagues have performed in situ hybridisation on all known Wnt genes as well as some Fzd and Tcf/Lef genes. Subsequently they mapped them back to a reference embryo. They provide these maps as a resource for the community.
The authors extract numerous statistics regarding overlap of expression domains of the genes, and have found that specific regions tend to contain expression of multiple genes.

This seems to constitute a very valuable resource and the manuscript and figures clearly report the general strategy to build it and conclusions that could be drawn from it. We are not able to evaluate the technical soundness of the approach as it lays beyond our area of expertise and is not described for a general audience.

Comments for the author

Below are some comments/questions:
l52: Could the authors elaborate on if Wnt has a particularly high number of paralogs compared to other similar morphogens? Is Wnt exceptional in this regard? Either way it may be good to know for the readers.
l72: Can the authors elaborate on what one can learn from areas where no Wnt is expressed?
l84: How standard is the mapping of expression pattern to a reference embryo? It would be useful here, but also in general, for a layman reader to know how straightforward this approach is.

© 2022. Published by The Company of Biologists under the terms of the Creative Commons Attribution License (https://creativecommons.org/licenses/by/4.0/).

l108: The authors sometimes use concrete statistics, and in other cases such as l121 don't mention the concrete percentage of the embryo were expression is more broad or contained. It would be useful for the reader a consistent use of percentages.
l131: Why are we interested in single gene domains? Are they evolutionarily new?
Is that a known/conserved mechanism?
l155: Is there an evolutionary reasoning to expect that Wnt and Frz ROHO overlap? Was this expected for regions of multiple ligands expression to overlap with regions of multiple receptor expression?
Do the overlap distances and groups in Figure 6B match with sequence similarity or any known evolutionary similarity?

First revision

Author response to reviewers' comments

We thank the Reviewers for their positive comments about the value of the paper and we are grateful for the interesting questions raised and the helpful clarifications suggested. We have edited and added to the manuscript according to each comment; Please see our detailed responses interspersed following each comment (in red text).

On the newly uploaded version. all additions to the manuscript are highlighted in red text; words removed are marked by strikethrough. During the process, a number of small errors were also corrected (e.g. detail on fig references) and indicated.

Reviewer 1 Advance Summary and Potential Significance to Field:
This manuscript describes a new resource that I believe will be of great value and interest to the worldwide developmental biology community. The authors have carried out a very impressive systematic analysis of the expression patterns of all 19 Wnt ligands, all 10 frizzleds and the pattern of activation of Wnt/beta-catenin signalling in intact 3D mouse embryos at 3 key stages of development. Using a sophisticated analysis, they have systematically mapped regions of overlap between expression patterns of these components, thereby providing a highly detailed description of the complex and dynamic expression map. Wnt signalling regulates a plethora of different developmental processes in the embryo across a very wide range of tissues and has been widely studied for several decades, but this is the first systematic description of expression patterns of genes that encode key components, and their relationship to each other. All of this data is freely available online in an easily accessible format. This type of highly detailed spatial expression analysis is an extremely valuable adjunct to the large amount of scRNAseq data now appearing in the literature.

Reviewer 1 Comments for the Author:
The manuscript is clear and well written, I have no substantial criticisms, but I would suggest that the authors address the following minor points.

1. Referring to Figure 2 (manuscript lines 99-114) the authors discuss regions of overlap and non-overlap between expression of Wnt ligand and frizzled receptors. I think that it is important to remember that Wnts are secretory proteins and have been found in long cytonemes which can project for considerable distances beyond the cell body, therefore the exact spatial distribution of active Wnt protein would be expected to be somewhat broader than the distribution of mRNA which is (presumably) mainly restricted to cell bodies. I think that this point should be included when describing/discussing Wnt/FZ co-localisation.

This is an important point and we have now added a passage to the discussion at line 376 as follows

Not surprisingly, regions of the embryo show extensive overlap in domains of Wnt and Fzd expression and canonical read-out. In relation to the proximity of Wnt mRNA signal to sites expressing Fzd receptor RNA or canonical readout, it is worth noting that in regions where Vangle2

© 2022. Published by The Company of Biologists under the terms of the Creative Commons Attribution License (https://creativecommons.org/licenses/by/4.0/).

is co-expressed (largely the nervous system) active Wnt protein may be present in long cytoneme processes extending from Wnt-expressing cells so that the signaling activity may be distant from cell bodies expressing Wnt mRNA (Brunt et al 2021).

We have additionally added a small caveat to a line in the discussion (line 402)

2. In supplementary movies 1,2 &3, please include a label (as in movies 4 & 5) so that the reader can immediately see which Wnt expression pattern the movie shows.

Done- thanks for the suggestion

3. In Supp. Fig1 please add an arrow to indicate the location of the mis-mapped ectodermal Wnt6 expression.

Done- thanks for the suggestion

4. In Figure 5, could the figure be amended by colour-coding the labels to show which colour signal corresponds to which Wnt gene (eg Wnt2/2b red/green)

Done- thanks for the suggestion

5. Line 248. Please check the text carefully to make sure that the individual Wnt genes are correctly ascribed to Groups 1, 2 and 3 in Fig 6B. Specifically, the text describes Wnt1 as belonging to group 1 at E10.5 and E11.5, but the Figure indicates that this should be E9.5 and E11.5 – Wnt 1 is not present in the E10.5 diagram.

Error has been corrected, thanks to the reviewer

7. In panel 7C, include a key to indicate which developmental age that each coloured dot indicates (yellow, blue, red)

Done- thanks for the suggestion

8. In Figures 7E and F, please add labels for more anatomical landmarks to allow the reader to identify the tissue shown.

There were originally two labels on part ii. We have enlarged these to make them more obvious and we have added more- thanks for the suggestion

9. Line 347, typo, 'signalling'

Done

10. Lines 442 & 458, typo 'complementary' (not 'complimentary')

Done

<u>Reviewer 2 Advance Summary and Potential Significance to Field:</u>

In "Integrated analysis of Wnt signalling system component gene expression", Murphy and colleagues have performed in situ hybridisation on all known Wnt genes as well as some Fzd and Tcf/Lef genes. Subsequently they mapped them back to a reference embryo. They provide these maps as a resource for the community. The authors extract numerous statistics regarding overlap of expression domains of the genes, and have found that specific regions tend to contain expression of multiple genes.
This seems to constitute a very valuable resource and the manuscript and figures clearly report the general strategy to build it and conclusions that could be drawn from it. We are not able to evaluate the technical soundness of the approach as it lays beyond our area of expertise and is not described for a general audience.

© 2022. Published by The Company of Biologists under the terms of the Creative Commons Attribution License (https://creativecommons.org/licenses/by/4.0/).

Reviewer 2 Comments for the Author: Below are some comments/questions:

l52: Could the authors elaborate on if Wnt has a particularly high number of paralogs compared to other similar morphogens? Is Wnt exceptional in this regard? Either way it may be good to know for the readers.

Wnt is indeed exceptional regarding the clear relationships between paralogous pairs of genes. We have added the following to the Introduction at line 42 to highlight this point.

Wnts are unusual with respect to the high number of family members and paired paralogues, compared for example to the hedgehog family, with only three vertebrate members. FGFs are another example of a large family of signaling-molecule-encoding-genes present early in multicellular evolution, like Wnts; however, their classification into direct paralogous pairs is less clear (Itoh and Ornitz, 2011)

l72: Can the authors elaborate on what one can learn from areas where no Wnt is expressed?

We have added the following passage to Discussion line 362

Given the dynamism of the patterns, it is possible that a region where there is no detected expression at one stage might show expression at another, however there is clearly some consistency in the regions that show no detected expression across the three stages assessed here.

l84: How standard is the mapping of expression pattern to a reference embryo? It would be useful here, but also in general, for a layman reader to know how straightforward this approach is.

Indeed, it is very important to communicate this effectively. An accompanying manuscript describing the WlzWarp process for the mapping in more detail is cited in the current paper and to make this clearer we have added an extra citation to the first sentence in the results (line 91).
In addition, we have added a comment to the methodology at line 532 as follows

The process is straightforward with the key required competence being an understanding of the biology and anatomy rather than technical IT expertise. More detail is provided by Hill et al, 2022, but the mappings for this data required operator time per embryo of about 60 minutes.

And we have added a statement to the introduction to underline the novelty of the spatial mapping to model embryos- at line 66 as follows:

Critically, spatial mapping onto an explicit coordinate model embryo enables exploration and analysis of the underlying molecular anatomy unbiased by anatomical interpretation based on histology as has been demonstrated by numbers of projects including the comprehensive Allen Brain Atlas (Lein et al., 2007).

l108: The authors sometimes use concrete statistics, and in other cases such as l121 don't mention the concrete percentage of the embryo were expression is more broad or contained. It would be useful for the reader a consistent use of percentages.

The difference in type of statement (qualitative or quantitative) is related to the type of analysis being reported in the respective paragraphs. The former paragraph is speaking to Figure 2A which shows the respective domains visually in snap shots of the mapped domains. The following paragraph moves on to presenting the reader with another type of analysis unique to this approach- namely quantitative analysis of the digitally mapped domains. This speaks to the data presented in Figure 2B and, importantly, also in Supplementary data tables S1 and S2.

We have added the word “quantitatively” to the opening sentence in the latter paragraph (line 121) to help make this clearer.

© 2022. Published by The Company of Biologists under the terms of the Creative Commons Attribution License (https://creativecommons.org/licenses/by/4.0/).

l131: Why are we interested in single gene domains? Are they evolutionarily new? Is that a known/conserved mechanism?

The reviewer raises an interesting question which we attempted to address in the discussion but upon rereading we realise it is too concise to come across clearly. We have elaborated a little on this point (line 370) to highlight the two aspects we suggest are most important: 1) that unique expression domains might indicate territories of unique function for that gene and therefore be reflected in strong phenotypes upon inactivation; and 2), as the reviewer picks out, that it might reflect expression territories more recently added evolutionarily – neofunctionalization. Because the paper is already very long, we attempt to clarify this concisely by slightly expanding the current text. We have removed "and regulation" (which was intended to cover point 2) and have added the following sentence to expand slightly.

"*Domains where only one Wnt is expressed may also represent evolutionary diversification involving changes in gene regulation, more recently evolved territories of expression and neofunctionalization (Ohno, 1970).*

l155: Is there an evolutionary reasoning to expect that Wnt and Frz ROHO overlap? Was this expected for regions of multiple ligands expression to overlap with regions of multiple receptor expression?

We did not predict that the *Wnt* and *Fzd* ROHOs would overlap. Very few studies have explored the *in situ* expression patterns of both *Wnt* and *Fzd* gene families, and this approach was necessary to discover the overlap between regions of multiple *Wnt* gene expression with regions of multiple *Fzd* gene expression in the developing mouse embryo. From an evolutionary perspective, there is clear value in an overlap between *Wnt* signal and *Fzd* receptor gene expression, as expression of both *Wnt* and *Fzd* are necessary to ensure robust Wnt Signaling Pathway activation. For future work, it would be interesting to explore whether the overlap between *Wnt* and *Fzd* ROHO domains may highlight undiscovered *cis*- or *trans*-regulatory networks that ensure coexpression of *Wnt* and *Fzd* genes in distinct regions / compartments of the mouse embryo.

Do the overlap distances and groups in Figure 6B match with sequence similarity or any known evolutionary similarity?

The network analysis Group 3 genes include Wnt5a*, Wnt5b, Wnt8a, Wnt8b, Wnt9a, Wnt9b, Wnt10a, Wnt10b,* and *Wnt2b*. The paralogous pairs included in this group show sequence similarity and this relates to an ancestral genome duplication event. The expression patterns of the paralogous pairs in this group are overlapping but distinct, and it is possible that common *trans*-regulatory factors are driving the co-expression of these genes, as explored in the part of the paper focused on comparison of paralogous pairs.

The network analysis Group 2 genes include *Wnt11, Wnt16* and *Wnt2*. Of these, *Wnt2* and *Wnt16* are both linked on chromosome 6 and so there is the distinct possibility that a common *cis*-acting enhancer element is regulating expression of both of these genes.

The network analysis Group 1 genes includes *Wnt7a, Wnt7b, Wnt3, Wnt3a, Wnt4* at all three stages, and *Wnt1* at E10.5 and E11.5. As with the Group 3 genes, it is possible that common *trans*-regulatory factors are driving the co-expression of the paralogous pairs in Group 1. It is additionally noteworthy that *Wnt3* and *Wnt3a* are both on chromosome 11, however they are >44.5 Mbp apart and thus unlikely to be co-regulated by a common *cis*-acting enhancer element. Consequently, there is no simple correlation between linkage and the Group 1 set of *Wnt* genes.

To concisely cover this interesting question in the manuscript we have added the following passage to line 440 (Discussion)

Indeed, there is no obvious simple relation between the net similarity of expression of pairs of genes represented in Figure 6Bi and either their phylogenetic relationships based on DNA sequence (Somorjai et al 2018), or certain widely conserved, tight chromosomal linkages (between Wnts1 and 10b, 6 and 10a, 3a and 9a, 3 and 9b (Ensemble genome browser https://www.ensembl.org/index.html). The potential for evolutionary conservation and shuffling

© 2022. Published by The Company of Biologists under the terms of the Creative Commons Attribution License (https://creativecommons.org/licenses/by/4.0/).

of cis or trans regulatory modules, perhaps controlling different parts of each pattern (as for example, in Fig. 6Bii), may be a fruitful area for future investigation.

Second decision letter

MS ID#: DEVELOP/2021/200312

MS TITLE: Integrated analysis of Wnt signalling system component gene expression

AUTHORS: Paula Murphy, Chris Armit, Bill Hill, Shanmugasundaram Venkataraman, Patrick Frankel, Richard A Baldock, and Duncan R Davidson
ARTICLE TYPE: Research Article

I am happy to tell you that your manuscript has been accepted for publication in Development, pending our standard ethics checks.

Reviewer 1

Advance summary and potential significance to field

This paper provides a thorough and comprehensive description of multiple components of the Wnt signalling pathway at key stages of mouse development, I believe that this will be an extremely useful resource to the developmental biology community.

Comments for the author

I confirm that the authors have fully responded to my concerns and I would be happy to see the manuscript published with no further changes.

Reviewer 2

Advance summary and potential significance to field

The authors seem to have addressed all of the concerns that were raised.

Comments for the author

The authors seem to have addressed all of the concerns that were raised.

© 2022. Published by The Company of Biologists under the terms of the Creative Commons Attribution License (https://creativecommons.org/licenses/by/4.0/).
